# Assessing the prevalence of young children living in households prepared for COVID-19 in 56 low- and middle-income countries

**DOI:** 10.1186/s41256-022-00254-2

**Published:** 2022-06-21

**Authors:** Chunling Lu, Yiqun Luan, Sara N. Naicker, S. V. Subramanian, Jere R. Behrman, Jody Heymann, Alan Stein, Linda M. Richter

**Affiliations:** 1 Division of Global Health Equity, Brigham & Women’s Hospital, Boston, MA USA; 2 Department of Global Health & Social Medicine, Harvard Medical School, Boston, MA USA; 3 The Heller School for Social Policy and Management, Brandeis University, Waltham, MA USA; 4 DSI-NRF Centre of Excellence in Human Development, University of Witwatersrand, Johannesburg, South Africa; 5 Harvard Center for Population and Development Studies, Cambridge, MA USA; 6 Department of Social and Behavioral Sciences, Harvard T.H. Chan School of Public Health, Boston, MA USA; 7 Center for Geographic Analysis, Harvard University, Cambridge, MA USA; 8 Department of Economics, University of Pennsylvania, Philadelphia, PA USA; 9 WORLD Policy Analysis Center, University of California, Los Angeles, Los Angeles, CA USA; 10 Department of Psychiatry, University of Oxford, Oxford, UK; 11 MRC/Wits Rural Public Health and Health Transitions Research Unit (Agincourt), Faculty of Health Sciences, School of Public Health, University of the Witwatersrand, Johannesburg, South Africa; 12 Africa Health Research Institute, Durban, KwaZulu-Natal South Africa

**Keywords:** Child, Child development, Developing Countries, Preparedness, COVID-19, Pandemics, Communicable diseases, Quarantine, Hand hygiene, Toilet facilities, Mass media, Telephone

## Abstract

**Background:**

The COVID-19 pandemic and governments’ attempts to contain it are negatively affecting young children’s health and development in ways we are only beginning to understand and measure. Responses to the pandemic are driven largely by confining children and families to their homes. This study aims to assess the levels of and associated socioeconomic disparities in household preparedness for protecting young children under the age of five from being exposed to communicable diseases, such as COVID-19, in low- and middle-income countries (LMICs).

**Methods:**

Using data from nationally representative household surveys in 56 LMICs since 2016, we estimated the percentages of young children under the age of five living in households prepared for communicable diseases (e.g., COVID-19) and associated residential and wealth disparities at the country- and aggregate-level. Preparedness was defined on the basis of space for quarantine, adequacy of toilet facilities and hand hygiene, mass media exposure at least once a week, and phone ownership. Disparities within countries were measured as the absolute gap in two domains—household wealth and residential area - and compared across regions and country income groups.

**Results:**

The final data set included 766,313 children under age five. On average, 19.4% of young children in the 56 countries lived in households prepared for COVID-19, ranging from 0.6% in Ethiopia in 2016 to 70.9% in Tunisia in 2018. In close to 90% of countries (50), fewer than 50% of young children lived in prepared households. Young children in rural areas or in the poorest households were less likely to live in prepared households than their counterparts.

**Conclusions:**

A large portion of young children under the age of five in LMICs were living in households that did not meet all preparedness guidelines for preventing COVID-19 and caring for patients at home. This study highlights the need to ensure all families in LMICs have the means to prevent the spread of the pandemic or other communicable illnesses to young children during pandemics.

**Supplementary Information:**

The online version contains supplementary material available at 10.1186/s41256-022-00254-2.

## Introduction

The COVID-19 pandemic has affected every aspect of our lives and imposed threats on people in every age group. Young children, especially those in low-income households, are very vulnerable to the pandemic and governmental actions to contain it. Mounting evidence demonstrates a wide range of negative impacts on young children’s health, protection, learning, and development [1], as a consequence of day care, preschool, and school closures [2]; routine health care service interruptions, including vaccination, prenatal and postnatal checkups [3, 4]; increased poverty and malnutrition due to losses of employment and income in households [5]; increased neglect and maltreatment resulting from caregiver stress [6, 7]; and isolation of children from adults who can identify and report child maltreatment [8].

The prevalence of COVID-19 among young children has been reported to be low [9]. This low rate could be due to the high prevalence of asymptomatic cases among young children which has kept them from being identified and tested [10]. Though clinical studies have found that most children who test positive for COVID-19 had no or mild symptoms [11], children with comorbidities, such as cardiac conditions or respiratory disease, accounted for a large proportion of the few who became critically ill [12]. Moreover, newly emerging evidence suggests that children with asymptomatic or mild symptoms may also develop long-term symptoms such as fatigue, muscle and joint pain, and respiratory problems [13].

Governments in many countries adopted confinement to homes as the primary mechanism to contain the spread of the virus. The World Health Organization (WHO) and countries’ Ministry of Health or Center for Disease Control (CDC) offices advised implementing home quarantines for people who had contact with infected people or experienced mild/moderate symptoms of COVID-19 [14–17]. Never before in recent history have so many people been confined to their homes and prevented from moving freely for the sake of their own and others’ health. For many households in low- and middle-income countries (LMICs), the measures put into place for COVID-19 prevention and control—movement restrictions, home confinement, handwashing with water and soap, closure of schools and workplaces—are beyond their resources, and the restrictions thus potentially harmful to their wellbeing [18]. In addition, lack of preparedness and lockdown conditions may affect children’s nurturing care, including their health, nutrition, security and safety, early learning, and responsive caregiving, with long-lasting effects on their development that may be difficult to compensate for, especially under impoverished conditions [19].

Given the global commitments to fighting communicable diseases and reducing preventable morbidity and mortality for children under the age of five, as stated in the Sustainable Development Goals [20], there is an urgency to examine how many and to what extent LMICs households were prepared for protecting household members, including young children, from the spread of communicable diseases, such as COVID-19. Though the world has responded to COVID-19 by forming various global initiatives to prepare for or monitor the development of cutting-edge biomedical technologies (e.g., testing, vaccine, and treatment) and vaccine purchase and distribution to LMICs [21], little attention has been paid to improving household and community socioeconomic infrastructure in LMICs, including housing, water, sanitation, and access to necessary information, that could serve as a fundamental means to close socioeconomic gaps on young children’s health and development and prepare households to mitigate future pandemic impacts on young children’s developmental trajectories. To provide scientific evidence for policymakers and other stakeholders, we used nationally representative household survey data since 2016 from 56 LMICs to conduct the first comprehensive assessment of the prevalence of young children under the age of five living in households prepared for communicable diseases spread by droplets and bodily fluids, such as the COVID-19, and associated residential and wealth disparities at both the country- and aggregate-level.

## Methods

### Definition

The definition of prepared households is based on guidelines on preparing for COVID-19 at home from WHO and countries’ Ministry of Health or CDC offices [14–17]. These guidelines offer recommendations to households on readiness for COVID-19, especially on how to protect members from those with mild and moderate conditions or to quarantine patients or those who had contact with infected people. For example, ideally, households should have rooms for quarantine and basic sanitation to avoid direct contact with patients’ excrement. Household members should take preventive actions, including frequent handwashing with water and soap and cleaning and disinfecting frequently touched surfaces. In addition, it is important that households stay informed about updates on COVID-19-related education and social issues, maintain good communication with health and other care providers, and have emergency contacts available. These requirements are usually applied to the communicable diseases caused by droplets and bodily fluids. Based on these requirements, this study defines a household prepared for communicable diseases, such as COVID-19, as one that meets five conditions: (a) space for quarantine (≤ three persons per sleeping room) [22, 23], (b) basic sanitation, (c) soap and water available for handwashing, (d) phone for communication, and (e) weekly exposure to mass media.

### Data source

To gather household surveys with available variables indicating these five conditions, we searched the International Household Survey Network (IHSN), a website that provides a list of household surveys conducted in LMICs [24]. Our search yielded Demographic and Health Surveys (DHS) [25] and Multiple Indicator Cluster Surveys (MICS) [26] that have variables covering all five conditions. Details on the data search are presented in Chapter 1 of Additional file 1.

Both DHS and MICS are nationally representative household surveys that provide a wide range of variables on LMICs household socioeconomic characteristics and collected representative data for children under the age of five. The two surveys are highly comparable due to their similar sampling design, implementation procedure, and measurement strategies [27]. They have been regularly used together in tracking progress in child and maternal health by researchers as well as international and national agencies [28–31]. Both DHS and MICS surveys follow a two-stage, stratified cluster sampling approach. Typically, before sampling, a country’s samples are stratified by geographic region and further by urban/rural areas. Within each stratum, the first sampling stage selects clusters (always census enumeration areas) with probability proportional to the contribution of that cluster’s population to the total population, while the second stage randomly selects households from a complete household listing of each selected cluster by equal probability [32, 60].

Evidence shows that poorer households are more likely to have higher fertility rates with more young children than better-off households, especially in low-income countries [33]. Assuming that the prevalence of households with preparedness for COVID-19 is the same as the prevalence of young children living in prepared households could lead to an underestimation of young children living in homes with poor preparedness for COVID-19. Therefore, this study focuses on the proportion of young children under the age of five that live in households with preparedness for communicable diseases such as COVID-19.

For each country, we included the most recent data since 2016. This decision balances a tradeoff between data currency for the immediate pre-COVID-19 period and data availability: only 19 countries had data available since 2019 – the year that COVID-19 started. To include more countries in the analysis, we followed practice in previous studies [34] and extended the timeline to 2016. Our final sample includes 56 countries, with 20 including data between 2016 and 2017, and 36 since 2018. Among them, 21 were low-income, 25 lower-middle-income, and ten upper-middle-income countries, according to World Bank 2015 country income classifications (Additional file 1: Table S2) [35]. Our analysis included 766,313 young children under five years of age.

### Variable measurement

#### Measuring a household with preparedness for communicable diseases such as COVID-19

We measured the percentages of young children under the age of five living in households with preparedness for communicable diseases such as COVID-19 by constructing five binary variables indicating if young children lived in households that met all five conditions described above. Sample questions for these five conditions are presented in Additional file 1: Table S3. Details on how we constructed the five binary variables are presented in Chapter 1 of Additional file 1.

Using the five binary variables on household conditions for home prevention and care, we constructed a binary summary measure, coded as 1 if a child lived in a household that was positive for *all* five binary variables, and 0 otherwise. The percentage of young children under the age of five living in households with preparedness for COVID-19 was calculated from young children living in prepared households as the numerator, and all young children under age five as the denominator. Households with missing values in any of the five variables were excluded from the analysis based on recommendations by the DHS and MICS programs and previous practice [36–38]. Percentages of missingness of these variables in each country are presented in Additional file 1: Table S4.

#### Measuring inequalities by residential area and wealth quintile

Inequality was measured by the difference in percentages of young children under the age of five living in prepared households by residential area (urban vs. rural) or wealth (highest vs. lowest wealth quintiles). A greater than zero difference indicated that young children living in rural areas or in the lowest wealth quintile had lower percentages of preparedness than their counterparts, and vice versa.

When testing for inequalities between the two groups, we used logistic regression with a dichotomous indicator for preparedness as the dependent variable and urban or wealth indicators as independent variables (more details in Chapter 1 of Additional file 1). We used two-tailed tests and statistical significance at *p* < 0.05 to examine the residential and wealth disparities in the prevalence of children living in prepared households.

For wealth disparities, the wealth index in DHS and MICS is a composite measure of households’ ownership of various types of assets or services, including phones, sanitation, and water. In published DHS and MICS country reports [37, 39], when reporting wealth gaps in sanitation, for example, an existing wealth index is used even if sanitation is also part of the wealth index. Previous studies exploring the wealth gap (poorest vs. richest) in sanitation compared the existing wealth index to one without sanitation [40–42]. These studies found high correlations between the two wealth indices and a somewhat narrowed wealth gap after excluding sanitation from the wealth index. However, the change in the wealth gap decreased as the number of assets included in the wealth index increased. These studies suggested that because DHS and MICS use large numbers of asset or service indicators in constructing wealth measures, the existing wealth index could be used for wealth gap assessment for the assets or services included in the wealth index. Furthermore, a recent study validated that excluding asset variables, such as smartphones, from wealth index construction was likely to skew the wealth index [43]. Considering country practice and evidence on the pros and cons of constructing a new wealth index without including related asset or service variables, we decided to use the existing wealth quintile variable for inequality assessment.

### Statistical analysis

We assessed the prevalence of young children under the age of five living in households prepared for communicable diseases, such as COVID-19, in 56 countries and its associated residential and wealth disparities at both the country- and aggregate-level. At the country level, we followed the DHS and MICS guidelines and adjusted for estimation with sampling weights, clustering, and stratification variables [44, 60]. We conducted the same analysis for each of the five conditions that defined household preparedness, as these conditions represent different dimensions of preparedness. We believe that, for policymakers and other stakeholders, knowing which condition did poorly would assist in identifying problems and improving the related capacity.

Our aggregate-level analysis generated average prevalence across all 56 countries as well as by country income groups and regions. We followed previous studies and used random-effects meta-analysis, combined with the DerSimonian and Laird inverse-variance method, to generate average estimates across countries [45, 46]. This approach assumed heterogeneity among the estimates across countries [47], and we tested this assumption with more details presented in Chapter 1 of Additional file 1.

To conduct sensitivity tests, we repeated the above analyses by varying thresholds for adequate quarantine conditions with two persons per sleeping room (lower bound) and four persons per sleeping room (upper bound).

### Ethical clearance

Ethical approval was not applicable to this study as the data used are secondary data and are publicly downloadable in anonymized form.

## Results

Using the most recent surveys from the 56 LMICs since 2016, we included 766,313 young children under the age of five (69.4% living in rural areas; 26.2% living in households in the lowest wealth quintile) in the analysis, noting that there are more young children in the poorest households.

### Prevalence of young children under age five living in households with preparedness

On average, only 19.4% (95% CI, 17.1–21.8%) of young children in 56 countries lived in prepared households, with the lowest percentage in sub-Saharan Africa (4.6%, 95% CI, 3.9–5.4%). In low-income countries, only 4.4% (95% CI, 3.5–5.4%) of young children lived in prepared households, compared to 24.5% (95% CI, 19.3–29.7%) in lower-middle-income and 38.7% (95% CI, 26.4–51.0%) in upper-middle-income countries (Table 1). Among the five conditions of preparedness, on average, 84.0% of young children lived in households with phones (95% CI, 82.0–86.0%), 63.2% with adequate quarantine conditions (95% CI, 59.4–67.1%), 61.5% with mass media exposure at least once a week (95% CI, 55.3–67.7%), 50.3% with basic sanitation (95% CI, 41.1–59.4%), and 48.7% with basic hygiene (95% CI, 39.3–58.1%). In sub-Saharan Africa and in low-income countries, fewer than 30% of young children lived in households with basic sanitation and hygiene facilities (Table 1). Country-level estimates for each condition are in Additional file 1: Tables S8, S10, S12, S14, and S16.

**Table 1:** Aggregate-level prevalence of young children living in households with preparedness and each condition

	No. of countries	Prepared households, % (95% CIs)	Adequate quarantine, % (95% CIs)	Basic hygiene, % (95% CIs)	Basic sanitation, % (95% CIs)	Ownership of phones, % (95% CIs)	Mother weekly exposed to mass media, % (95% CIs)
Average	56	19.4(17.1, 21.8)	63.2(59.4, 67.1)	48.7(39.3, 58.1)	50.3(41.1, 59.4)	84.0(82.0, 86.0)	61.5(55.3, 67.7)
*Region*							
East Asia and Pacific	11	23.5(13.7, 33.2)	56.1(45.5, 66.7)	67.9(55.4, 80.4)	64.0(51.7, 76.4)	86.5(82.6, 90.4)	65.2(53.8, 76.6)
Europe and Central Asia	3	52.9(38.4, 67.5)	75.0(59.5, 90.4)	89.1(79.1, 99.0)	91.6(85.5, 97.7)	92.8(86.8, 98.8)	91.5(86.3, 96.7)
Latin America and the Caribbean	5	35.8(14.1, 57.5)	70.9(61.5, 80.3)	65.2(39.4, 91.0)	70.8(50.0, 91.6)	90.7(86.0, 95.5)	81.4(72.2, 90.6)
Middle East and North Africa	4	50.8(38.4, 63.2)	66.1(54.4, 77.7)	92.9(89.7, 96.1)	91.5(85.9, 97.2)	97.5(96.1, 98.9)	88.3(81.6, 94.9)
South Asia	5	32.1(21.6, 42.6)	60.4(45.2, 75.6)	73.4(56.3, 90.5)	67.8(41.5, 94.0)	96.2(93.2, 99.1)	65.7(53.5, 77.9)
Sub-Saharan Africa	28	4.6(3.9, 5.4)	63.4(58.6, 68.2)	23.1(18.4, 27.8)	27.7(22.3, 33.1)	76.6(71.1, 82.2)	48.8(40.3, 57.3)
*Country income class*							
Low-income	21	4.4(3.5, 5.4)	64.7(58.8, 70.7)	22.0(15.4, 28.6)	26.6(19.4, 33.7)	73.5(66.4, 80.6)	45.4(36.9, 53.9)
Lower-middle income	25	24.5(19.3, 29.7)	60.4(54.4, 66.4)	60.8(51.1, 70.5)	59.2(47.5, 70.8)	89.3(87.6, 91.1)	66.1(58.0, 74.1)
Upper-middle income	10	38.7(26.4, 51.0)	67.1(59.9, 74.3)	74.3(60.4, 88.2)	77.7(68.3, 87.0)	93.0(90.3, 95.6)	84.3(79.9, 88.7)

**Fig. 1:**
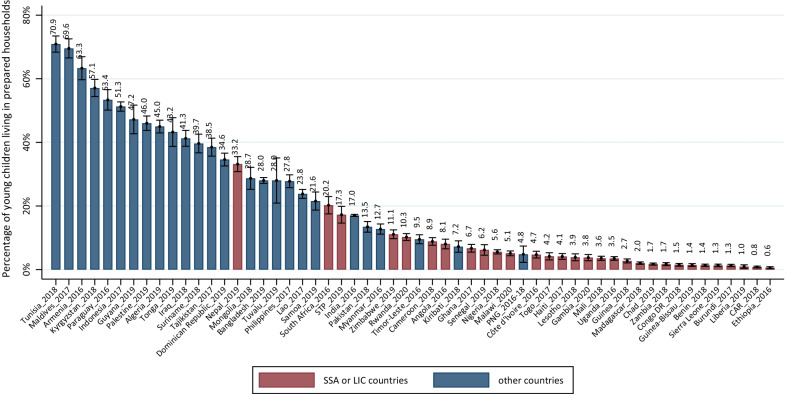
Country-level percentage (%) of young children living in households with preparedness

Country-level preparedness ranged from 0.6% (95% CI, 0.3%-0.9%) in Ethiopia in 2016 to 70.9% (95% CI, 68.4%-73.4%) in Tunisia in 2018 (Fig. 1; Additional file 1: Table S6). In only six countries (all middle-income), more than 50% of young children lived in prepared households: Tunisia (70.9%), Maldives (69.6%), Armenia (63.3%), Kyrgyzstan (57.1%), Paraguay (53.4%), and Indonesia (51.3%). In 34 countries, mostly in sub-Saharan Africa (27 countries), fewer than 20% of young children lived in prepared households, including all 21 low-income countries except for Nepal (33.2%), 13 lower-middle-income, and one upper-middle-income country (Angola 8.1%). Countries with the lowest estimates are all from sub-Saharan Africa, including Ethiopia (0.6%), Central African Republic (0.8%), Liberia (1.0%), Burundi (1.3%), Sierra Leone (1.3%), Benin (1.4%), Guinea-Bissau (1.4%), Democratic Republic of the Congo (1.5%), and Zambia (1.7%) (Fig. 1; Additional file 1: Table S6).

### Residential area-associated disparities

The average urban–rural gap across the 56 LMICs was 10.3 percentage points (pp) (95% CI, 8.5–12.1 pp) (Table 2). Low-income countries had the lowest gap (5.6 pp) because of relatively low percentages of urban children living in prepared households. The same explanation applies to the small gap observed in sub-Saharan Africa (6.8 pp). Among the five conditions, on average, mother’s weekly mass media exposure had the largest urban–rural gap (20.3 pp), followed by basic sanitation (15.2 pp), basic hygiene (13.6 pp), phone ownership (12.9 pp), and adequate quarantine conditions (6.7 pp) (Table 2). Details on country-level residential area-associated disparities in preparedness and each condition are in Additional file 1: Tables S6, S8, S10, S12, S14, and S16.

**Table 2:** Aggregate-level residential disparities in the prevalence of young children living in households with preparedness and each condition

	No of countries	Prepared households, % (95% CIs)	Adequate quarantine, % (95% CIs)	Basic hygiene, % (95% CIs)	Basic sanitation, % (95% CIs)	Ownership of phones, % (95% CIs)	Mother weekly exposed to mass media, % (95% CIs)
Average	56	10.3(8.5, 12.1)	6.7(5.2, 8.3)	13.6(10.7, 16.5)	15.2(11.8, 18.5)	12.9(11.1, 14.7)	20.3(16.6, 24.1)
*Region*							
East Asia and Pacific	11	14.4(8.9, 20.0)	8.0(2.2, 13.8)	15.0(9.9, 20.1)	16.6(8.9, 24.3)	12.4(8.2, 16.5)	17.4(10.1, 24.6)
Europe and Central Asia	3	17.7(-0.2, 35.6)	4.4(-1.4, 10.3)	7.5(0.7, 14.3)	15.8(-1.3, 32.9)	1.8(0.2, 3.4)	1.9(0.4, 3.3)
Latin America and the Caribbean	5	12.8(5.3, 20.3)	7.8(1.0, 14.6)	8.8(3.6, 14.1)	15.4(7.7, 23.0)	7.2(3.5, 10.9)	12.7(6.0, 19.5)
Middle East and North Africa	4	10.5(3.1, 17.9)	6.5(2.3, 10.8)	5.6(1.5, 9.6)	6.1(1.5, 10.8)	0.6(0.0, 1.3)	1.9(0.7, 3.0)
South Asia	5	15.1(10.7, 19.4)	7.9(3.5, 12.4)	19.1(7.6, 30.5)	14.7(-2.9, 32.2)	2.7(-0.2, 5.7)	22.1(12.2, 31.9)
Sub-Saharan Africa	28	6.8(5.6, 8.0)	6.1(4.0, 8.2)	14.6(11.8, 17.4)	16.0(12.7, 19.3)	19.2(14.9, 23.5)	27.2(22.2, 32.1)
*Country income class*							
Low-income	21	5.6(4.6, 6.7)	6.6(4.1, 9.0)	13.3(10.2, 16.4)	15.1(11.5, 18.6)	20.6(15.5, 25.7)	26.4(22.0, 30.9)
Lower-middle income	25	12.9(9.7, 16.1)	6.2(3.9, 8.5)	15.6(10.5, 20.7)	16.1(10.2, 22.0)	8.7(6.8, 10.7)	19.0(13.0, 24.9)
Upper-middle income	10	14.2(11.1, 17.3)	8.6(4.6, 12.6)	8.9(4.6, 13.2)	13.2(6.5, 19.8)	7.7(3.7, 11.7)	11.0(4.0, 18.0)

**Fig. 2:**
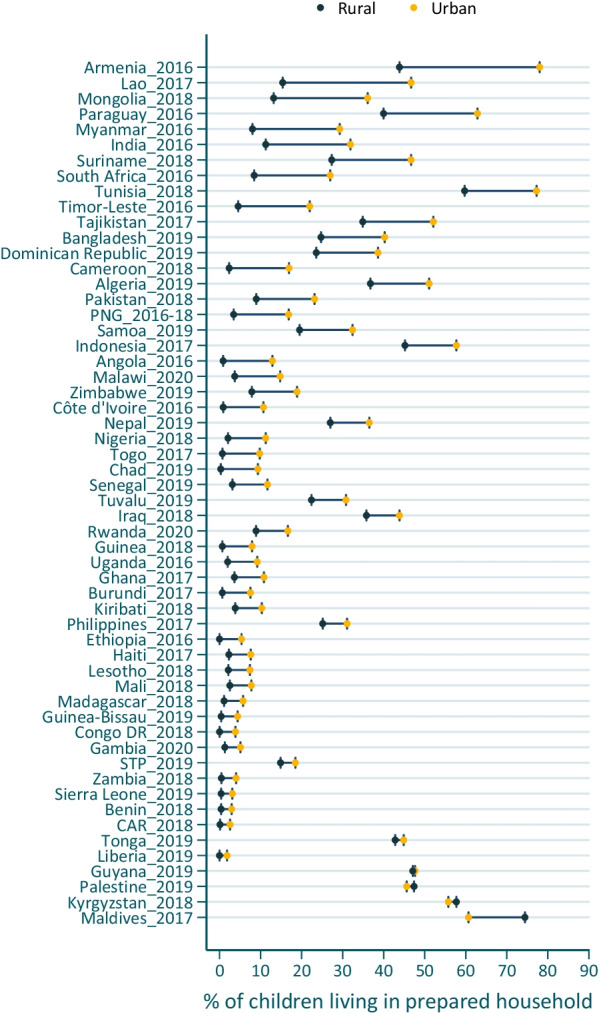
Country-level residential disparities in percentage (%) of young children living in households with preparedness

At the country level, the preparedness urban–rural gap favored urban children and was statistically significant (*p* < 0.05) in 49 of 56 countries (88%), ranging from 1.8 pp in Liberia in 2019 to 34.2 pp in Armenia in 2016. In many LMICs, large portions of both urban and rural children were living in households with poor preparedness, leading to small urban–rural gaps (e.g., Benin, urban children 3.0% and rural children 0.4%). In low-income countries (except for Nepal), fewer than 20% of urban children lived in prepared households (Fig. 2, Additional file 1: Table S6 for details).

### Wealth-associated disparities

The average gap between young children in the richest quintile households and those in the poorest quintile was 33.5 pp, with the largest gap in Latin America and the Caribbean (59.4 pp) due to high inequality in the two upper-middle-income countries (Dominican Republic and Paraguay), and the smallest in sub-Saharan Africa (19.6 pp). Low-income countries had much smaller gaps than countries in the other two income groups, because in low-income countries, even children in the richest quintile lived in households with low levels of preparedness (Table 3). Regarding the five conditions of preparedness, basic sanitation had the largest average gap (44.1 pp), followed by mother’s weekly exposure to mass media (41.8 pp), basic hygiene (33.6 pp), phone ownership (31.9 pp), and adequate quarantine conditions (26.5 pp). Details on country-level wealth-associated disparities in preparedness and each condition are in Additional file 1: Tables S7, S9, S11, S13, S15, and S17.

**Table 3:** Aggregate-level wealth disparities in the prevalence of young children living in households with preparedness and each condition

	No. of countries	Prepared households, % (95% CIs)	Adequate quarantine, % (95% CIs)	Basic hygiene, % (95% CIs)	Basic sanitation, % (95% CIs)	Ownership of phones, % (95% CIs)	Mother weekly exposed to mass media, % (95% CIs)
Average	56	33.5(27.0, 40.1)	26.5(22.9, 30.1)	33.6(26.3, 41.0)	44.1(34.7, 53.5)	31.9(25.7, 38.1)	41.8(34.3, 49.3)
*Region*							
East Asia and Pacific	11	48.7(38.1, 59.2)	36.1(25.1, 47.1)	39.7(29.8, 49.5)	53.6(41.8, 65.4)	31.4(18.5, 44.3)	34.6(19.8, 49.4)
Europe and Central Asia	3	32.0(4.7, 59.2)	10.1(-0.4, 20.5)	16.6(-0.7, 34.0)	20.9(1.4, 40.4)	6.2(1.1, 11.2)	7.8(3.0, 12.6)
Latin America and the Caribbean	5	59.4(33.2, 85.7)	40.5(32.4, 48.5)	34.5(15.6, 53.3)	49.5(37.2, 61.9)	23.1(8.8, 37.4)	28.1(13.8, 42.3)
Middle East and North Africa	4	45.2(34.6, 55.9)	32.9(24.6, 41.2)	16.3(8.5, 24.0)	16.5(5.3, 27.8)	5.4(2.4, 8.5)	9.3(3.6, 15.1)
South Asia	5	44.3(34.7, 54.0)	29.9(20.8, 39.1)	51.4(28.7, 74.1)	44.0(6.6, 81.3)	10.2(0.1, 20.3)	54.5(37.8, 71.1)
Sub-Saharan Africa	28	19.6(15.9, 23.2)	20.8(17.1, 24.4)	32.1(26.5, 37.8)	46.0(38.8, 53.2)	44.0(32.2, 55.9)	53.1(45.8, 60.4)
*Country income class*							
Low-income	21	14.2(11.4, 17.0)	20.2(16.3, 24.1)	27.3(21.8, 32.8)	39.4(32.4, 46.3)	47.6(32.9, 62.2)	52.4(45.9, 58.9)
Lower-middle income	25	41.6(33.4, 49.7)	29.1(23.3, 34.9)	39.4(26.9, 51.9)	49.1(33.3, 64.9)	24.5(18.5, 30.5)	39.9(28.1, 51.7)
Upper-middle income	10	54.2(42.6, 65.8)	34.2(27.4, 41.0)	32.4(18.3, 46.5)	41.8(25.6, 57.9)	17.3(8.1, 26.5)	24.5(9.4, 39.6)

**Fig. 3:**
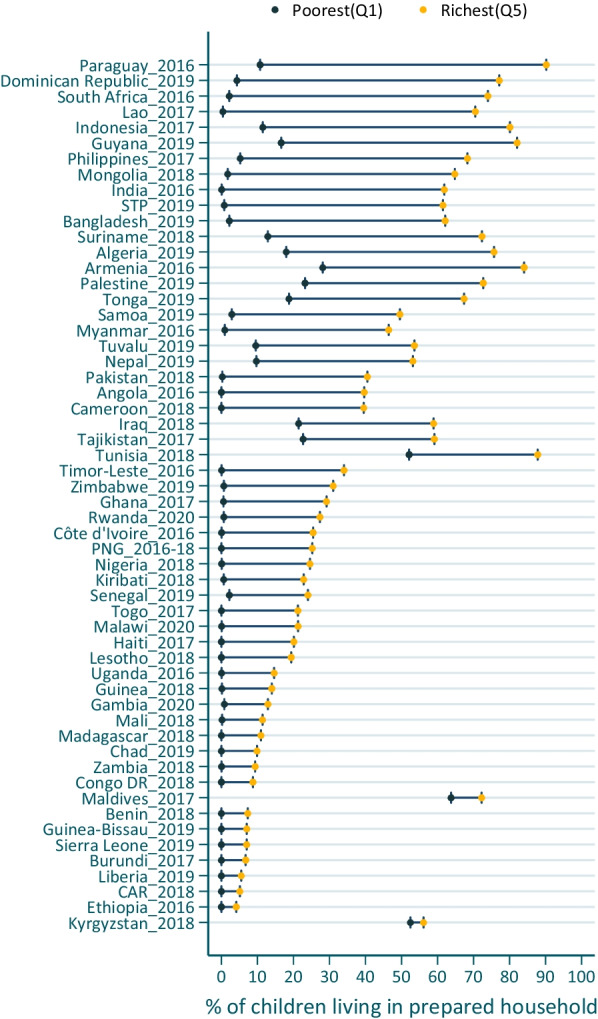
Country-level wealth disparities in percentage (%) of young children living in households with preparedness

At the country level, 54 out of 56 countries, except Kyrgyzstan and Maldives, had significant richest-poorest gaps, favoring children in the richest quintile. These gaps ranged from 4.1 pp (95% CI, 2.0–6.2 pp) in Ethiopia in 2016 to 79.4 pp (95% CI, 75.3–83.6 pp) in Paraguay in 2016. In most low-income countries, the richest quintiles had fewer than 20% of children living in prepared households, leading to smaller wealth gaps in these countries. For example, in Ethiopia, only 4.1% of children in the richest quintile lived in prepared households. In 34 of 56 countries, fewer than 1% of young children in the poorest quintile lived in prepared households (Fig. 3, Additional file 1: Table S7 for details).

### Sensitivity analysis

Sensitivity tests using the lower- (2-persons per sleeping room) or upper-bound (4-persons per sleeping room) threshold for adequate quarantine conditions yielded consistent results (Additional file 1: Tables S18, S19 and S20). On average, fewer than 10% or 24.1% of young children lived in prepared households in the 56 countries when using 2-person or 4-person per sleeping room thresholds for adequate quarantine conditions, respectively. Patterns of disparities by residential area and wealth quintiles remained unchanged.

## Discussion

Using the most recent data from the DHS and MICS in 56 LMICs since 2016, we provide the first assessment of the proportion of young children living in households with preparedness for communicable diseases, such as COVID-19, and associated residential and wealth disparities at both the country- and aggregate-level. We found that, on average, fewer than 20% of young children under the age of five lived in prepared households, with children in sub-Saharan African countries (4.6%) being the most disadvantaged. In addition, we observed significant residence- and/or wealth-disparities in most countries, favoring children living in urban areas or in the richest quintile. In 34 countries, fewer than 1% of children in the poorest quintile lived in prepared households. In many sub-Saharan or low-income countries, the small percentages of children living in prepared households in urban areas or in the richest quintile translated into relatively small residential or wealth disparities. The main conditions of poor household preparation were deficiencies in access to basic sanitation and hygiene facilities.

This study contributes to the body of literature on the extent to which current home-confining approaches to preventing the pandemic spread are likely to be effective in LMICs and underscores the barriers at home to prevent the spread of COVID-19 and other communicable diseases. The study reveals that for youngest children, especially in the poorest or rural areas, being confined to home through governmental lockdowns with the aim of protecting them and their families is in fact often not safe and potentially increases a range of other health and development risks during COVID-19 or other communicable disease crises. Though children have lower morbidity and better prognosis than older individuals in the case of COVID-19 [48], the long-term consequences of infection on children’s still developing physiology and neurobiology are as yet unknown, as are the effects of social isolation as well as potential increased poverty, parental mental distress, and child maltreatment. For those households without good preparation for COVID-19, policies that require household members, including COVID-19 patients with mild-moderate symptoms, to stay home may have increased the risk of infection for healthy young children and other household members. According to the Government Stringency Index [49], a composite index published by Oxford University to reflect the strictness of government containment policies preventing the spread of COVID-19 (e.g., school or business closure, restriction on movement, stay at home requirement), 50 of the 56 countries with available index measures implemented confinement policies since the onset of the pandemic, with the highest monthly averaged scores ranging from 23 in Burundi to 100 in the Philippines (scale value from 0 to 100, with 100 representing the strictest) [50]. Furthermore, some countries with low levels of household preparedness (e.g., Lesotho, 3.9% of young children living in prepared households) had high index scores (e.g., Lesotho, 91 scored in April 2020). This suggests that immediate actions are required to improve household conditions for preventing COVID-19 and avoiding containment measures having unintended adverse consequences for young children.

In many countries, efforts to contain the virus have been made in improving testing, tracing, and vaccination, and published studies have focused on assessing the risks of virus spread outside homes (e.g., bars, churches, workplaces) [51, 52]. However, policymakers and community leaders also need to work with households to address challenges of effective prevention and provision of safe care at home, including improving household water, sanitation, and hygiene conditions. Furthermore, confinement to households with a lack of space and crowding, especially with multiple children, minimizes the opportunities for play and stimulation as well as for more educational activities (such as reading) affecting development.

Poorer and rural households, which this study shows are less prepared for COVID-19 or other communicable diseases, are more likely to engage in informal work and are vulnerable to cessation and disruption by containment measures [53]; they are also more likely to be severely affected by COVID-19 due to comorbidities and poorer healthcare [53], have more COVID-19-related deaths [53, 54], and more likely to experience food shortages [53], interpersonal violence [55] and decreasing mental health [3]—all of which have important effects on young children. The largest urban–rural gap—and to a lesser extent between highest and lowest wealth quintiles—was mother’s weekly exposure to mass media. This is a critical finding when we consider that access to information has been playing an important role under COVID-19.

This study has the following limitations. First, our definition and measures of household preparedness for COVID-19 are based on data availability and do not fully capture the factors that affect household preparedness. For example, the availability of masks is not included due to a lack of household-level data. As more variables related to COVID-19 become available in household surveys, our definition and measures will improve. Second, data were only available for 56 LMICs, and the aggregate-level results are not representative at global, regional, or income levels. Third, we obtained data from different years, and caution is needed when making cross-country comparisons. In addition, for most countries, we used data collected before 2019, which may not necessarily capture the context in 2019 accurately, and thus underestimate the level of preparedness in countries with substantial progress immediately prior to 2019. However, the United Nations reports show that in sub-Saharan Africa, for example, the average coverage of basic sanitation only increased from 23% in 2000 to 30% in 2017 [56]. This suggests that for basic sanitation or hygiene facilities—the two main contributors to poor preparation for COVID-19—progress between 2016 and 2019 is unlikely to have been substantial. Further, we did a random-effects meta-analysis to compare the preparedness prevalence among countries with data collected before 2019 to that since 2019. The results revealed that, among countries with data collected before 2019, 19.1% (95% CI, 16.2%-22.1%) of children lived in prepared households. For countries with data collected since 2019, the prevalence is 20.1% (95% CI, 15.8%-24.4%), demonstrating slow preparedness progress. Fourth, except for basic hygiene (observed by interviewers), all variables used in our study are self-reported by households. While it has been documented that self-reported information could be subject to measurement errors, such as recall bias, we believe that variables used in this study (sleeping rooms, toilets, phones, mother’s weekly exposure to mass media) might be less likely to suffer from recall bias as they are not very time-sensitive. Fifth, as discussed earlier, the wealth-inequality gap in this study might have been smaller if we had excluded phones, toilets, and sleeping rooms from the wealth index. However, considering the large number of indicators included in constructing the wealth index in the 56 countries (for instance, between 70/83 and 135/162 indicators for the DHS and MICS countries, respectively), overestimation is probably not a major concern for this study. Nevertheless, future studies on this topic should explore constructing new wealth indices without the outcome variables and compare findings to the current ones.

Compared to populations in other age groups, young children have lower risks of infection and mortality from COVID-19. However, the next pandemic might not spare the young children as the COVID-19 does. Early childhood development provides a critical foundation for lifetime health, education, work productivity, and social wellbeing [57]. Aligned with previous studies in revealing the challenges faced by LIMCs households in preventing infections under confinement [58], our findings uniquely highlighted the dual threats imposed on young children by the pandemic disease itself and the household’s incapability to maintain a nurturing environment for children’s development amid a stringent lockdown policy. This study highlights the need to ensure all families have the means to prevent the spread of the pandemic or other communicable illnesses to young children when quarantined or isolated at home. The international community should launch cross-border collaborations to finance the improvement of household living conditions, such as water, sanitation, housing, information access, and communication technology, so as to protect young children’s health and wellbeing from adverse impacts of pandemics. In addition, the costs to development during stay-at-home orders and the health costs of nutritional and income loss are particularly devastating—and equally, urgently need to be mitigated [59]. During these challenging times, countries and global society need to realize the importance of ensuring the health and development of young children as the necessary basis for global development and prosperity in the coming years. Even with many competing priorities, reducing the negative impact of the COVID-19 pandemic on young children, including both disease risks and risks of lockdowns to healthy development, should be high on the agenda ‒ especially for children in rural areas, the poorest households, in sub-Saharan Africa, and in low-income countries. Meanwhile, to provide solid evidence for policymaking, further research efforts are needed to identify and track COVID-19 related risks (e.g., increased poverty and malnutrition, reduced stimulation) for young children; develop cost-effective interventions to minimize these risks; track related investments to improve efficiency.

## Conclusion

Less than 20% of young children under age five lived in prepared households, while Sub-Saharan African countries saw the lowest percentage of 4.6% on average. Deficiencies in access to basic hygiene and sanitation facilities constituted the main barriers to household preparedness. In addition, we observed significant disparities by residential areas and household wealth status in most countries, regions, and country income groups, with children in urban areas or the richest quintile being more likely to live in prepared households. Meanwhile, sub-Saharan African countries and low-income countries appeared to have relatively smaller residential/wealth disparities, primarily due to a poor preparedness for children living in urban or the richest quantile. This study highlighted the urgency of placing children’s prevention and development at the center of countries’ strategies combatting COVID-19 and other communicable diseases. To fundamentally empower households with adequate preparedness, more global initiatives and cross-border investments should be made to focus on building basic infrastructure and improving households’ living standards.

## Supplementary Information

**Additional file 1: Chapter 1**. Methods. **Chapter 2**. Results. **Table S1**. Keywords used for searching household surveys. **Table S2**. DHS and MICS data used in this study, 56 surveys. **Table S3**. Question examples used in DHS and MICS for the five groups of variables. **Table S4**. Sample size and percentage of missing values for each variable and country. **Table S5**. Analytical framework. **Table S6**. National prevalence (95% CI) of young children living in households with preparation for Covid-19 and associated disparity by place of residence in 56 countries using the most recent data since 2016. **Table S7**. National prevalence (95% CI) of young children living in households with preparation for Covid-19 and associated disparity by household wealth quintile in 56 countries using the most recent data since 2016. **Table S8**. National prevalence (95% CI) of young children living in households with adequate quarantine condition and associated disparity by place of residence in 56 countries using the most recent data since 2016 (<= three persons per room). **Table S9**. National prevalence (95% CI) of young children living in households with adequate quarantine condition and associated disparity by household wealth quintile in 56 countries using the most recent data since 2016 (<= three persons per room). **Table S10**. National prevalence (95% CI) of young children living in households with basic hygiene conditions and associated disparity by place of residence in 56 countries using the most recent data since 2016. **Table S11**. National prevalence (95% CI) of young children living in households with basic hygiene conditions and associated disparity by household wealth quintile in 56 countries using the most recent data since 2016. **Table S12**. National prevalence (95% CI) of young children living in households with basic sanitation conditions and associated disparity by place of residence in 56 countries using the most recent data since 2016. **Table S13**. National prevalence (95% CI) of young children living in households with basic sanitation conditions and associated disparity by household wealth quintile in 56 countries using the most recent data since 2016. **Table S14**. National prevalence (95% CI) of young children living in households with ownership of landline or mobile phones and associated disparity by place of residence in 56 countries using the most recent data since 2016. **Table S15**. National prevalence (95% CI) of young children living in households with ownership of landline or mobile phones and associated disparity by household wealth quintile in 56 countries using the most recent data since 2016. **Table S16**. National prevalence (95% CI) of young children with mother exposed to mass media at least once a week and associated disparity by place of residence in 56 countries using the most recent data since 2016. **Table S17**. National prevalence (95% CI) of young children with mother exposed to mass media at least once a week and associated disparity by household wealth quintile in 56 countries using the most recent data since 2016. **Table S18**. Aggregate-level prevalence (95% CI) of young children living in households with Covid-19 preparedness and adequate quarantine condition by region and income group. **Table S19**. Aggregate-level residential disparities in prevalence (95% CI) of young children living in households with Covid-19 preparedness and adequate quarantine condition by region and income group. **Table S20**. Aggregate-level wealth disparities in prevalence (95% CI) of young children living in households with Covid-19 preparedness and adequate quarantine condition by region and income group. **Table S21**. National prevalence (95% CI) of young children living in households with preparation for Covid-19 and associated disparity by place of residence in 56 countries using the most recent data since 2016 (adequate quarantine defined as <= two persons per room). **Table S22**. National prevalence (95% CI) of young children living in households with preparation for Covid-19 and associated disparity by household wealth quintile in 56 countries using the most recent data since 2016 (adequate quarantine defined as <= two persons per room). **Table S23**. National prevalence (95% CI) of young children living in households with preparation for Covid-19 and associated disparity by place of residence in 56 countries using the most recent data since 2016 (adequate quarantine defined as <= four persons per room). **Table S24**. National prevalence (95% CI) of young children living in households with preparation for Covid-19 and associated disparity by household wealth quintile in 56 countries using the most recent data since 2016 (adequate quarantine defined as <= four persons per room). **Table S25**. National prevalence (95% CI) of young children living in households with adequate quarantine condition and associated disparity by place of residence in 56 countries using the most recent data since 2016 (adequate quarantine defined as <= two persons per room). **Table S26**. National prevalence (95% CI) of young children living in households with adequate quarantine condition and associated disparity by household wealth quintile in 56 countries using the most recent data since 2016 (adequate quarantine defined as <= two persons per room). **Table S27**. National prevalence (95% CI) of young children living in households with adequate quarantine condition and associated disparity by place of residence in 56 countries using the most recent data since 2016 (adequate quarantine defined as <= four persons per room). **Table S28**. National prevalence (%, and 95% CI) of young children living in households with adequate quarantine condition and associated disparity by household wealth quintile in 56 countries using the most recent data since 2016 (adequate quarantine defined as <= four persons per room). **Figure S1**. National percentage (%) of young children living in households withpreparation for Covid-19 in 56 countries using available data in the most recent years since 2016 (adequate quarantine defined as <= two persons per room). **Figure S2**. National percentage (%) of young children living in households with preparation for Covid-19 in 56 countries using available data in the most recent years since 2016 (adequate quarantine defined as <= four persons per room). **Figure S3**. National percentage (%) of young children living in households with adequate quarantine condition in 56 countries using available data in the most recent years since 2016 (<= three persons per room). **Figure S4**. National percentage (%) of young children living in households with adequate quarantine condition in 56 countries using available data in the most recent years since 2016 (<= two persons per room). **Figure S5**. National percentage (%) of young children living in households with adequate quarantine condition in 56 countries using available data in the most recent years since 2016 (<= four persons per room). **Figure S6**. National percentage (%) of young children living in households with basic hygiene conditions in 56 countries using available data in the most recent years since 2016. **Figure S7**. National percentage (%) of young children living in households with basic sanitation conditions in 56 countries using available data in the most recent years since 2016. **Figure S8**. National percentage (%) of young children living in households with ownership of landline or mobile phones in 56 countries using available data in the most recent years since 2016. **Figure S9**. National percentage (%) of young children living in households with mother exposed to mass media at least once a week in 56 countries using available data in the most recent years since 2016.

## Data Availability

This study used data that are available from publicly accessible data sources. In particular, all MICS datasets are available from UNICEF’s online platform at https://mics.unicef.org/. All DHS datasets are available from the DHS online platform at https://dhsprogram.com/.

## Abbreviations

COVID-19: Coronavirus diseases 2019
LMICs: Low- and middle-income countries
WHO: World Health Organization
CDC: Center for disease control
IHSN: International household survey network
DHS: Demographic and health surveys
MICS: Multiple indicator cluster surveys
CI: Confidence interval
SSA: Sub-Saharan African
LIC: Low-income country
