# Correction to: Assessing the prevalence of young children living in households prepared for COVID-19 in 56 low- and middle-income countries

**DOI:** 10.1186/s41256-022-00256-0

**Published:** 2022-07-19

**Authors:** Chunling Lu, Yiqun Luan, Sara N. Naicker, S. V. Subramanian, Jere R. Behrman, Jody Heymann, Alan Stein, Linda M. Richter

**Affiliations:** 1 Division of Global Health Equity, Brigham and Women’s Hospital, Boston, MA USA; 2 Department of Global Health and Social Medicine, Harvard Medical School, Boston, MA USA; 3 The Heller School for Social Policy and Management, Brandeis University, Waltham, MA USA; 4 DSI-NRF Centre of Excellence in Human Development, University of Witwatersrand, Johannesburg, South Africa; 5 Harvard Center for Population and Development Studies, Cambridge, MA USA; 6 Department of Social and Behavioral Sciences, Harvard T.H. Chan School of Public Health, Boston, MA USA; 7 Center for Geographic Analysis, Harvard University, Cambridge, MA USA; 8 Department of Economics, University of Pennsylvania, Philadelphia, PA USA; 9 WORLD Policy Analysis Center, University of California, Los Angeles, Los Angeles, CA USA; 10 Department of Psychiatry, University of Oxford, Oxford, UK; 11 MRC/Wits Rural Public Health and Health Transitions Research Unit (Agincourt), Faculty of Health Sciences, School of Public Health, University of the Witwatersrand, Johannesburg, South Africa; 12 Africa Health Research Institute, Durban, KwaZulu-Natal South Africa

## Correction to: Global Health Research and Policy (2022) 7:18 10.1186/s41256-022-00254-2

Following publication of the original article [1], the authors reported that one reference number needs to be corrected, and the captions listed in the Supplementary Information section need to be updated.

The original article [1] has been corrected.
