# Supplementary material for: Assessing the prevalence of young children living in households prepared for COVID-19 in 56 low- and middle-income countries
Source: Glob Health Res Policy. 2022 Jun 21;7:18. doi: 10.1186/s41256-022-00254-2 (PMC9210057; doi:10.1186/s41256-022-00254-2)
Supplement: Supplementary file 1 — Additional file 1: Chapter 1. Methods. Chapter 2. Results. Table S1. Keywords used for searching household surveys. Table S2. DHS and MICS data used in this study, 56 surveys. Table S3. Question examples used in DHS and MICS for the five groups of variables. Table S4. Sample size and percentage of missing values for each variable and country. Table S5. Analytical framework. Table S6. National prevalence (95% CI) of young children living in households with preparation for Covid-19 and associated disparity by place of residence in 56 countries using the most recent data since 2016. Table S7. National prevalence (95% CI) of young children living in households with preparation for Covid-19 and associated disparity by household wealth quintile in 56 countries using the most recent data since 2016. Table S8. National prevalence (95% CI) of young children living in households with adequate quarantine condition and associated disparity by place of residence in 56 countries using the most recent data since 2016 (<= three persons per room). Table S9. National prevalence (95% CI) of young children living in households with adequate quarantine condition and associated disparity by household wealth quintile in 56 countries using the most recent data since 2016 (<= three persons per room). Table S10. National prevalence (95% CI) of young children living in households with basic hygiene conditions and associated disparity by place of residence in 56 countries using the most recent data since 2016. Table S11. National prevalence (95% CI) of young children living in households with basic hygiene conditions and associated disparity by household wealth quintile in 56 countries using the most recent data since 2016. Table S12. National prevalence (95% CI) of young children living in households with basic sanitation conditions and associated disparity by place of residence in 56 countries using the most recent data since 2016. Table S13. National prevalence (95% CI) of young children li [file 41256_2022_254_MOESM1_ESM.docx]

**Additional file 1**

**Chapter 1 Methods**

1. **Household survey search**

To gather household surveys with available variables indicating the five conditions as listed in Table S1, we searched the micro-level surveys through the International Household Survey Network (IHSN), a website in collaboration with international (e.g., World Bank, WHO) and national agencies (e.g., USAID).^1^ The mission of the IHSN is to improve the availability, quality, and accessibility of survey data in LMICs. The catalog provides a searchable list of household surveys (including data, questionnaires, and reports for some of them) conducted in LMICs with different data types, such as household income and expenditure, population health and nutrition, census, and living conditions. We searched through the surveys that were nationally representative and publicly accessible since 2016. A keyword strategy was used to identify the surveys with variables related to the five conditions (**Table S1**). We found some household surveys with variables related to one or two conditions of preparedness, but only Demographic and Health Surveys (DHS)^2^ and Multiple Indicator Cluster Surveys (MICS)^3^ have variables covering all five conditions. The two surveys are highly comparable due to their similar sampling, implementation, and measurement strategies.^4^ For example, both surveys used a two-stage sampling design, with randomly selecting enumeration areas from the most recent population census as the primary sample unit in the first stage, followed by randomly selecting households or persons to be interviewed in each primary sample unit in the second stage.

**References**

International Household Surveys Network. Central survey Catalog. <http://www.ihsn.org/survey-catalogs> (accessed 16 April 2021).

Demographic and Health Surveys. <https://dhsprogram.com/data> (accessed 3 May 2021)

Multiple Indicator Cluster Surveys. <https://mics.unicef.org> (accessed 3 May 2021)

World Health Organization. Equity country profiles.

Available: https://www.who.int/docs/default-source/gho-documents/health-equity/state-of-inequality/technical-notes/health-equity-country-profiles.pdf?sfvrsn=9c3f023_2

**Table S1.** Keywords used for searching household surveys

| 1. Adequate quarantine: household size, member of household, room  2. Basic hygiene: handwash, handwashing, hand wash, wash hand, soap, detergent  3. Basic sanitation: toilet, flush, defecation  4. Ownership of phones: phone  5. Media exposure: TV, television, radio, newspaper, magazine, mass media |
| --- |

**Table S2.** DHS and MICS data used in this study, 56 surveys

| **Country** | **Year** | **Survey** | **Country income group** | **Region** |
| --- | --- | --- | --- | --- |
| Algeria | 2019 | MICS | Upper-middle | Middle East and North Africa |
| Angola | 2016 | DHS | Upper-middle | Sub-Saharan Africa |
| Armenia | 2016 | DHS | Lower-middle | Europe and Central Asia |
| Bangladesh | 2019 | MICS | Lower-middle | South Asia |
| Benin | 2018 | DHS | Low-income | Sub-Saharan Africa |
| Burundi | 2017 | DHS | Low-income | Sub-Saharan Africa |
| Cameroon | 2018 | DHS | Lower-middle | Sub-Saharan Africa |
| Central African Republic | 2018 | MICS | Low-income | Sub-Saharan Africa |
| Chad | 2019 | MICS | Low-income | Sub-Saharan Africa |
| Congo DR | 2018 | MICS | Low-income | Sub-Saharan Africa |
| Cote d’Ivoire | 2016 | MICS | Lower-middle | Sub-Saharan Africa |
| Dominican Republic | 2019 | MICS | Upper-middle | Latin America and the Caribbean |
| Ethiopia | 2016 | DHS | Low-income | Sub-Saharan Africa |
| Gambia | 2020 | DHS | Low-income | Sub-Saharan Africa |
| Ghana | 2017 | MICS | Lower-middle | Sub-Saharan Africa |
| Guinea | 2018 | DHS | Low-income | Sub-Saharan Africa |
| Guinea-Bissau | 2019 | MICS | Low-income | Sub-Saharan Africa |
| Guyana | 2019 | MICS | Upper-middle | Latin America and the Caribbean |
| Haiti | 2017 | DHS | Low-income | Latin America and the Caribbean |
| India | 2016 | DHS | Lower-middle | South Asia |
| Indonesia | 2017 | DHS | Lower-middle | East Asia and Pacific |
| Iraq | 2018 | MICS | Upper-middle | Middle East and North Africa |
| Kiribati | 2018 | MICS | Lower-middle | East Asia and Pacific |
| Kyrgyzstan | 2018 | MICS | Lower-middle | Europe and Central Asia |
| Lao | 2017 | MICS | Lower-middle | East Asia and Pacific |
| Lesotho | 2018 | MICS | Lower-middle | Sub-Saharan Africa |
| Liberia | 2019 | DHS | Low-income | Sub-Saharan Africa |
| Madagascar | 2018 | MICS | Low-income | Sub-Saharan Africa |
| Malawi | 2020 | MICS | Low-income | Sub-Saharan Africa |
| Maldives | 2017 | DHS | Upper-middle | South Asia |
| Mali | 2018 | DHS | Low-income | Sub-Saharan Africa |
| Mongolia | 2018 | MICS | Lower-middle | East Asia and Pacific |
| Myanmar | 2016 | DHS | Lower-middle | East Asia and Pacific |
| Nepal | 2019 | MICS | Low-income | South Asia |
| Nigeria | 2018 | DHS | Lower-middle | Sub-Saharan Africa |
| Pakistan | 2018 | DHS | Lower-middle | South Asia |
| Palestine | 2019 | MICS | Lower-middle | Middle East and North Africa |
| Papua New Guinea | 2016-18 | DHS | Lower-middle | East Asia and Pacific |
| Paraguay | 2016 | MICS | Upper-middle | Latin America and the Caribbean |
| Philippines | 2017 | DHS | Lower-middle | East Asia and Pacific |
| Rwanda | 2020 | DHS | Low-income | Sub-Saharan Africa |
| Samoa | 2019 | MICS | Lower-middle | East Asia and Pacific |
| São Tomé and Príncipe | 2019 | MICS | Lower-middle | Sub-Saharan Africa |
| Senegal | 2019 | DHS | Low-income | Sub-Saharan Africa |
| Sierra Leone | 2019 | DHS | Low-income | Sub-Saharan Africa |
| South Africa | 2016 | DHS | Upper-middle | Sub-Saharan Africa |
| Suriname | 2018 | MICS | Upper-middle | Latin America and the Caribbean |
| Tajikistan | 2017 | DHS | Lower-middle | Europe and Central Asia |
| Timor-Leste | 2016 | DHS | Lower-middle | East Asia and Pacific |
| Togo | 2017 | MICS | Low-income | Sub-Saharan Africa |
| Tonga | 2019 | MICS | Lower-middle | East Asia and Pacific |
| Tunisia | 2018 | MICS | Lower-middle | Middle East and North Africa |
| Tuvalu | 2019 | MICS | Upper-middle | East Asia and Pacific |
| Uganda | 2016 | DHS | Low-income | Sub-Saharan Africa |
| Zambia | 2018 | DHS | Lower-middle | Sub-Saharan Africa |
| Zimbabwe | 2019 | MICS | Low-income | Sub-Saharan Africa |

1. **Constructing measures for the five conditions on preparedness**
2. Young children living in households with adequate quarantine conditions

We used two variables to construct a binary variable indicating household quarantine conditions: number of household members who usually live in the household, and number of rooms used for sleeping in a household. We first obtained the ratio of these two variables which indicates the number of persons per sleeping room. We then defined a household with adequate quarantine conditions based on the WHO and United Nations criteria on household crowding, which is three persons or less per sleeping room.^1,2^ As overcrowding definitions could vary across different cultural contexts, we tested the sensitivity of analysis using two cutoff values for a lower bound (two persons per sleeping room) and an upper bound (four persons per sleeping room). We then merged this household-level binary variable on quarantine conditions into the dataset for young children to obtain information on whether a child lived in a household with adequate quarantine conditions.

1. Young children living in households with basic hygiene facilities

Performing hand hygiene is considered one of the most important measures in preventing COVID-19, requiring household members to wash hands regularly with water and soap.^3^ According to WHO and UNICEF,^4^ a household meets the criteria for having basic hygiene facilities if it has a handwashing facility with water and soap available. The DHS and MICS include questions on hand hygiene. Though the survey questions varied across countries or years (see examples in **Table S3)**, they all include three core components: (a) if a fixed or mobile handwashing facility was observed in a dwelling or yard by the surveyor, (b) if water for handwashing was observed, and (c) if any cleansing agent was observed. With these variables, we constructed a binary variable, with a value of 1 indicating a household having a handwashing facility with water and soap and 0 otherwise. If a household only had cleansing agents such as ash, mud, or sand, it was coded as 0. A small proportion of households not observed were excluded from the analysis. We merged the household-level binary variable into the child data.

1. Young children living in households with basic sanitation facilities

##### As recommended by the WHO, to minimize the risk of infection when caring for a patient with suspected or confirmed COVID-19, direct contact with the patient’s stool, urine and other waste must be avoided.^5^ Both DHS and MICS have a group of variables indicating the type of sanitation facility in a household. We followed the guidance of the WHO/UNICEF Joint Management Programme (JMP) for Water Supply, Sanitation and Hygiene^4^ and constructed a binary variable, with a value of 1 indicating a young child living in a household using sanitation not shared with other households and with hygienic separation of excreta from human contact (e.g., flush to piped sewer system/septic tank/pit latrine, ventilated improved pit, pit latrine with slab, and composting toilet), and 0 otherwise.

1. Young children living in households that stay connected

Households having the means to call for medical assistance and staying in touch with outside support or resources is recommended by WHO for assessing household readiness for home prevention and care.^5^ We measured this condition using household ownership of at least one phone (landline or mobile) in the DHS and MICS. A binary variable was constructed to indicate whether a household owned a phone (1 yes, and 0 otherwise). This binary variable was then merged into the child dataset.

1. Young children living in households that stay informed about COVID-19

When assessing household suitability for home prevention or care, a key factor is a household’s capacity to obtain up-to-date information about COVID-19.^5^ Mass media has been found to be efficient in conveying messages on public health topics.^6^ DHS and MICS include a group of variables on mass media exposure among caregivers of young children. Respondents (aged 15-49) were asked how often they read a newspaper, listened to the radio, or watched TV. Those who answered at least once a week are considered to be exposed to that specific media regularly^7^. To measure children living in households that stay informed about COVID-19, we followed the DHS and MICS practice and constructed a binary variable indicating if a child’s caregivers were exposed to any one of the three types of media at least once a week (1 yes, and 0 otherwise).

**References**

1. WHO. WHO Housing and Health Guidelines. 2018. <https://www.ncbi.nlm.nih.gov/books/NBK535293/pdf/Bookshelf_NBK535293.pdf> (accessed 3 May 2021).
2. United Nations. Principles and Recommendations for Population and Housing Censuses. Revision 2. 2007. <https://unstats.un.org/unsd/demographic-social/Standards-and-Methods/files/Principles_and_Recommendations/Population-and-Housing-Censuses/Series_M67Rev2-E.pdf>.
3. WHO. Coronavirus disease (COVID-19): Home care for families and caregivers. . 2020. <https://www.who.int/news-room/q-a-detail/coronavirus-disease-covid-19-home-care-for-families-and-caregivers> (accessed 3 May 2021).
4. WHO/UNICEF Joint Monitoring Programme. Hygiene. 2020. <https://washdata.org/monitoring/hygiene> (accessed 3 May 2021).
5. WHO. Home care for patients with suspected or confirmed COVID-19 and management of their contacts, interim guidance. 2020. <https://apps.who.int/iris/rest/bitstreams/1292529/retrieve> (accessed December 2020).
6. Anwar A, Malik M, Raees V, Anwar A. Role of mass media and public health communications in the COVID-19 pandemic. *Cureus* 2020; **12**(9): e104543.
7. International Institute for Population Sciences (IIPS) and ICF. 2017. National Family Health Survey (NFHS-4), 2015-16: India. Mumbai: IIPS.
8. **Measuring country-level socioeconomic inequalities using logistic regression**

For each country, we used logistic regression models to test for the inequalities in young children living in households with preparedness for the COVID-19 by wealth (highest vs. lowest wealth quintile) or residential areas (urban vs. rural). The dependent variable is a dichotomous variable indicating a young child living in a prepared household, and the exposure variable is a dichotomous variable indicating either wealth status or residential area. The equation is shown below when taking residential area as an example:

$$Logit\left( {living in prepared household}_{i} \right)=\beta_{0}+\beta_{1}{Urban}_{i}+e_{i}$$

Where $Logit\left( {living in prepared household}_{i} \right)$ indicates the logit of the probability of living in a prepared household for the $i^{th}$ child in the country, ${Urban}_{i}$ indicates if the $i^{th}$ child lived in an urban area, and $e_{i}$ indicates the error term. $\beta_{0}$ is a constant. $\beta_{1}$ represents the logarithm of the odds ratio of living in a prepared household for an average child living in urban area compared to an average child living in rural area. Using STATA codes, we were able to obtain the difference (and its statistical significance) of the probabilities of living in prepared households between the urban and rural young children. See the disparity results derived from the logistic regressions in the last columns of Table S6 and Table S7.

1. **Generating aggregate-level estimates using random-effects meta-analysis**

We followed previous studies^1,2^ and used meta-analysis to estimate the aggregate-level estimates. We first generated the country-level estimates and then combined these estimates into a weighted average using random-effects meta-analysis with the DerSimonian and Laird method (DL). We chose the DL method because it is the most commonly used approach in meta-analyses which assumes heterogeneity of the effects (or quantitative measures of outcome variables) observed in different studies and that the collected studies (in our case, the included countries) represent a random sample from a large population of studies.^3^ For our study, the heterogeneity assumption refers to not assuming a common homogeneous estimate of the prevalence of children living in prepared households across the 56 countries. The same assumption also applies to the disparities by residential area and wealth. We conducted three Cochran’s Q homogeneity tests to examine our assumptions. Each of the three tests reports a p-value of zero, indicating the rejection of homogeneity across the 56 countries. To process the random-effects meta-analysis, we used STATA code “meta set” to declare the country-level estimates as the main information about the analysis and specify their corresponding standard errors and the DL estimate method. Then we used codes “meta summarize” with the “subgroup” as an option to request the aggregate-level estimates based on countries’ income and region categories.

**References**

1. McKinnon, B., Harper, S., Kaufman, J. S., & Bergevin, Y. (2014). Socioeconomic inequality in neonatal mortality in countries of low and middle income: a multicountry analysis. The Lancet Global Health, 2(3), e165-e173.
2. Yang, F., Li, Z., Subramianian, S. V., & Lu, C. (2021). Assessment of Knowledge of HIV/AIDS and Association With Socioeconomic Disparities Among Young Women in Low-and Middle-Income Countries, 2003 to 2018. JAMA network open, 4(1), e2035000-e2035000.
3. Kontopantelis, E., & Reeves, D. (2010). metaan: Random-effects meta-analysis. The Stata Journal, 10(3), 395-407.

**Table S3.** Question examples used in DHS and MICS for the five groups of variables

| **Variable** | **Survey** | **Related questions** |
| --- | --- | --- |
| Adequate quarantine | DHS | - Household size: please give me the names of the persons who usually live in your household and guests of the household who stayed here last night, starting with the head of the household. - Number of sleeping rooms: how many rooms in this household are used for sleeping? |
|  | MICS | - Household size: first, please tell me the name of each person who usually lives here, starting with the head of the household. - Number of sleeping rooms: how many rooms do members of this household usually use for sleeping? |
| Basic hygiene facility | DHS | - Please show me where members of your household most often wash their hands - Observed - Not observed, not in dwelling/yard/plot - Not observed, no permission to see - Not observed, other reason - Observe presence of water at the place for handwashing - Observe presence of soap, detergent, or other cleansing agent |
|  | MICS | - Observed handwashing facility: we would like to learn about where members of this household wash their hands. - Observed: - Fixed facility observed (sink/tap/tube well) - Mobile object observed - Not observed: - No handwashing place in dwelling/yard/plot - No permission to see - Other reason (specify) - Observe presence of water at the place for handwashing. - Is soap or detergent or ash/mud/sand present at the place for handwashing? - Where do you or other members of your household most often wash your hands? - Fixed facility (sink/tap/tube well) - Mobile object - No handwashing place in dwelling/yard/plot - Other (specify) - Do you have any soap or detergent or ash/mud/sand in your house for washing hands? - Can you please show it (soap or detergent or ash/mud/sand) to me? |
| Basic sanitation | DHS | - What kind of toilet facility do members of your household usually use? - Flush or pour flush toilet - Flush to piped sewer system - Flush to septic tank - Flush to pit latrine - Flush to somewhere else - Flush, don’t know where - Pit latrine - Ventilated improved pit latrine - Pit latrine with slab - Pit latrine without slab/open pit - Composting toilet - Bucket toilet - Hanging toilet/handing latrine - No facility/bush/field - Other (specify) |
|  | MICS | - What kind of toilet facility do members of your household usually use? - Flush/pour flush - Flush to piped sewer system - Flush to septic tank - Flush to pit latrine - Flush to open drain - Flush to don’t know where - Pit latrine - Ventilated improved pit latrine - Pit latrine with slab - Pit latrine without slab/open pit - Composting toilet - Bucket - Hanging toilet/hanging latrine - No facility/bush field - Other (specify) |
| Ownership of phones | DHS | - Does your household have: - A mobile telephone? - A landline telephone? |
|  | MICS | - Does any member of your household have a mobile telephone? - Does your household have a fixed telephone line? |
| Media exposure | DHS | - Do you read a newspaper or magazine at least once a week, less than once a week or not at all^*^? - Do you listen to the radio at least once a week, less than once a week or not at all^*^? - Do you watch television at least once a week, less than once a week or not at all^*^? - At least once a week - Less than once a week - Not at all |
|  | MICS | - Do you read a newspaper or magazine at least once a week, less than once a week or not at all? - Do you listen to the radio at least once a week, less than once a week or not at all? - Do you watch television at least once a week, less than once a week or not at all? - Not at all - Less than once a week - At least once a week - Almost every day |

* DHS does not have an “Almost every day” category for respondents’ media exposure

**Table S4.** Sample size and percentage of missing values for each variable and country

| **Country** | **Year** | **Survey** | **Sample size (household/woman)^[[1]](#footnote-2)^** | **Adequate**  **quarantine^[[2]](#footnote-3)^** | **Basic hygiene facility^2^** | **Basic**  **Sanitation^2^** | **Ownership**  **of phones^2^** | **Media**  **exposure^[[3]](#footnote-4)^** |
| --- | --- | --- | --- | --- | --- | --- | --- | --- |
| Algeria | 2019 | MICS | 29,919/35,111 | 0.00% | 0.28% | 0.17% | 0.05% | 0.00% |
| Angola | 2016 | DHS | 16,109/14,379 | 0.00% | 7.28% | 0.00% | 0.00% | 0.00% |
| Armenia | 2016 | DHS | 7,893/6,116 | 0.18% | 3.28% | 0.05% | 0.00% | 0.00% |
| Bangladesh | 2019 | MICS | 61,242/64,378 | 0.00% | 0.17% | 0.03% | 0.00% | 0.00% |
| Benin | 2018 | DHS | 14,156/15,928 | 0.00% | 2.00% | 0.00% | 0.00% | 0.00% |
| Burundi | 2017 | DHS | 15,977/17,269 | 0.00% | 0.22% | 0.00% | 0.00% | 0.00% |
| Cameroon | 2018 | DHS | 11,710/14,677 | 0.00% | 1.18% | 0.00% | 0.00% | 0.00% |
| Central African Republic | 2018 | MICS | 8,133/9,202 | 0.00% | 0.50% | 0.15% | 0.38% | 0.00% |
| Chad | 2019 | MICS | 18,967/22,561 | 0.00% | 0.77% | 0.08% | 0.23% | 0.02% |
| Congo DR | 2018 | MICS | 20,792/21,756 | 0.00% | 0.31% | 0.09% | 0.00% | 0.34% |
| Cote d’Ivoire | 2016 | MICS | 11,879/11,780 | 0.00% | 5.19% | 0.04% | 0.01% | 0.00% |
| Dominican Republic | 2019 | MICS | 31,488/22,295 | 0.00% | 0.64% | 0.27% | 0.04% | 0.00% |
| Ethiopia | 2016 | DHS | 16,650/15,683 | 0.25% | 2.50% | 0.00% | 0.00% | 0.00% |
| Gambia | 2020 | DHS | 6,549/11,865 | 0.00% | 2.98% | 0.00% | 0.00% | 0.00% |
| Ghana | 2017 | MICS | 12,886/14,374 | 0.00% | 0.17% | 0.02% | 0.01% | 0.00% |
| Guinea | 2018 | DHS | 7,912/10,874 | 0.00% | 3.65% | 0.00% | 0.00% | 0.00% |
| Guinea-Bissau | 2019 | MICS | 7,379/10,945 | 0.00% | 0.01% | 0.00% | 0.00% | 0.00% |
| Guyana | 2019 | MICS | 7,072/5,887 | 0.00% | 0.99% | 0.18% | 0.06% | 0.00% |
| Haiti | 2017 | DHS | 13,405/15,513 | 0.00% | 2.45% | 0.00% | 0.00% | 0.00% |
| India | 2016 | DHS | 601,509/699,686 | 0.74% | 0.64% | 0.00% | 0.00% | 0.00% |
| Indonesia | 2017 | DHS | 47,963/49,627 | 0.72% | 1.15% | 0.02% | 0.04% | 0.01% |
| Iraq | 2018 | MICS | 20,214/30,660 | 0.00% | 0.07% | 0.02% | 0.02% | 0.00% |
| Kiribati | 2018 | MICS | 3,071/4,150 | 0.00% | 1.04% | 0.07% | 0.00% | 0.00% |
| Kyrgyzstan | 2018 | MICS | 6,968/5,742 | 0.00% | 0.01% | 0.00% | 1.45% | 0.00% |
| Lao | 2017 | MICS | 22,287/25,305 | 0.00% | 0.02% | 0.03% | 0.02% | 0.00% |
| Lesotho | 2018 | MICS | 8,847/6,453 | 0.00% | 0.76% | 0.06% | 0.05% | 0.00% |
| Liberia | 2019 | DHS | 9,068/8,065 | 0.00% | 24.82% | 0.00% | 0.00% | 0.00% |
| Madagascar | 2018 | MICS | 17,870/ 17,161 | 0.00% | 0.84% | 0.02% | 0.00% | 0.01% |
| Malawi | 2020 | MICS | 25,419/24,543 | 0.00% | 0.72% | 0.11% | 0.21% | 0.00% |
| Maldives | 2017 | DHS | 6,050/7,699 | 0.00% | 0.99% | 0.00% | 0.00% | 0.00% |
| Mali | 2018 | DHS | 9,510/ 10,519 | 0.00% | 6.53% | 0.00% | 0.00% | 0.00% |
| Mongolia | 2018 | MICS | 13,798/10,794 | 0.00% | 0.70% | 0.13% | 0.01% | 0.00% |
| Myanmar | 2016 | DHS | 12,500/12,885 | 2.25% | 1.50% | 0.00% | 0.00% | 0.00% |
| Nepal | 2019 | MICS | 12,655/14,805 | 0.00% | 0.21% | 0.09% | 0.00% | 0.00% |
| Nigeria | 2018 | DHS | 40,427/41,821 | 0.00% | 8.72% | 0.00% | 0.00% | 0.00% |
| Pakistan | 2018 | DHS | 14,540/15,068 | 0.23% | 5.39% | 0.01% | 0.03% | 0.00% |
| Palestine | 2019 | MICS | 9,326/ 11,135 | 0.14% | 0.57% | 0.35% | 0.00% | 0.02% |
| Papua New Guinea | 2016-18 | DHS | 16,021/15,198 | 4.15% | 13.46% | 0.54% | 0.31% | 0.09% |
| Paraguay | 2016 | MICS | 7,313/7,311 | 0.00% | 8.04% | 0.29% | 0.00% | 0.00% |
| Philippines | 2017 | DHS | 27,496/25,074 | 0.00% | 2.93% | 0.00% | 0.00% | 0.00% |
| Rwanda | 2020 | DHS | 12,949/14,634 | 0.00% | 0.65% | 0.00% | 0.00% | 0.00% |
| Samoa | 2019 | MICS | 3,196/4,139 | 0.00% | 0.31% | 0.16% | 0.19% | 0.00% |
| São Tomé and Príncipe | 2019 | MICS | 3,426/3,115 | 0.00% | 0.76% | 0.67% | 0.00% | 0.00% |
| Senegal | 2019 | DHS | 4,538/8,649 | 0.00% | 5.31% | 0.00% | 0.00% | 0.00% |
| Sierra Leone | 2019 | DHS | 13,399/15,574 | 0.00% | 12.08% | 0.00% | 0.00% | 0.00% |
| South Africa | 2016 | DHS | 11,083/8,514 | 0.00% | 6.44% | 0.00% | 0.00% | 0.00% |
| Suriname | 2018 | MICS | 7,915/6,999 | 0.00% | 1.30% | 0.19% | 0.11% | 0.01% |
| Tajikistan | 2017 | DHS | 7,843/10,718 | 0.00% | 1.21% | 0.00% | 0.00% | 0.00% |
| Timor-Leste | 2016 | DHS | 11,502/12,607 | 0.00% | 6.05% | 0.00% | 0.00% | 0.00% |
| Togo | 2017 | MICS | 7,916/7,326 | 0.00% | 0.04% | 0.00% | 0.00% | 0.00% |
| Tonga | 2019 | MICS | 2,498/2,903 | 0.00% | 0.68% | 0.04% | 0.04% | 0.00% |
| Tunisia | 2018 | MICS | 11,225/10,559 | 0.00% | 0.17% | 0.04% | 0.01% | 0.00% |
| Tuvalu | 2019 | MICS | 695/817 | 0.00% | 0.58% | 0.00% | 0.00% | 0.00% |
| Uganda | 2016 | DHS | 19,588/18,506 | 0.00% | 15.07% | 0.00% | 0.00% | 0.00% |
| Zambia | 2018 | DHS | 12,831/13,683 | 0.00% | 14.93% | 0.00% | 0.00% | 0.00% |
| Zimbabwe | 2019 | MICS | 11,091/10,129 | 0.00% | 0.41% | 0.00% | 0.04% | 0.00% |

In five countries (Liberia, Papua New Guinea, Sierra Leone, Uganda, and Zambia), the percentage of missing data in basic hygiene is larger than 10%. We followed previous practice and the recommendations by the DHS and MICS programs^1-3^ and excluded the missing data from the statistical calculations. We acknowledge that excluding the missing data from estimation could have a potential impact on estimation if the % of missingness is large and data missing is systematic (not random). Our investigation into the missing data in the five countries found that, except in Papua New Guinea and Uganda, the missing data are not highly concentrated in some groups with specific socioeconomic characteristics. In Papua New Guinea and Uganda, most missing data were reported by rural households. The impact of this concentration on estimates in these two countries is unknown as the households with missing data were excluded from both the numerator and the denominator when calculating the prevalence.

**References**

1. Demographic and Health Surveys Program, Data quality and use. <https://dhsprogram.com/data/Data-Quality-and-Use.cfm> (accessed 3 May 2021)
2. Bangladesh Bureau of Statistics & UNICEF Bangladesh (2019) Progotir Pathey, Bangladesh Multiple Indicator Cluster Survey 2019, Survey Findings Report. (Bangladesh Bureau of Statistics, Dhaka, Bangladesh). <https://mics.unicef.org/surveys>.
3. McLaren SW. The Relationship between Hemoglobin Level and Socio-economic Indicators among Women of Childbearing Age in South Africa: A Secondary Analysis of DHS Data. Ecology of Food and Nutrition. 2021 Jul 16:1-8.

**Table S5.** Analytical framework

|  | **Preparedness** | **Adequate quarantine** | **Basic hygiene facility** | **Basic sanitation** | **Ownership of phones** | **Media exposure** |
| --- | --- | --- | --- | --- | --- | --- |
| **(1) Estimating prevalence (%) of children** | | | | | | |
| a. Country-level | X | X | X | X | X | X |
| b. Aggregate-level |  |  |  |  |  |  |
| - by income group | X | X | X | X | X | X |
| - by region | X | X | X | X | X | X |
| **(2) Assessing inequalities** | | | | | | |
| a. Country-level |  |  |  |  |  |  |
| - by residential area | X | X | X | X | X | X |
| - by wealth quintile | X | X | X | X | X | X |
| b. Aggregate-level |  |  |  |  |  |  |
| - by residential area | X | X | X | X | X | X |
| - by wealth quintile | X | X | X | X | X | X |

**Chapter 2 Results**

*(1) Country-level estimates with adequate quarantine condition defined as <= three persons per room*

**Table S6.** National prevalence of young children living in households with preparedness and associated disparities by place of residence

| Country | Year | WB region^1^ | Income group^2^ | National (%) | Rural (%) | Urban (%) | Difference  (urban-rural) |
| --- | --- | --- | --- | --- | --- | --- | --- |
| Algeria | 2019 | MENA | UM | 45.0(43.0, 47.0) | 36.8(33.3, 40.2) | 51.1(48.9, 53.2) | 14.3(10.2, 18.4) |
| Angola | 2016 | SSA | UM | 8.1(6.5, 9.6) | 0.9(0.2, 1.5) | 12.9(10.4, 15.4) | 12.0(9.4, 14.6) |
| Armenia | 2016 | ECA | LM | 63.3(59.7, 66.9) | 43.8(38.8, 48.9) | 78.0(73.6, 82.5) | 34.2(27.4, 40.9) |
| Bangladesh | 2019 | SA | LM | 28.0(27.1, 28.9) | 24.7(23.8, 25.7) | 40.3(37.8, 42.7) | 15.5(12.9, 18.2) |
| Benin | 2018 | SSA | L | 1.4(1.0, 1.7) | 0.4(0.2, 0.6) | 3.0(2.1, 3.8) | 2.6(1.7, 3.5) |
| Burundi | 2017 | SSA | L | 1.3(0.9, 1.7) | 0.7(0.4, 1.0) | 7.5(4.7, 10.4) | 6.9(4.0, 9.7) |
| CAR | 2018 | SSA | L | 0.8(0.6, 1.1) | 0.1(-0.1, 0.3) | 2.6(1.8, 3.3) | 2.4(1.6, 3.2) |
| Cameroon | 2018 | SSA | LM | 8.9(7.7, 10.1) | 2.4(1.3, 3.4) | 16.9(14.7, 19.2) | 14.6(12.1, 17.1) |
| Chad | 2019 | SSA | L | 1.7(1.4, 2.1) | 0.3(0.2, 0.5) | 9.3(7.4, 11.3) | 9.0(7.1, 11.0) |
| Congo DR | 2018 | SSA | L | 1.5(1.1, 2.0) | 0.0(0.0, 0.0) | 3.9(2.7, 5.1) | 3.9(2.7, 5.0) |
| Cote d’Ivoire | 2016 | SSA | LM | 4.7(3.6, 5.8) | 0.9(0.6, 1.3) | 10.7(8.0, 13.4) | 9.8(7.1, 12.5) |
| Dominican Republic | 2019 | LAC | UM | 34.6(32.6, 36.6) | 23.6(20.8, 26.4) | 38.6(36.1, 41.1) | 15.0(11.3, 18.8) |
| Ethiopia | 2016 | SSA | L | 0.6(0.3, 0.9) | 0.0(0.0, 0.0) | 5.4(2.7, 8.0) | 5.4(2.7, 8.1) |
| Gambia | 2020 | SSA | L | 3.8(2.8, 4.8) | 1.3(0.7, 1.9) | 5.1(3.6, 6.6) | 3.8(2.2, 5.4) |
| Ghana | 2017 | SSA | LM | 6.7(5.5, 7.9) | 3.6(2.3, 4.9) | 10.8(8.4, 13.2) | 7.2(4.5, 9.9) |
| Guinea | 2018 | SSA | L | 2.7(2.1, 3.4) | 0.7(0.3, 1.1) | 7.9(6.0, 9.9) | 7.2(5.2, 9.2) |
| Guinea-Bissau | 2019 | SSA | L | 1.4(1.0, 1.9) | 0.4(0.1, 0.7) | 4.4(2.9, 5.9) | 4.0(2.5, 5.5) |
| Guyana* | 2019 | LAC | UM | 47.2(42.7, 51.7) | 47.1(41.7, 52.5) | 47.6(40.4, 54.8) | 0.5(-8.5, 9.5) |
| Haiti | 2017 | LAC | L | 4.1(3.2, 5.0) | 2.3(1.4, 3.1) | 7.6(5.6, 9.6) | 5.3(3.1, 7.5) |
| India | 2016 | SA | LM | 17.0(16.7, 17.4) | 11.3(11.0, 11.5) | 31.9(31.0, 32.8) | 20.6(19.7, 21.6) |
| Indonesia | 2017 | EAP | LM | 51.3(49.8, 52.7) | 45.2(43.1, 47.3) | 57.7(55.7, 59.7) | 12.5(9.6, 15.4) |
| Iraq | 2018 | MENA | UM | 41.3(38.8, 43.8) | 35.8(29.1, 42.5) | 43.9(41.5, 46.2) | 8.1(0.9, 15.2) |
| Kiribati | 2018 | EAP | LM | 7.2(5.5, 9.0) | 3.8(2.4, 5.3) | 10.3(7.2, 13.4) | 6.5(3.0, 9.9) |
| Kyrgyzstan* | 2018 | ECA | LM | 57.1(54.4, 59.8) | 57.7(54.4, 61.1) | 55.8(51.2, 60.3) | -2.0(-7.6, 3.7) |
| Lao | 2017 | EAP | LM | 23.8(22.4, 25.2) | 15.4(13.9, 16.9) | 46.7(43.7, 49.7) | 31.3(28.0, 34.6) |
| Lesotho | 2018 | SSA | LM | 3.9(2.8, 5.0) | 2.1(1.1, 3.1) | 7.4(4.9, 10.0) | 5.3(2.5, 8.0) |
| Liberia | 2019 | SSA | L | 1.0(0.4, 1.5) | 0.0(0.0, 0.0) | 1.8(0.8, 2.8) | 1.8(0.8, 2.8) |
| Madagascar | 2018 | SSA | L | 2.0(1.7, 2.4) | 1.1(0.8, 1.4) | 5.7(4.4, 7.1) | 4.6(3.2, 6.0) |
| Malawi | 2020 | SSA | L | 5.1(4.4, 5.9) | 3.7(3.2, 4.2) | 14.8(10.2, 19.4) | 11.1(6.4, 15.7) |
| Maldives | 2017 | SA | UM | 69.6(66.6, 72.6) | 74.4(72.0, 76.9) | 60.7(53.7, 67.7) | -13.7(-21.2, -6.3) |
| Mali | 2018 | SSA | L | 3.6(2.9, 4.2) | 2.5(1.9, 3.1) | 7.8(5.6, 9.9) | 5.2(3.0, 7.5) |
| Mongolia | 2018 | EAP | LM | 28.7(25.2, 32.2) | 13.2(10.3, 16.1) | 36.1(31.3, 40.9) | 22.9(17.3, 28.5) |
| Myanmar | 2016 | EAP | LM | 12.7(11.2, 14.3) | 8.0(6.4, 9.6) | 29.3(24.6, 34.0) | 21.3(16.3, 26.3) |
| Nepal | 2019 | SA | L | 33.2(30.8, 35.5) | 27.0(23.8, 30.2) | 36.5(33.3, 39.7) | 9.5(5.0, 14.0) |
| Nigeria | 2018 | SSA | LM | 5.6(4.9, 6.3) | 2.1(1.7, 2.4) | 11.2(9.6, 12.9) | 9.2(7.5, 10.8) |
| PNG | 2016-18 | SA | LM | 4.8(2.3, 7.4) | 3.4(0.6, 6.3) | 16.9(12.4, 21.3) | 13.4(8.1, 18.7) |
| Pakistan | 2018 | EAP | LM | 13.5(11.8, 15.1) | 9.0(6.9, 11.0) | 23.2(20.4, 25.9) | 14.2(10.8, 17.6) |
| Palestine* | 2019 | MENA | LM | 46.0(43.8, 48.3) | 47.4(44.1, 50.7) | 45.6(42.8, 48.4) | -1.8(-6.1, 2.6) |
| Paraguay | 2016 | LAC | UM | 53.4(50.1, 56.6) | 40.0(36.2, 43.7) | 62.9(57.9, 67.8) | 22.9(16.7, 29.1) |
| Philippines | 2017 | EAP | LM | 27.8(25.8, 29.8) | 25.2(22.4, 27.9) | 31.1(28.0, 34.2) | 5.9(1.8, 10.0) |
| Rwanda | 2020 | SSA | L | 10.3(9.2, 11.4) | 8.9(7.8, 10.0) | 16.7(13.0, 20.3) | 7.8(3.9, 11.6) |
| Samoa | 2019 | EAP | L | 17.3(14.6, 19.9) | 14.9(11.0, 18.7) | 18.5(15.0, 21.9) | 3.6(-1.6, 8.8) |
| STP* | 2019 | SSA | LM | 21.6(18.7, 24.4) | 19.5(16.4, 22.6) | 32.4(26.6, 38.2) | 12.9(6.3, 19.5) |
| Senegal | 2019 | SSA | L | 6.2(4.5, 7.9) | 3.1(2.2, 4.1) | 11.7(7.1, 16.3) | 8.6(3.8, 13.3) |
| Sierra Leone | 2019 | SSA | L | 1.3(1.0, 1.7) | 0.4(0.1, 0.7) | 3.1(2.1, 4.2) | 2.7(1.6, 3.8) |
| South Africa | 2016 | SSA | UM | 20.2(17.5, 23.0) | 8.4(6.6, 10.3) | 27.0(22.8, 31.1) | 18.5(14.0, 23.1) |
| Suriname | 2018 | LAC | UM | 39.7(36.7, 42.6) | 27.3(23.7, 31.0) | 46.7(42.7, 50.7) | 19.3(13.9, 24.8) |
| Tajikistan | 2017 | ECA | LM | 38.5(35.7, 41.3) | 34.9(31.6, 38.2) | 52.1(47.1, 57.1) | 17.2(11.2, 23.2) |
| Timor-Leste | 2016 | EAP | LM | 9.5(8.1, 11.0) | 4.5(3.1, 6.0) | 22.0(18.3, 25.6) | 17.4(13.5, 21.4) |
| Togo | 2017 | SSA | L | 4.2(3.0, 5.3) | 0.7(0.3, 1.1) | 9.8(7.2, 12.4) | 9.1(6.4, 11.7) |
| Tonga* | 2019 | EAP | LM | 43.2(38.7, 47.8) | 42.8(37.5, 48.1) | 44.9(36.5, 53.2) | 2.1(-7.9, 12.0) |
| Tunisia | 2018 | MENA | LM | 70.9(68.4, 73.4) | 59.7(55.5, 63.9) | 77.3(74.5, 80.0) | 17.5(12.5, 22.5) |
| Tuvalu* | 2019 | EAP | UM | 28.0(20.9, 35.1) | 22.4(13.2, 31.6) | 30.8(21.2, 40.5) | 8.4(-5.2, 22.0) |
| Uganda | 2016 | SSA | L | 3.5(2.9, 4.1) | 2.0(1.5, 2.5) | 9.2(7.3, 11.0) | 7.2(5.3, 9.1) |
| Zambia | 2018 | SSA | LM | 1.7(1.2, 2.2) | 0.5(0.2, 0.7) | 4.0(2.6, 5.4) | 3.6(2.2, 5.0) |
| Zimbabwe | 2019 | SSA | L | 11.1(9.7, 12.5) | 7.9(6.3, 9.4) | 18.9(15.9, 21.9) | 11.0(7.7, 14.4) |

**Note:**

1. World Bank region group: “EAP” represents “East Asia and Pacific”; “ECA” represents “Europe and Central Asia”; “LAC” represents “Latin America and the Caribbean”; “MENA” represents “Middle East and North Africa”; “SA” represents “South Asia”; “SSA” represents “Sub-Saharan Africa”.

2. Income group: “L” represents “Low-income country”; “LM” represents “Lower-middle income country”; “UM” represents “Upper-middle income country”.

*. The disparity in prevalence between young children in urban and rural areas is not statistically significant at the 0.05 level.

**Table S7.** National prevalence of young children living in households with preparedness and associated disparities by household wealth quintile

| **Country** | **Year** | **WB region^1^** | **Income group^2^** | **Wealth quintile** | | | | | **Difference (richest-poorest)** |
| --- | --- | --- | --- | --- | --- | --- | --- | --- | --- |
|  |  |  |  | **Poorest (%)** | **2 (%)** | **3 (%)** | **4 (%)** | **Richest (%)** |  |
| Algeria | 2019 | MENA | UM | 18.0(15.5, 20.5) | 33.6(30.8, 36.4) | 50.2(47.2, 53.2) | 65.0(62.0, 68.1) | 75.7(72.4, 79.0) | 57.7(53.6, 61.8) |
| Angola | 2016 | SSA | UM | 0.0(0.0, 0.0) | 0.4(0.1, 0.7) | 3.5(2.4, 4.5) | 10.4(7.3, 13.5) | 39.6(32.8, 46.4) | 39.6(32.8, 46.5) |
| Armenia | 2016 | ECA | LM | 28.1(21.4, 34.9) | 52.0(45.3, 58.6) | 72.6(65.4, 79.8) | 77.3(70.4, 84.1) | 84.0(77.5, 90.6) | 55.9(46.5, 65.4) |
| Bangladesh | 2019 | SA | LM | 2.2(1.7, 2.7) | 11.2(10.1, 12.3) | 26.1(24.5, 27.8) | 39.5(37.6, 41.5) | 62.1(59.8, 64.5) | 59.9(57.6, 62.3) |
| Benin | 2018 | SSA | L | 0.0(0.0, 0.0) | 0.0(0.0, 0.1) | 0.0(0.0, 0.0) | 0.4(0.0, 0.7) | 7.3(5.6, 9.1) | 7.3(5.6, 9.1) |
| Burundi | 2017 | SSA | L | 0.0(0.0, 0.0) | 0.1(0.0, 0.2) | 0.0(0.0, 0.1) | 0.8(0.3, 1.3) | 6.7(4.9, 8.6) | 6.7(4.9, 8.6) |
| CAR | 2018 | SSA | L | 0.0(0.0, 0.0) | 0.0(0.0, 0.0) | 0.0(0.0, 0.0) | 0.5(-0.3, 1.3) | 5.1(3.7, 6.6) | 5.1(3.6, 6.6) |
| Cameroon | 2018 | SSA | LM | 0.0(0.0, 0.0) | 1.0(0.4, 1.7) | 3.4(2.1, 4.8) | 9.9(7.7, 12.0) | 39.5(35.3, 43.7) | 39.5(35.3, 43.8) |
| Chad | 2019 | SSA | L | 0.0(0.0, 0.0) | 0.0(0.0, 0.1) | 0.0(0.0, 0.1) | 0.5(0.2, 0.9) | 9.9(8.0, 11.8) | 9.9(8.0, 11.8) |
| Congo DR | 2018 | SSA | L | 0.0(0.0, 0.0) | 0.0(0.0, 0.0) | 0.1(0.0, 0.2) | 0.7(0.0, 1.4) | 8.8(5.9, 11.6) | 8.8(5.9, 11.6) |
| Cote d’Ivoire | 2016 | SSA | LM | 0.0(0.0, 0.1) | 0.3(0.0, 0.5) | 1.3(0.6, 2.0) | 4.7(2.9, 6.5) | 25.5(19.7, 31.2) | 25.4(19.7, 31.2) |
| Dominican Republic | 2019 | LAC | UM | 4.3(3.1, 5.4) | 18.0(15.3, 20.8) | 37.5(33.7, 41.2) | 60.2(55.5, 64.9) | 77.1(72.6, 81.7) | 72.9(68.2, 77.5) |
| Ethiopia | 2016 | SSA | L | 0.0(0.0, 0.0) | 0.0(0.0, 0.0) | 0.0(0.0, 0.0) | 0.0(0.0, 0.0) | 4.1(2.0, 6.2) | 4.1(2.0, 6.2) |
| Gambia | 2020 | SSA | L | 0.8(0.2, 1.4) | 0.7(0.2, 1.3) | 2.4(0.1, 4.7) | 4.3(2.2, 6.3) | 12.9(9.0, 16.8) | 12.1(8.2, 16.0) |
| Ghana | 2017 | SSA | LM | 0.6(0.1, 1.0) | 0.5(0.1, 0.9) | 2.7(1.3, 4.1) | 3.7(1.7, 5.8) | 29.2(24.9, 33.4) | 28.6(24.3, 32.9) |
| Guinea | 2018 | SSA | L | 0.1(-0.1, 0.4) | 0.1(-0.1, 0.4) | 0.9(0.3, 1.6) | 2.2(1.0, 3.3) | 14.0(10.7, 17.2) | 13.8(10.6, 17.1) |
| Guinea-Bissau | 2019 | SSA | L | 0.0(0.0, 0.0) | 0.1(-0.1, 0.3) | 0.5(-0.1, 1.0) | 2.0(0.8, 3.1) | 7.0(4.3, 9.8) | 7.0(4.3, 9.8) |
| Guyana | 2019 | LAC | UM | 16.6(12.1, 21.1) | 42.1(33.6, 50.6) | 58.3(48.6, 68.0) | 65.3(55.4, 75.2) | 82.1(76.3, 87.9) | 65.5(58.3, 72.7) |
| Haiti | 2017 | LAC | L | 0.0(0.0, 0.0) | 0.7(0.1, 1.3) | 1.7(0.8, 2.7) | 4.3(2.3, 6.3) | 20.1(16.3, 23.9) | 20.1(16.3, 23.9) |
| India | 2016 | SA | LM | 0.1(0.0, 0.1) | 2.2(2.0, 2.4) | 10.7(10.2, 11.1) | 29.5(28.7, 30.4) | 61.9(60.7, 63.1) | 61.8(60.6, 63.0) |
| Indonesia | 2017 | EAP | LM | 11.5(10.0, 12.9) | 38.9(36.2, 41.5) | 57.4(54.8, 59.9) | 71.3(69.0, 73.5) | 80.1(77.9, 82.3) | 68.6(66.0, 71.3) |
| Iraq | 2018 | MENA | UM | 21.4(18.5, 24.3) | 36.2(32.0, 40.5) | 44.2(39.6, 48.8) | 52.0(48.2, 55.7) | 58.9(54.0, 63.8) | 37.5(31.8, 43.2) |
| Kiribati | 2018 | EAP | LM | 0.6(-0.3, 1.6) | 4.4(2.1, 6.7) | 3.5(1.4, 5.5) | 7.2(3.2, 11.2) | 22.8(16.1, 29.6) | 22.2(15.4, 29.0) |
| Kyrgyzstan* | 2018 | ECA | LM | 52.5(47.1, 57.9) | 59.4(54.4, 64.5) | 58.1(52.2, 63.9) | 60.0(54.8, 65.2) | 56.1(49.5, 62.8) | 3.6(-4.9, 12.2) |
| Lao | 2017 | EAP | LM | 0.4(0.1, 0.7) | 6.8(5.5, 8.1) | 19.7(17.4, 21.9) | 40.8(38.0, 43.7) | 70.5(67.4, 73.6) | 70.1(67.0, 73.2) |
| Lesotho | 2018 | SSA | LM | 0.0(0.0, 0.0) | 0.2(0.0, 0.5) | 1.4(0.0, 2.7) | 2.5(1.1, 3.9) | 19.4(13.7, 25.0) | 19.4(13.7, 25.0) |
| Liberia | 2019 | SSA | L | 0.0(0.0, 0.0) | 0.0(0.0, 0.0) | 0.1(-0.1, 0.3) | 0.3(-0.3, 0.8) | 5.5(2.4, 8.6) | 5.5(2.4, 8.6) |
| Madagascar | 2018 | SSA | L | 0.0(0.0, 0.0) | 0.0(0.0, 0.1) | 0.3(0.1, 0.5) | 2.1(1.3, 2.9) | 11.0(9.0, 13.0) | 11.0(9.0, 13.0) |
| Malawi | 2020 | SSA | L | 0.1(0.0, 0.1) | 0.9(0.5, 1.3) | 3.3(2.5, 4.1) | 5.7(4.2, 7.2) | 21.3(17.4, 25.2) | 21.2(17.3, 25.1) |
| Maldives* | 2017 | SA | UM | 63.8(59.3, 68.3) | 73.9(69.1, 78.8) | 78.1(73.6, 82.7) | 57.2(48.3, 66.1) | 72.2(62.4, 82.1) | 8.5(-2.4, 19.4) |
| Mali | 2018 | SSA | L | 0.2(-0.1, 0.5) | 1.3(0.5, 2.0) | 2.2(1.0, 3.4) | 4.5(3.0, 6.0) | 11.4(8.9, 13.9) | 11.2(8.7, 13.8) |
| Mongolia | 2018 | EAP | LM | 1.7(0.9, 2.6) | 6.9(4.9, 8.9) | 15.7(11.8, 19.6) | 44.1(37.9, 50.4) | 64.8(59.0, 70.6) | 63.1(57.2, 68.9) |
| Myanmar | 2016 | EAP | LM | 0.9(0.1, 1.8) | 3.7(2.2, 5.3) | 9.2(6.6, 11.9) | 21.1(16.9, 25.3) | 46.4(40.6, 52.3) | 45.5(39.5, 51.5) |
| Nepal | 2019 | SA | L | 9.7(7.8, 11.6) | 26.2(22.4, 30.0) | 36.0(31.9, 40.2) | 48.8(44.1, 53.6) | 53.2(47.0, 59.3) | 43.5(36.9, 50.0) |
| Nigeria | 2018 | SSA | LM | 0.1(0.0, 0.2) | 0.4(0.2, 0.6) | 1.6(1.1, 2.2) | 6.6(5.5, 7.6) | 24.6(21.7, 27.5) | 24.5(21.6, 27.4) |
| PNG | 2016-18 | SA | LM | 0.0(0.0, 0.0) | 0.1(-0.1, 0.3) | 0.1(0.0, 0.3) | 2.1(1.1, 3.1) | 25.2(13.7, 36.7) | 25.2(13.7, 36.7) |
| Pakistan | 2018 | EAP | LM | 0.2(-0.2, 0.7) | 1.4(0.7, 2.2) | 8.9(6.4, 11.3) | 22.9(19.1, 26.6) | 40.5(35.3, 45.8) | 40.3(35.0, 45.6) |
| Palestine | 2019 | MENA | LM | 23.2(19.6, 26.9) | 40.3(35.5, 45.1) | 44.2(39.9, 48.6) | 56.5(52.6, 60.5) | 72.7(68.8, 76.5) | 49.5(44.1, 54.8) |
| Paraguay | 2016 | LAC | UM | 10.7(8.3, 13.2) | 44.5(39.4, 49.7) | 71.5(66.4, 76.5) | 78.8(74.0, 83.7) | 90.2(86.8, 93.6) | 79.4(75.3, 83.6) |
| Philippines | 2017 | EAP | LM | 5.2(4.1, 6.3) | 15.2(13.2, 17.2) | 29.8(26.0, 33.6) | 49.9(45.6, 54.2) | 68.3(61.9, 74.7) | 63.1(56.5, 69.7) |
| Rwanda | 2020 | SSA | L | 0.7(0.2, 1.2) | 3.7(2.6, 4.9) | 9.0(7.3, 10.7) | 13.7(11.3, 16.0) | 27.3(23.3, 31.4) | 26.7(22.6, 30.8) |
| Samoa | 2019 | EAP | L | 0.8(0.0, 1.6) | 3.4(1.1, 5.7) | 9.2(5.8, 12.6) | 27.1(20.6, 33.6) | 61.5(53.4, 69.7) | 60.7(52.6, 68.9) |
| STP | 2019 | SSA | LM | 0.8(0.0, 1.6) | 3.4(1.1, 5.7) | 9.2(5.8, 12.6) | 27.1(20.6, 33.6) | 61.5(53.4, 69.7) | 60.7(52.6, 68.9) |
| Senegal | 2019 | SSA | L | 2.2(0.9, 3.5) | 2.9(1.6, 4.1) | 2.0(1.0, 3.0) | 4.1(2.0, 6.1) | 24.0(16.3, 31.8) | 21.8(13.9, 29.7) |
| Sierra Leone | 2019 | SSA | L | 0.0(0.0, 0.0) | 0.0(0.0, 0.1) | 0.4(0.0, 0.7) | 1.1(0.3, 1.9) | 7.0(4.6, 9.4) | 7.0(4.6, 9.4) |
| South Africa | 2016 | SSA | UM | 2.2(1.1, 3.3) | 4.9(3.3, 6.5) | 10.8(7.8, 13.8) | 34.9(28.5, 41.4) | 74.0(67.1, 80.9) | 71.8(64.8, 78.9) |
| Suriname | 2018 | LAC | UM | 12.9(9.8, 16.0) | 39.3(34.1, 44.4) | 49.9(43.4, 56.4) | 66.5(59.6, 73.4) | 72.3(64.3, 80.4) | 59.5(50.7, 68.3) |
| Tajikistan | 2017 | ECA | LM | 22.7(17.9, 27.4) | 27.6(22.4, 32.7) | 39.8(34.9, 44.7) | 45.0(39.5, 50.5) | 59.1(54.7, 63.6) | 36.5(30.1, 42.8) |
| Timor-Leste | 2016 | EAP | LM | 0.0(0.0, 0.1) | 0.6(0.1, 1.2) | 3.3(2.1, 4.6) | 10.7(6.7, 14.7) | 34.0(29.6, 38.5) | 34.0(29.5, 38.4) |
| Togo | 2017 | SSA | L | 0.0(0.0, 0.0) | 0.0(0.0, 0.1) | 0.4(-0.2, 0.9) | 1.9(0.2, 3.6) | 21.2(17.0, 25.4) | 21.2(17.0, 25.4) |
| Tonga | 2019 | EAP | LM | 18.8(12.2, 25.4) | 35.8(25.7, 45.9) | 47.6(35.6, 59.6) | 59.1(48.3, 70.0) | 67.4(58.1, 76.7) | 48.6(37.4, 59.8) |
| Tunisia | 2018 | MENA | LM | 52.1(47.1, 57.1) | 62.9(58.0, 67.9) | 70.5(65.0, 75.9) | 82.7(78.8, 86.6) | 87.8(83.8, 91.8) | 35.7(29.3, 42.1) |
| Tuvalu | 2019 | EAP | UM | 9.5(3.3, 15.7) | 20.1(8.7, 31.6) | 17.7(7.9, 27.5) | 44.7(31.1, 58.3) | 53.6(32.3, 74.9) | 44.0(21.2, 66.9) |
| Uganda | 2016 | SSA | L | 0.0(0.0, 0.1) | 0.1(0.0, 0.3) | 0.8(0.3, 1.3) | 3.0(2.0, 4.0) | 14.6(12.2, 17.1) | 14.6(12.1, 17.0) |
| Zambia | 2018 | SSA | LM | 0.0(0.0, 0.1) | 0.2(0.0, 0.4) | 0.4(0.0, 0.7) | 0.9(0.3, 1.4) | 9.4(6.6, 12.1) | 9.3(6.6, 12.1) |
| Zimbabwe | 2019 | SSA | L | 0.7(0.1, 1.2) | 4.6(2.9, 6.3) | 13.1(10.2, 16.0) | 12.4(9.7, 15.1) | 31.0(26.6, 35.5) | 30.4(25.9, 34.9) |

**Note:**

1. World Bank region group: “EAP” represents “East Asia and Pacific”; “ECA” represents “Europe and Central Asia”; “LAC” represents “Latin America and the Caribbean”; “MENA” represents “Middle East and North Africa”; “SA” represents “South Asia”; “SSA” represents “Sub-Saharan Africa”.

2. Income group: “L” represents “Low-income country”; “LM” represents “Lower-middle income country”; “UM” represents “Upper-middle income country”.

*. The disparity in prevalence between young children living in the wealthiest and poorest quintiles is not statistically significant at the 0.05 level.

**Table S8.** National prevalence of young children living in households with adequate quarantine condition and associated disparities by place of residence

| Country | Year | WB region^1^ | Income group^2^ | National (%) | Rural (%) | Urban (%) | Difference  (urban-rural) |
| --- | --- | --- | --- | --- | --- | --- | --- |
| Algeria | 2019 | MENA | UM | 69.8(68.2, 71.3) | 64.4(61.8, 67.1) | 73.7(71.9, 75.5) | 9.2(6.0, 12.5) |
| Angola | 2016 | SSA | UM | 53.2(51.4, 55.1) | 49.2(46.6, 51.9) | 55.8(53.2, 58.5) | 6.6(2.9, 10.3) |
| Armenia* | 2016 | ECA | LM | 87.4(85.0, 89.8) | 89.0(86.4, 91.7) | 86.2(82.6, 89.8) | -2.9(-7.4, 1.6) |
| Bangladesh* | 2019 | SA | LM | 71.6(70.8, 72.5) | 71.6(70.7, 72.6) | 71.7(69.7, 73.8) | 0.1(-2.2, 2.4) |
| Benin* | 2018 | SSA | L | 63.1(61.4, 64.8) | 62.2(60.1, 64.3) | 64.4(61.7, 67.2) | 2.2(-1.3, 5.8) |
| Burundi* | 2017 | SSA | L | 74.9(73.6, 76.2) | 74.8(73.5, 76.2) | 75.7(71.9, 79.5) | 0.9(-3.1, 4.9) |
| CAR* | 2018 | SSA | L | 56.2(53.9, 58.6) | 55.5(52.8, 58.3) | 57.9(53.3, 62.6) | 2.4(-3.0, 7.8) |
| Cameroon* | 2018 | SSA | LM | 66.8(64.5, 69.1) | 66.9(63.5, 70.3) | 66.7(63.9, 69.6) | -0.2(-4.6, 4.3) |
| Chad* | 2019 | SSA | L | 61.5(60.0, 63.0) | 61.7(60.0, 63.4) | 60.6(57.7, 63.4) | -1.1(-4.4, 2.2) |
| Congo DR | 2018 | SSA | L | 59.8(57.6, 62.1) | 64.0(61.6, 66.3) | 53.3(49.6, 57.0) | -10.7(-15.1, -6.3) |
| Cote d’Ivoire | 2016 | SSA | LM | 60.3(58.1, 62.5) | 62.3(59.5, 65.1) | 57.3(53.6, 60.9) | -5.0(-9.6, -0.4) |
| Dominican Republic | 2019 | LAC | UM | 79.6(77.7, 81.4) | 74.2(70.3, 78.1) | 81.4(79.4, 83.4) | 7.2(2.8, 11.6) |
| Ethiopia | 2016 | SSA | L | 27.1(24.7, 29.4) | 24.0(21.6, 26.3) | 52.1(45.8, 58.3) | 28.1(21.4, 34.8) |
| Gambia* | 2020 | SSA | L | 76.6(74.2, 79.1) | 78.4(75.3, 81.4) | 75.7(72.4, 79.1) | -2.6(-7.2, 1.9) |
| Ghana* | 2017 | SSA | LM | 48.2(45.8, 50.6) | 49.3(46.4, 52.3) | 46.7(42.9, 50.6) | -2.6(-7.5, 2.3) |
| Guinea* | 2018 | SSA | L | 66.9(64.6, 69.2) | 68.1(65.2, 71.0) | 63.9(60.2, 67.6) | -4.2(-8.9, 0.6) |
| Guinea-Bissau | 2019 | SSA | L | 80.1(78.3, 82.0) | 85.8(83.8, 87.9) | 63.9(60.1, 67.7) | -21.9(-26.2, -17.6) |
| Guyana* | 2019 | LAC | UM | 75.6(72.5, 78.8) | 75.7(71.9, 79.6) | 75.3(70.0, 80.6) | -0.4(-7.0, 6.1) |
| Haiti* | 2017 | LAC | L | 55.1(52.9, 57.2) | 55.8(53.0, 58.5) | 53.8(50.4, 57.3) | -2.0(-6.4, 2.5) |
| India | 2016 | SA | LM | 47.2(46.8, 47.7) | 45.7(45.2, 46.1) | 51.3(50.3, 52.3) | 5.6(4.5, 6.7) |
| Indonesia* | 2017 | EAP | LM | 81.1(80.2, 82.1) | 81.7(80.4, 83.1) | 80.5(79.2, 81.8) | -1.2(-3.2, 0.7) |
| Iraq* | 2018 | MENA | UM | 49.0(46.7, 51.4) | 45.8(40.3, 51.3) | 50.6(48.1, 53.1) | 4.8(-1.3, 10.8) |
| Kiribati | 2018 | EAP | LM | 43.8(40.8, 46.9) | 48.7(44.6, 52.7) | 39.5(35.0, 44.0) | -9.2(-15.3, -3.1) |
| Kyrgyzstan* | 2018 | ECA | LM | 76.7(74.0, 79.4) | 76.7(73.2, 80.2) | 76.7(72.8, 80.6) | 0.0(-5.3, 5.3) |
| Lao | 2017 | EAP | LM | 46.9(45.2, 48.6) | 40.2(38.2, 42.3) | 64.8(62.1, 67.5) | 24.6(21.2, 28.0) |
| Lesotho | 2018 | SSA | LM | 54.1(51.6, 56.5) | 48.5(45.5, 51.4) | 65.1(61.1, 69.2) | 16.7(11.7, 21.7) |
| Liberia | 2019 | SSA | L | 68.2(65.5, 70.9) | 72.4(70.1, 74.7) | 64.5(59.7, 69.2) | -8.0(-13.2, -2.7) |
| Madagascar | 2018 | SSA | L | 34.7(33.2, 36.2) | 32.8(31.0, 34.5) | 42.1(39.2, 45.0) | 9.3(5.9, 12.7) |
| Malawi | 2020 | SSA | L | 74.1(72.8, 75.4) | 73.1(71.8, 74.5) | 80.9(77.2, 84.5) | 7.7(3.9, 11.6) |
| Maldives | 2017 | SA | UM | 79.0(76.3, 81.7) | 86.7(84.9, 88.5) | 64.8(58.1, 71.4) | -21.9(-28.8, -15.0) |
| Mali* | 2018 | SSA | L | 71.6(69.6, 73.6) | 71.9(69.6, 74.2) | 70.4(65.9, 74.9) | -1.5(-6.6, 3.6) |
| Mongolia | 2018 | EAP | LM | 54.8(51.4, 58.1) | 46.7(40.9, 52.6) | 56.8(52.9, 60.7) | 10.1(3.0, 17.1) |
| Myanmar | 2016 | EAP | LM | 45.6(43.2, 48.0) | 42.9(40.2, 45.5) | 55.3(50.0, 60.7) | 12.5(6.5, 18.5) |
| Nepal | 2019 | SA | L | 72.7(70.7, 74.6) | 67.7(64.5, 70.8) | 75.4(73.0, 77.8) | 7.7(3.8, 11.7) |
| Nigeria* | 2018 | SSA | LM | 59.9(58.6, 61.1) | 60.1(58.4, 61.7) | 59.6(57.8, 61.4) | -0.5(-2.9, 2.0) |
| PNG* | 2016-18 | SA | LM | 64.0(61.6, 66.5) | 64.6(62.0, 67.2) | 59.5(53.4, 65.7) | -5.1(-11.7, 1.6) |
| Pakistan | 2018 | EAP | LM | 31.5(29.3, 33.8) | 28.6(25.7, 31.5) | 37.8(34.2, 41.4) | 9.2(4.6, 13.8) |
| Palestine* | 2019 | MENA | LM | 65.1(62.9, 67.3) | 64.2(61.0, 67.4) | 65.4(62.6, 68.2) | 1.2(-3.0, 5.5) |
| Paraguay | 2016 | LAC | UM | 73.8(70.9, 76.8) | 68.3(64.7, 71.8) | 77.6(73.2, 81.9) | 9.3(3.7, 14.9) |
| Philippines* | 2017 | EAP | LM | 47.6(45.4, 49.7) | 47.3(44.6, 50.1) | 47.9(44.3, 51.4) | 0.5(-4.0, 5.1) |
| Rwanda* | 2020 | SSA | L | 83.0(81.7, 84.4) | 83.2(81.8, 84.6) | 82.5(78.4, 86.7) | -0.6(-5.0, 3.8) |
| Samoa | 2019 | EAP | L | 40.5(36.8, 44.2) | 38.2(34.0, 42.3) | 52.4(45.7, 59.1) | 14.2(6.3, 22.1) |
| STP* | 2019 | SSA | LM | 70.2(67.3, 73.2) | 72.2(68.1, 76.2) | 69.3(65.4, 73.2) | -2.9(-8.6, 2.7) |
| Senegal* | 2019 | SSA | L | 70.3(67.9, 72.7) | 69.8(66.9, 72.6) | 71.2(66.9, 75.4) | 1.4(-3.7, 6.6) |
| Sierra Leone | 2019 | SSA | L | 74.6(73.0, 76.1) | 77.5(75.5, 79.4) | 69.2(66.6, 71.7) | -8.3(-11.5, -5.1) |
| South Africa* | 2016 | SSA | UM | 74.4(71.7, 77.1) | 75.2(71.8, 78.5) | 74.0(70.2, 77.7) | -1.2(-6.2, 3.8) |
| Suriname | 2018 | LAC | UM | 70.6(68.2, 73.1) | 57.5(53.8, 61.3) | 77.4(74.5, 80.3) | 19.9(15.1, 24.7) |
| Tajikistan | 2017 | ECA | LM | 60.8(58.2, 63.4) | 58.6(55.5, 61.8) | 69.0(65.1, 72.9) | 10.4(5.4, 15.4) |
| Timor-Leste* | 2016 | EAP | LM | 72.9(71.0, 74.8) | 71.8(69.5, 74.0) | 75.6(72.1, 79.1) | 3.8(-0.3, 8.0) |
| Togo | 2017 | SSA | L | 64.6(62.2, 67.1) | 67.6(64.7, 70.6) | 59.8(55.7, 64.0) | -7.8(-12.9, -2.7) |
| Tonga* | 2019 | EAP | LM | 75.2(71.6, 78.7) | 74.7(70.5, 78.9) | 77.0(71.2, 82.7) | 2.3(-4.9, 9.5) |
| Tunisia | 2018 | MENA | LM | 80.2(78.2, 82.3) | 73.7(70.4, 77.1) | 83.9(81.5, 86.3) | 10.2(6.0, 14.3) |
| Tuvalu* | 2019 | EAP | UM | 43.9(36.4, 51.4) | 41.8(31.8, 51.9) | 44.9(34.8, 55.0) | 3.1(-11.5, 17.7) |
| Uganda | 2016 | SSA | L | 54.0(52.5, 55.5) | 52.3(50.5, 54.1) | 60.3(57.2, 63.3) | 8.0(4.4, 11.5) |
| Zambia | 2018 | SSA | LM | 57.3(55.6, 59.1) | 54.5(52.3, 56.6) | 62.5(59.6, 65.4) | 8.0(4.4, 11.7) |
| Zimbabwe | 2019 | SSA | L | 70.1(68.4, 71.9) | 68.7(66.7, 70.8) | 73.5(70.4, 76.6) | 4.8(1.1, 8.5) |

**Note:**

1. World Bank region group: “EAP” represents “East Asia and Pacific”; “ECA” represents “Europe and Central Asia”; “LAC” represents “Latin America and the Caribbean”; “MENA” represents “Middle East and North Africa”; “SA” represents “South Asia”; “SSA” represents “Sub-Saharan Africa”.

2. Income group: “L” represents “Low-income country”; “LM” represents “Lower-middle income country”; “UM” represents “Upper-middle income country”.

*. The disparity in prevalence between young children in urban and rural areas is not statistically significant at the 0.05 level.

**Table S9.** National prevalence of young children living in households with adequate quarantine condition and associated disparities by household wealth quintile

| **Country** | **Year** | **WB region^1^** | **Income group^2^** | **Wealth quintile** | | | | | **Difference (richest-poorest)** |
| --- | --- | --- | --- | --- | --- | --- | --- | --- | --- |
|  |  |  |  | **Poorest (%)** | **2 (%)** | **3 (%)** | **4 (%)** | **Richest (%)** |  |
| Algeria | 2019 | MENA | UM | 51.6(48.2, 55.1) | 63.5(60.0, 66.9) | 72.8(70.2, 75.4) | 83.0(80.6, 85.5) | 88.5(85.9, 91.0) | 36.8(32.7, 41.0) |
| Angola | 2016 | SSA | UM | 45.7(42.3, 49.0) | 50.5(47.5, 53.5) | 48.9(45.5, 52.2) | 54.2(50.1, 58.3) | 75.4(70.1, 80.6) | 29.7(23.5, 35.9) |
| Armenia* | 2016 | ECA | LM | 85.8(81.1, 90.5) | 88.5(83.9, 93.1) | 87.9(82.4, 93.4) | 87.7(81.3, 94.1) | 87.1(80.9, 93.3) | 1.3(-6.4, 9.1) |
| Bangladesh | 2019 | SA | LM | 53.0(51.1, 54.9) | 63.8(62.1, 65.6) | 77.4(75.8, 79.1) | 81.3(79.6, 83.0) | 84.3(82.4, 86.2) | 31.3(28.6, 34.0) |
| Benin | 2018 | SSA | L | 56.2(52.5, 59.9) | 61.5(58.5, 64.6) | 62.3(58.9, 65.8) | 65.7(62.7, 68.7) | 71.0(67.7, 74.2) | 14.8(9.7, 19.8) |
| Burundi | 2017 | SSA | L | 64.6(61.7, 67.5) | 74.6(72.2, 77.0) | 76.5(73.8, 79.2) | 78.9(76.6, 81.2) | 82.7(80.0, 85.4) | 18.1(14.2, 22.0) |
| CAR | 2018 | SSA | L | 49.8(45.1, 54.5) | 52.0(47.6, 56.4) | 56.3(52.4, 60.2) | 59.2(54.5, 63.8) | 68.8(64.3, 73.3) | 19.0(12.6, 25.3) |
| Cameroon | 2018 | SSA | LM | 56.8(50.5, 63.0) | 68.4(64.5, 72.4) | 68.0(64.5, 71.6) | 67.0(62.3, 71.7) | 77.3(73.4, 81.2) | 20.5(13.2, 27.8) |
| Chad | 2019 | SSA | L | 42.6(39.6, 45.5) | 55.5(52.5, 58.5) | 67.0(64.4, 69.7) | 75.5(72.7, 78.3) | 70.5(67.7, 73.3) | 28.0(23.8, 32.1) |
| Congo DR* | 2018 | SSA | L | 56.0(52.7, 59.3) | 64.1(60.7, 67.5) | 66.8(63.4, 70.3) | 58.7(54.0, 63.4) | 52.1(46.2, 58.0) | -3.9(-10.6, 2.9) |
| Cote d’Ivoire* | 2016 | SSA | LM | 59.9(55.9, 63.8) | 61.5(57.7, 65.4) | 64.6(60.6, 68.5) | 51.5(47.0, 56.1) | 64.4(58.2, 70.6) | 4.5(-2.9, 11.9) |
| Dominican Republic | 2019 | LAC | UM | 62.4(58.7, 66.0) | 69.7(65.7, 73.8) | 84.9(81.7, 88.1) | 92.3(89.9, 94.7) | 97.4(96.1, 98.8) | 35.1(31.2, 38.9) |
| Ethiopia | 2016 | SSA | L | 13.2(10.3, 16.0) | 20.3(17.2, 23.5) | 26.3(21.8, 30.8) | 38.3(33.8, 42.8) | 47.7(42.5, 53.0) | 34.5(28.7, 40.4) |
| Gambia | 2020 | SSA | L | 72.4(68.9, 76.0) | 75.7(71.0, 80.4) | 75.3(69.9, 80.8) | 77.3(71.6, 83.0) | 84.3(78.8, 89.8) | 11.8(5.1, 18.5) |
| Ghana | 2017 | SSA | LM | 48.2(44.0, 52.3) | 40.0(34.0, 45.9) | 41.3(36.9, 45.7) | 43.4(38.8, 48.0) | 70.0(66.0, 74.0) | 21.8(16.0, 27.7) |
| Guinea | 2018 | SSA | L | 56.8(52.5, 61.2) | 70.6(66.3, 74.8) | 75.8(71.4, 80.3) | 63.4(59.2, 67.6) | 70.2(65.4, 75.0) | 13.4(6.9, 19.8) |
| Guinea-Bissau | 2019 | SSA | L | 80.9(76.9, 84.8) | 88.0(84.8, 91.2) | 88.6(85.9, 91.4) | 70.5(65.6, 75.4) | 64.7(59.4, 69.9) | -16.2(-22.7, -9.7) |
| Guyana | 2019 | LAC | UM | 52.2(46.0, 58.4) | 73.2(64.8, 81.7) | 80.6(71.9, 89.3) | 93.1(89.5, 96.7) | 95.4(92.2, 98.7) | 43.3(36.3, 50.3) |
| Haiti | 2017 | LAC | L | 47.0(43.0, 51.0) | 56.6(52.4, 60.7) | 50.6(45.9, 55.3) | 54.4(49.6, 59.2) | 74.7(70.3, 79.0) | 27.6(21.7, 33.6) |
| India | 2016 | SA | LM | 31.4(30.7, 32.0) | 41.1(40.3, 41.8) | 47.3(46.5, 48.2) | 55.4(54.3, 56.5) | 73.4(72.3, 74.5) | 42.0(40.8, 43.3) |
| Indonesia | 2017 | EAP | LM | 64.2(61.9, 66.6) | 78.6(76.5, 80.6) | 83.7(81.9, 85.5) | 86.9(85.2, 88.6) | 93.2(91.8, 94.7) | 29.0(26.2, 31.8) |
| Iraq | 2018 | MENA | UM | 35.2(32.0, 38.5) | 43.2(39.2, 47.3) | 50.0(45.4, 54.7) | 58.2(54.4, 62.0) | 63.7(59.4, 67.9) | 28.4(23.1, 33.8) |
| Kiribati | 2018 | EAP | LM | 40.6(34.8, 46.3) | 49.3(43.1, 55.4) | 41.4(35.0, 47.8) | 36.9(29.8, 43.9) | 51.9(44.0, 59.8) | 11.3(1.5, 21.1) |
| Kyrgyzstan | 2018 | ECA | LM | 68.8(62.6, 75.1) | 78.3(73.5, 83.1) | 79.5(74.0, 85.1) | 80.2(76.3, 84.2) | 78.6(72.6, 84.7) | 9.8(1.1, 18.5) |
| Lao | 2017 | EAP | LM | 20.5(18.1, 22.8) | 33.5(30.7, 36.3) | 49.1(46.0, 52.1) | 64.6(61.6, 67.6) | 83.7(81.1, 86.2) | 63.2(59.7, 66.7) |
| Lesotho | 2018 | SSA | LM | 36.5(31.9, 41.0) | 43.6(38.3, 48.9) | 57.4(52.1, 62.7) | 61.0(55.4, 66.6) | 79.3(74.1, 84.4) | 42.8(35.9, 49.7) |
| Liberia | 2019 | SSA | L | 67.9(63.6, 72.2) | 76.4(72.6, 80.2) | 76.5(72.4, 80.6) | 59.7(52.5, 66.8) | 57.7(48.7, 66.7) | -10.2(-20.2, -0.2) |
| Madagascar | 2018 | SSA | L | 22.7(20.7, 24.6) | 25.4(23.2, 27.5) | 33.3(30.5, 36.1) | 45.9(42.7, 49.0) | 59.3(54.8, 63.8) | 36.6(31.7, 41.5) |
| Malawi | 2020 | SSA | L | 62.6(59.9, 65.3) | 70.2(67.3, 73.2) | 78.9(76.6, 81.3) | 80.1(77.6, 82.6) | 86.1(83.3, 88.9) | 23.5(19.6, 27.4) |
| Maldives* | 2017 | SA | UM | 80.2(76.0, 84.3) | 86.5(82.8, 90.1) | 86.0(81.7, 90.3) | 61.6(53.1, 70.1) | 76.9(68.8, 85.0) | -3.3(-12.5, 5.9) |
| Mali | 2018 | SSA | L | 66.9(62.5, 71.4) | 70.0(66.1, 73.9) | 74.2(70.6, 77.7) | 68.3(64.1, 72.5) | 79.8(75.9, 83.7) | 12.9(6.9, 18.9) |
| Mongolia | 2018 | EAP | LM | 38.8(29.8, 47.8) | 31.8(25.7, 37.8) | 42.9(36.9, 48.9) | 58.8(52.8, 64.8) | 69.3(63.8, 74.9) | 30.5(20.0, 41.0) |
| Myanmar | 2016 | EAP | LM | 35.5(31.4, 39.5) | 39.6(34.8, 44.3) | 44.3(39.2, 49.5) | 53.5(48.6, 58.4) | 68.6(63.5, 73.6) | 33.1(26.6, 39.6) |
| Nepal | 2019 | SA | L | 60.0(56.6, 63.5) | 68.8(64.7, 72.9) | 73.8(69.6, 77.9) | 80.0(76.3, 83.6) | 85.3(82.0, 88.6) | 25.3(20.5, 30.1) |
| Nigeria | 2018 | SSA | LM | 55.6(53.1, 58.1) | 58.1(55.2, 61.0) | 61.0(58.6, 63.4) | 58.7(56.1, 61.4) | 68.1(65.4, 70.7) | 12.4(8.8, 16.1) |
| PNG | 2016-18 | SA | LM | 55.2(50.5, 59.9) | 57.8(52.5, 63.1) | 70.4(66.1, 74.6) | 65.9(61.8, 69.9) | 71.8(65.0, 78.5) | 16.5(8.3, 24.7) |
| Pakistan | 2018 | EAP | LM | 11.8(9.3, 14.4) | 25.2(21.4, 29.1) | 30.2(25.9, 34.6) | 40.8(36.2, 45.5) | 55.8(50.6, 60.9) | 43.9(38.2, 49.7) |
| Palestine | 2019 | MENA | LM | 43.3(38.6, 48.0) | 63.6(59.0, 68.2) | 65.4(61.6, 69.3) | 72.7(69.0, 76.4) | 86.6(83.4, 89.8) | 43.3(37.6, 49.0) |
| Paraguay | 2016 | LAC | UM | 46.0(40.7, 51.2) | 71.5(66.3, 76.7) | 81.5(77.0, 86.0) | 87.2(83.7, 90.8) | 97.2(95.7, 98.6) | 51.2(45.8, 56.7) |
| Philippines | 2017 | EAP | LM | 28.0(25.3, 30.7) | 41.7(38.4, 45.0) | 49.4(44.7, 54.1) | 60.9(56.8, 65.1) | 79.1(72.5, 85.6) | 51.1(44.0, 58.2) |
| Rwanda | 2020 | SSA | L | 68.1(65.2, 71.0) | 85.1(82.5, 87.7) | 86.7(84.0, 89.4) | 87.0(84.1, 89.8) | 92.1(89.5, 94.7) | 24.0(20.1, 27.8) |
| Samoa | 2019 | EAP | L | 14.9(10.0, 19.8) | 29.7(23.2, 36.1) | 40.3(33.4, 47.1) | 60.0(54.2, 65.8) | 71.8(63.6, 79.9) | 56.9(47.6, 66.2) |
| STP | 2019 | SSA | LM | 57.6(50.6, 64.6) | 57.4(51.3, 63.4) | 65.9(60.1, 71.8) | 85.2(80.5, 90.0) | 92.3(88.2, 96.3) | 34.7(26.3, 43.1) |
| Senegal | 2019 | SSA | L | 63.8(58.3, 69.3) | 66.3(61.0, 71.6) | 67.2(62.0, 72.4) | 73.5(67.0, 80.1) | 84.7(80.4, 89.1) | 20.9(14.1, 27.8) |
| Sierra Leone* | 2019 | SSA | L | 74.2(71.2, 77.3) | 78.8(76.1, 81.5) | 78.8(75.8, 81.7) | 68.7(65.8, 71.7) | 70.3(66.2, 74.4) | -3.9(-9.1, 1.2) |
| South Africa | 2016 | SSA | UM | 63.5(57.3, 69.8) | 76.5(71.7, 81.3) | 74.8(69.5, 80.1) | 72.0(65.1, 78.9) | 91.1(86.0, 96.2) | 27.6(19.5, 35.7) |
| Suriname | 2018 | LAC | UM | 47.5(43.0, 51.9) | 69.1(64.6, 73.6) | 80.1(75.1, 85.2) | 88.2(83.8, 92.6) | 92.9(88.6, 97.2) | 45.4(39.3, 51.6) |
| Tajikistan | 2017 | ECA | LM | 53.8(48.8, 58.8) | 53.9(48.5, 59.2) | 61.0(56.0, 66.1) | 64.3(58.8, 69.7) | 72.3(68.2, 76.4) | 18.5(12.2, 24.9) |
| Timor-Leste | 2016 | EAP | LM | 52.1(47.5, 56.7) | 70.6(66.9, 74.2) | 79.1(75.5, 82.6) | 80.0(76.3, 83.6) | 83.5(79.8, 87.2) | 31.4(25.4, 37.4) |
| Togo | 2017 | SSA | L | 65.9(61.7, 70.0) | 70.6(65.9, 75.3) | 56.5(51.1, 61.9) | 53.2(47.3, 59.0) | 77.9(73.1, 82.6) | 12.0(5.7, 18.3) |
| Tonga | 2019 | EAP | LM | 53.6(45.9, 61.3) | 74.9(66.0, 83.9) | 79.4(69.8, 89.0) | 86.7(80.3, 93.1) | 86.4(78.3, 94.5) | 32.8(21.7, 43.9) |
| Tunisia | 2018 | MENA | LM | 69.4(64.9, 73.9) | 74.2(70.0, 78.5) | 78.6(74.0, 83.3) | 87.6(84.3, 91.0) | 92.2(88.8, 95.6) | 22.8(17.1, 28.5) |
| Tuvalu | 2019 | EAP | UM | 27.8(16.7, 38.8) | 36.0(21.5, 50.4) | 29.3(18.5, 40.1) | 60.7(47.0, 74.3) | 69.2(48.2, 90.1) | 41.4(16.9, 65.9) |
| Uganda | 2016 | SSA | L | 34.7(32.2, 37.2) | 49.0(46.1, 52.0) | 59.6(56.5, 62.7) | 64.0(60.7, 67.2) | 67.0(63.8, 70.3) | 32.3(28.2, 36.4) |
| Zambia | 2018 | SSA | LM | 47.8(44.9, 50.8) | 53.9(50.4, 57.3) | 61.0(57.4, 64.5) | 62.7(57.3, 68.0) | 66.8(61.1, 72.4) | 19.0(12.6, 25.4) |
| Zimbabwe | 2019 | SSA | L | 54.8(51.2, 58.4) | 70.7(67.1, 74.3) | 77.6(74.4, 80.8) | 67.9(64.1, 71.6) | 85.0(81.7, 88.3) | 30.2(25.3, 35.0) |

**Note:**

1. World Bank region group: “EAP” represents “East Asia and Pacific”; “ECA” represents “Europe and Central Asia”; “LAC” represents “Latin America and the Caribbean”; “MENA” represents “Middle East and North Africa”; “SA” represents “South Asia”; “SSA” represents “Sub-Saharan Africa”.

2. Income group: “L” represents “Low-income country”; “LM” represents “Lower-middle income country”; “UM” represents “Upper-middle income country”.

*. The disparity in prevalence between young children living in the wealthiest and poorest quintiles is not statistically significant at the 0.05 level.

**Table S10.** National prevalence of young children living in households with basic hygiene conditions and associated disparities by place of residence

| Country | Year | WB region^1^ | Income group^2^ | National (%) | Rural (%) | Urban (%) | Difference  (urban-rural) |
| --- | --- | --- | --- | --- | --- | --- | --- |
| Algeria | 2019 | MENA | UM | 83.2(80.8, 85.6) | 76.0(71.1, 80.9) | 88.5(86.9, 90.0) | 12.5(7.4, 17.6) |
| Angola | 2016 | SSA | UM | 24.0(21.8, 26.3) | 13.5(10.9, 16.1) | 31.1(27.9, 34.4) | 17.6(13.4, 21.9) |
| Armenia | 2016 | ECA | LM | 96.5(95.1, 97.9) | 93.2(90.2, 96.2) | 98.9(98.1, 99.8) | 5.7(2.6, 8.8) |
| Bangladesh | 2019 | SA | LM | 74.1(73.2, 75.0) | 70.6(69.6, 71.7) | 87.0(85.6, 88.5) | 16.4(14.6, 18.2) |
| Benin | 2018 | SSA | L | 8.9(7.8, 10.0) | 7.4(6.0, 8.7) | 11.4(9.4, 13.4) | 4.0(1.6, 6.4) |
| Burundi | 2017 | SSA | L | 4.7(4.0, 5.4) | 3.7(3.0, 4.3) | 14.7(11.0, 18.4) | 11.0(7.2, 14.8) |
| CAR | 2018 | SSA | L | 17.6(15.9, 19.4) | 12.0(10.1, 14.0) | 31.1(27.2, 34.9) | 19.0(14.7, 23.4) |
| Cameroon | 2018 | SSA | LM | 31.8(29.7, 34.0) | 21.5(18.7, 24.2) | 44.7(41.4, 48.0) | 23.2(18.9, 27.6) |
| Chad | 2019 | SSA | L | 36.1(34.1, 38.1) | 33.5(31.2, 35.7) | 50.1(46.6, 53.6) | 16.7(12.5, 20.8) |
| Congo DR | 2018 | SSA | L | 19.9(15.6, 24.1) | 14.7(8.2, 21.2) | 28.1(23.2, 33.0) | 13.4(5.2, 21.6) |
| Cote d’Ivoire | 2016 | SSA | LM | 22.9(20.4, 25.3) | 16.3(14.0, 18.6) | 33.1(28.4, 37.8) | 16.9(11.6, 22.1) |
| Dominican Republic | 2019 | LAC | UM | 55.9(53.7, 58.1) | 43.4(40.1, 46.8) | 60.5(57.8, 63.2) | 17.1(12.7, 21.4) |
| Ethiopia | 2016 | SSA | L | 6.0(5.0, 7.0) | 4.0(3.0, 5.0) | 21.9(17.6, 26.2) | 17.9(13.4, 22.3) |
| Gambia* | 2020 | SSA | L | 7.2(5.7, 8.6) | 5.8(3.5, 8.1) | 7.9(6.0, 9.8) | 2.1(-0.9, 5.1) |
| Ghana | 2017 | SSA | LM | 47.2(44.2, 50.2) | 40.8(37.0, 44.5) | 55.6(51.2, 60.1) | 14.8(9.0, 20.7) |
| Guinea | 2018 | SSA | L | 17.9(16.0, 19.9) | 12.6(10.6, 14.5) | 31.6(27.4, 35.7) | 19.0(14.4, 23.7) |
| Guinea-Bissau | 2019 | SSA | L | 15.6(14.0, 17.3) | 13.7(11.9, 15.6) | 21.0(17.6, 24.5) | 7.3(3.4, 11.3) |
| Guyana* | 2019 | LAC | UM | 85.0(81.9, 88.0) | 85.6(82.0, 89.1) | 83.0(77.8, 88.2) | -2.6(-8.9, 3.7) |
| Haiti | 2017 | LAC | L | 19.2(16.9, 21.4) | 15.0(12.4, 17.6) | 26.8(22.8, 30.9) | 11.8(7.0, 16.6) |
| India | 2016 | SA | LM | 55.1(54.7, 55.6) | 46.4(45.9, 46.9) | 77.5(76.6, 78.4) | 31.1(30.0, 32.1) |
| Indonesia | 2017 | EAP | LM | 88.3(87.4, 89.3) | 83.4(81.9, 85.0) | 93.5(92.6, 94.4) | 10.0(8.2, 11.8) |
| Iraq | 2018 | MENA | UM | 96.7(96.0, 97.4) | 95.0(93.6, 96.5) | 97.5(96.6, 98.4) | 2.5(0.8, 4.2) |
| Kiribati* | 2018 | EAP | LM | 59.7(56.1, 63.4) | 56.8(52.4, 61.3) | 62.3(56.7, 67.9) | 5.5(-1.7, 12.7) |
| Kyrgyzstan | 2018 | ECA | LM | 98.0(97.3, 98.8) | 97.5(96.4, 98.6) | 99.2(98.4, 99.9) | 1.6(0.3, 3.0) |
| Lao | 2017 | EAP | LM | 50.4(48.5, 52.3) | 42.3(40.0, 44.5) | 72.1(69.0, 75.2) | 29.8(26.0, 33.6) |
| Lesotho | 2018 | SSA | LM | 15.7(13.5, 17.9) | 10.2(8.0, 12.4) | 26.5(21.6, 31.3) | 16.2(10.9, 21.6) |
| Liberia | 2019 | SSA | L | 3.2(1.9, 4.4) | 0.9(0.3, 1.5) | 5.0(2.8, 7.2) | 4.1(1.8, 6.4) |
| Madagascar | 2018 | SSA | L | 19.2(17.7, 20.6) | 15.4(13.8, 16.9) | 34.1(30.6, 37.6) | 18.7(14.8, 22.5) |
| Malawi | 2020 | SSA | L | 27.1(25.6, 28.6) | 24.0(22.6, 25.4) | 48.1(42.4, 53.7) | 24.1(18.2, 29.9) |
| Maldives* | 2017 | SA | UM | 96.5(95.4, 97.6) | 96.0(95.1, 96.9) | 97.4(94.6, 100.2) | 1.4(-1.5, 4.3) |
| Mali | 2018 | SSA | L | 12.8(11.2, 14.4) | 8.9(7.4, 10.3) | 27.6(22.7, 32.4) | 18.7(13.7, 23.8) |
| Mongolia | 2018 | EAP | LM | 81.9(79.7, 84.1) | 73.5(70.0, 77.1) | 86.1(83.4, 88.8) | 12.6(8.1, 17.1) |
| Myanmar | 2016 | EAP | LM | 74.6(71.9, 77.3) | 70.1(66.9, 73.3) | 90.0(85.9, 94.0) | 19.8(14.7, 25.0) |
| Nepal | 2019 | SA | L | 77.8(75.7, 80.0) | 68.1(64.8, 71.5) | 83.1(80.5, 85.7) | 15.0(10.7, 19.2) |
| Nigeria | 2018 | SSA | LM | 29.0(27.4, 30.7) | 21.4(19.4, 23.4) | 40.8(38.0, 43.5) | 19.4(16.0, 22.8) |
| PNG | 2016-18 | SA | LM | 31.4(27.7, 35.0) | 27.5(23.3, 31.6) | 61.7(55.7, 67.7) | 34.3(27.0, 41.6) |
| Pakistan | 2018 | EAP | LM | 63.3(59.7, 66.9) | 53.2(48.5, 57.9) | 84.8(82.0, 87.6) | 31.6(26.1, 37.1) |
| Palestine* | 2019 | MENA | LM | 96.0(95.2, 96.8) | 95.3(93.8, 96.7) | 96.2(95.2, 97.1) | 0.9(-0.8, 2.6) |
| Paraguay | 2016 | LAC | UM | 88.0(86.1, 89.8) | 82.6(79.7, 85.6) | 91.8(89.5, 94.1) | 9.1(5.4, 12.9) |
| Philippines | 2017 | EAP | LM | 84.8(83.3, 86.3) | 80.8(78.7, 82.8) | 89.9(87.7, 92.0) | 9.1(6.1, 12.1) |
| Rwanda | 2020 | SSA | L | 23.5(21.6, 25.3) | 21.4(19.5, 23.3) | 33.3(27.7, 39.0) | 11.9(6.0, 17.9) |
| Samoa | 2019 | EAP | L | 75.6(71.6, 79.7) | 73.6(68.9, 78.3) | 86.1(81.9, 90.2) | 12.5(6.2, 18.8) |
| STP* | 2019 | SSA | LM | 51.4(47.3, 55.6) | 46.4(40.0, 52.8) | 54.0(48.8, 59.2) | 7.6(-0.7, 15.9) |
| Senegal | 2019 | SSA | L | 13.8(10.6, 16.9) | 8.8(7.3, 10.4) | 22.9(14.2, 31.6) | 14.0(5.2, 22.9) |
| Sierra Leone* | 2019 | SSA | L | 20.9(18.5, 23.2) | 20.4(17.3, 23.4) | 22.0(18.6, 25.3) | 1.6(-3.0, 6.1) |
| South Africa | 2016 | SSA | UM | 40.2(37.1, 43.3) | 27.1(23.6, 30.6) | 47.2(42.9, 51.6) | 20.1(14.5, 25.7) |
| Suriname* | 2018 | LAC | UM | 78.0(75.6, 80.4) | 76.3(72.7, 79.8) | 79.0(75.7, 82.2) | 2.7(-2.2, 7.5) |
| Tajikistan | 2017 | ECA | LM | 72.2(69.3, 75.1) | 68.7(65.2, 72.2) | 85.3(81.1, 89.5) | 16.6(11.1, 22.1) |
| Timor-Leste | 2016 | EAP | LM | 27.3(24.7, 29.8) | 21.4(18.6, 24.2) | 41.4(35.3, 47.5) | 20.0(13.2, 26.7) |
| Togo | 2017 | SSA | L | 19.2(17.0, 21.4) | 12.0(9.9, 14.0) | 30.8(26.6, 35.0) | 18.9(14.2, 23.5) |
| Tonga* | 2019 | EAP | LM | 77.7(73.5, 81.9) | 76.1(71.1, 81.1) | 83.3(76.2, 90.4) | 7.2(-1.5, 16.0) |
| Tunisia | 2018 | MENA | LM | 94.8(93.7, 95.9) | 89.3(86.9, 91.8) | 97.9(97.0, 98.7) | 8.5(5.9, 11.1) |
| Tuvalu | 2019 | EAP | UM | 95.3(92.6, 98.0) | 98.5(96.8, 100.1) | 93.7(89.7, 97.7) | -4.7(-9.2, -0.3) |
| Uganda | 2016 | SSA | L | 28.2(26.3, 30.1) | 23.8(21.8, 25.9) | 43.2(38.9, 47.6) | 19.4(14.6, 24.2) |
| Zambia | 2018 | SSA | LM | 20.3(17.9, 22.7) | 13.7(11.8, 15.5) | 32.6(26.7, 38.5) | 19.0(12.8, 25.1) |
| Zimbabwe | 2019 | SSA | L | 63.6(61.2, 66.0) | 59.4(56.3, 62.5) | 73.7(70.3, 77.2) | 14.3(9.7, 19.0) |

**Note:**

1. World Bank region group: “EAP” represents “East Asia and Pacific”; “ECA” represents “Europe and Central Asia”; “LAC” represents “Latin America and the Caribbean”; “MENA” represents “Middle East and North Africa”; “SA” represents “South Asia”; “SSA” represents “Sub-Saharan Africa”.

2. Income group: “L” represents “Low-income country”; “LM” represents “Lower-middle income country”; “UM” represents “Upper-middle income country”.

*. The disparity in prevalence between young children in urban and rural areas is not statistically significant at the 0.05 level.

**Table S11.** National prevalence of young children living in households with basic hygiene conditions and associated disparities by household wealth quintile

| **Country** | **Year** | **WB region^1^** | **Income group^2^** | **Wealth quintile** | | | | | **Difference (richest-poorest)** |
| --- | --- | --- | --- | --- | --- | --- | --- | --- | --- |
|  |  |  |  | **Poorest (%)** | **2 (%)** | **3 (%)** | **4 (%)** | **Richest (%)** |  |
| Algeria | 2019 | MENA | UM | 62.1(57.0, 67.3) | 80.6(77.1, 84.1) | 90.0(87.5, 92.5) | 95.5(94.3, 96.7) | 97.0(95.3, 98.8) | 34.9(29.4, 40.4) |
| Angola | 2016 | SSA | UM | 8.6(6.2, 10.9) | 17.0(13.8, 20.2) | 22.1(18.8, 25.4) | 31.8(27.1, 36.6) | 54.3(47.9, 60.7) | 45.7(38.9, 52.6) |
| Armenia | 2016 | ECA | LM | 88.9(83.8, 94.0) | 95.4(92.0, 98.7) | 97.6(95.3, 99.9) | 100.0(100.0, 100.0) | 100.0(100.0, 100.0) | 11.1(6.0, 16.2) |
| Bangladesh | 2019 | SA | LM | 43.9(41.9, 45.9) | 66.2(64.5, 68.0) | 77.8(76.2, 79.4) | 88.1(86.9, 89.4) | 96.7(96.1, 97.4) | 52.8(50.7, 55.0) |
| Benin | 2018 | SSA | L | 3.7(2.2, 5.2) | 5.3(3.7, 6.8) | 6.9(5.1, 8.7) | 8.6(6.8, 10.5) | 21.9(18.4, 25.4) | 18.2(14.4, 22.1) |
| Burundi | 2017 | SSA | L | 1.0(0.5, 1.5) | 3.2(2.0, 4.3) | 3.1(2.1, 4.1) | 5.0(3.4, 6.6) | 13.0(10.7, 15.4) | 12.0(9.6, 14.4) |
| CAR | 2018 | SSA | L | 4.7(2.8, 6.6) | 9.7(7.1, 12.2) | 13.9(11.1, 16.7) | 27.0(23.3, 30.7) | 43.4(39.1, 47.8) | 38.8(34.2, 43.3) |
| Cameroon | 2018 | SSA | LM | 8.8(6.6, 11.1) | 24.0(19.7, 28.4) | 35.0(31.5, 38.6) | 39.9(35.6, 44.1) | 63.3(59.5, 67.1) | 54.5(50.1, 58.9) |
| Chad | 2019 | SSA | L | 26.5(23.4, 29.6) | 30.1(27.2, 33.0) | 35.1(31.8, 38.4) | 40.3(36.2, 44.4) | 52.8(49.0, 56.6) | 26.3(21.4, 31.2) |
| Congo DR | 2018 | SSA | L | 7.4(0.9, 13.9) | 11.5(6.7, 16.3) | 21.6(11.3, 32.0) | 25.2(20.1, 30.3) | 41.6(33.3, 49.8) | 34.2(23.6, 44.7) |
| Cote d’Ivoire | 2016 | SSA | LM | 11.4(8.7, 14.2) | 17.8(14.6, 20.9) | 19.9(15.7, 24.0) | 23.3(19.6, 27.0) | 53.2(45.9, 60.6) | 41.8(34.0, 49.6) |
| Dominican Republic | 2019 | LAC | UM | 23.9(21.1, 26.8) | 41.9(37.8, 46.0) | 62.1(58.1, 66.0) | 82.0(78.1, 85.9) | 91.6(88.1, 95.2) | 67.7(63.2, 72.2) |
| Ethiopia | 2016 | SSA | L | 1.3(0.6, 2.1) | 3.1(1.4, 4.8) | 4.4(2.2, 6.5) | 6.8(4.6, 9.1) | 19.6(16.2, 23.0) | 18.3(14.7, 21.8) |
| Gambia | 2020 | SSA | L | 4.3(2.6, 6.1) | 3.9(1.8, 6.0) | 4.4(1.6, 7.3) | 7.1(4.2, 9.9) | 18.4(13.9, 23.0) | 14.1(9.2, 19.0) |
| Ghana | 2017 | SSA | LM | 35.0(30.2, 39.9) | 41.0(35.1, 46.9) | 45.0(40.1, 49.9) | 52.2(46.7, 57.6) | 66.0(61.4, 70.5) | 30.9(24.5, 37.4) |
| Guinea | 2018 | SSA | L | 11.5(8.5, 14.4) | 10.1(7.3, 12.8) | 14.5(11.0, 17.9) | 20.9(17.1, 24.8) | 41.1(35.5, 46.8) | 29.7(23.3, 36.1) |
| Guinea-Bissau | 2019 | SSA | L | 7.4(4.8, 9.9) | 14.2(10.9, 17.5) | 20.5(16.0, 25.0) | 16.2(12.3, 20.1) | 22.8(17.8, 27.7) | 15.4(9.8, 21.0) |
| Guyana | 2019 | LAC | UM | 69.7(64.2, 75.2) | 88.5(83.3, 93.7) | 86.9(77.4, 96.4) | 94.1(90.6, 97.7) | 95.7(92.6, 98.8) | 26.0(19.7, 32.4) |
| Haiti | 2017 | LAC | L | 10.2(7.6, 12.7) | 14.5(11.7, 17.4) | 17.2(13.6, 20.8) | 25.0(19.8, 30.1) | 37.2(31.9, 42.4) | 27.0(21.2, 32.9) |
| India | 2016 | SA | LM | 24.0(23.4, 24.7) | 43.0(42.2, 43.8) | 59.9(59.1, 60.8) | 77.7(76.8, 78.6) | 92.5(92.0, 93.1) | 68.5(67.7, 69.4) |
| Indonesia | 2017 | EAP | LM | 68.1(65.3, 70.9) | 85.6(83.8, 87.3) | 93.6(92.5, 94.8) | 96.3(95.5, 97.2) | 98.8(98.4, 99.3) | 30.7(27.9, 33.6) |
| Iraq | 2018 | MENA | UM | 89.8(87.5, 92.0) | 97.5(96.4, 98.6) | 98.3(97.0, 99.6) | 99.6(99.2, 100.0) | 99.8(99.6, 100.0) | 10.0(7.8, 12.3) |
| Kiribati | 2018 | EAP | LM | 45.4(39.0, 51.8) | 62.8(57.1, 68.4) | 58.1(50.3, 65.9) | 57.8(50.0, 65.6) | 77.7(69.9, 85.6) | 32.3(22.1, 42.6) |
| Kyrgyzstan | 2018 | ECA | LM | 98.4(97.1, 99.7) | 96.4(94.3, 98.5) | 98.4(96.5, 100.3) | 97.7(95.9, 99.6) | 100.0(100.0, 100.0) | 1.6(0.3, 2.9) |
| Lao | 2017 | EAP | LM | 20.3(17.3, 23.3) | 38.4(35.4, 41.4) | 54.4(51.1, 57.8) | 68.9(65.8, 72.0) | 88.0(85.8, 90.1) | 67.7(64.0, 71.3) |
| Lesotho | 2018 | SSA | LM | 4.5(2.3, 6.8) | 6.7(4.1, 9.2) | 10.4(6.4, 14.4) | 19.3(14.0, 24.6) | 44.6(36.7, 52.4) | 40.0(31.8, 48.2) |
| Liberia | 2019 | SSA | L | 0.6(0.0, 1.1) | 1.2(0.1, 2.3) | 1.3(-0.2, 2.9) | 2.7(0.0, 5.4) | 10.7(5.9, 15.5) | 10.2(5.3, 15.0) |
| Madagascar | 2018 | SSA | L | 3.7(2.8, 4.6) | 9.4(7.7, 11.1) | 20.5(17.5, 23.5) | 29.7(26.3, 33.1) | 47.7(43.8, 51.6) | 44.0(40.0, 48.0) |
| Malawi | 2020 | SSA | L | 14.5(12.7, 16.3) | 22.0(19.3, 24.7) | 25.3(22.9, 27.6) | 32.7(29.6, 35.7) | 50.6(46.7, 54.6) | 36.1(31.7, 40.5) |
| Maldives | 2017 | SA | UM | 92.3(90.1, 94.5) | 96.5(94.9, 98.2) | 98.4(97.3, 99.5) | 95.9(91.1, 100.7) | 99.3(97.8, 100.7) | 6.9(4.3, 9.6) |
| Mali | 2018 | SSA | L | 3.5(2.0, 4.9) | 5.7(4.0, 7.3) | 8.9(6.5, 11.2) | 16.3(13.1, 19.4) | 32.2(27.5, 36.8) | 28.7(23.8, 33.6) |
| Mongolia | 2018 | EAP | LM | 63.6(58.8, 68.3) | 71.6(66.7, 76.4) | 82.5(78.7, 86.3) | 92.3(88.7, 96.0) | 99.3(98.4, 100.1) | 35.7(30.9, 40.5) |
| Myanmar | 2016 | EAP | LM | 54.6(49.5, 59.8) | 70.2(65.8, 74.7) | 83.5(79.9, 87.2) | 88.1(84.0, 92.2) | 95.9(93.8, 98.0) | 41.3(35.7, 46.9) |
| Nepal | 2019 | SA | L | 49.0(44.9, 53.0) | 75.1(70.8, 79.4) | 83.1(79.6, 86.5) | 91.8(89.3, 94.3) | 98.9(98.0, 99.9) | 50.0(45.7, 54.2) |
| Nigeria | 2018 | SSA | LM | 9.9(7.1, 12.7) | 21.2(17.5, 24.8) | 23.6(20.5, 26.6) | 37.0(33.7, 40.3) | 61.0(57.9, 64.2) | 51.1(46.8, 55.4) |
| PNG | 2016-18 | SA | LM | 8.4(5.7, 11.1) | 14.7(11.1, 18.2) | 23.2(19.1, 27.3) | 38.9(34.9, 42.9) | 73.2(65.9, 80.5) | 64.8(57.1, 72.5) |
| Pakistan | 2018 | EAP | LM | 18.9(15.2, 22.6) | 48.8(43.5, 54.1) | 73.3(69.1, 77.4) | 89.0(86.4, 91.7) | 97.7(96.5, 98.9) | 78.8(75.0, 82.7) |
| Palestine | 2019 | MENA | LM | 91.4(89.3, 93.6) | 96.6(95.0, 98.2) | 95.5(93.4, 97.6) | 97.7(96.5, 98.9) | 99.6(99.2, 100.0) | 8.2(6.0, 10.4) |
| Paraguay | 2016 | LAC | UM | 72.2(67.8, 76.7) | 87.1(83.3, 90.9) | 95.1(93.1, 97.1) | 95.7(93.5, 98.0) | 97.6(96.0, 99.2) | 25.4(20.6, 30.1) |
| Philippines | 2017 | EAP | LM | 70.1(67.3, 72.9) | 82.9(80.1, 85.6) | 89.5(87.0, 92.0) | 96.3(94.9, 97.7) | 97.4(95.1, 99.7) | 27.2(23.6, 30.9) |
| Rwanda | 2020 | SSA | L | 12.8(10.6, 14.9) | 18.3(15.6, 20.9) | 21.8(18.9, 24.7) | 25.8(21.6, 30.0) | 42.1(37.0, 47.2) | 29.3(23.7, 35.0) |
| Samoa | 2019 | EAP | L | 52.4(43.8, 61.0) | 73.0(66.2, 79.7) | 80.8(73.8, 87.7) | 90.2(86.7, 93.7) | 91.0(86.3, 95.6) | 38.6(29.2, 47.9) |
| STP | 2019 | SSA | LM | 43.0(35.8, 50.2) | 39.2(31.2, 47.1) | 46.7(40.3, 53.0) | 57.2(49.5, 64.9) | 79.4(72.3, 86.5) | 36.4(27.7, 45.0) |
| Senegal | 2019 | SSA | L | 9.4(6.4, 12.5) | 9.6(6.6, 12.7) | 7.5(5.2, 9.9) | 8.9(3.4, 14.4) | 38.0(27.7, 48.3) | 28.6(17.7, 39.4) |
| Sierra Leone | 2019 | SSA | L | 18.4(14.0, 22.9) | 19.2(15.0, 23.3) | 20.3(16.6, 24.0) | 21.0(16.9, 25.0) | 29.2(23.0, 35.4) | 10.8(3.1, 18.4) |
| South Africa | 2016 | SSA | UM | 15.4(11.5, 19.4) | 25.2(20.5, 30.0) | 36.9(31.3, 42.5) | 57.5(50.9, 64.0) | 83.7(78.1, 89.3) | 68.2(61.3, 75.1) |
| Suriname | 2018 | LAC | UM | 64.8(60.2, 69.5) | 78.3(73.4, 83.1) | 83.7(78.8, 88.6) | 89.1(85.1, 93.1) | 90.7(85.6, 95.9) | 25.9(18.8, 33.0) |
| Tajikistan | 2017 | ECA | LM | 51.8(44.4, 59.2) | 67.6(62.1, 73.1) | 73.3(68.7, 78.0) | 78.5(74.3, 82.7) | 90.2(87.2, 93.3) | 38.5(30.5, 46.4) |
| Timor-Leste | 2016 | EAP | LM | 11.2(8.3, 14.1) | 16.1(13.0, 19.3) | 24.0(20.0, 28.0) | 30.3(24.8, 35.7) | 54.2(48.3, 60.2) | 43.0(36.4, 49.6) |
| Togo | 2017 | SSA | L | 4.8(2.9, 6.7) | 11.6(8.3, 15.0) | 17.1(13.0, 21.1) | 24.0(19.9, 28.1) | 43.2(37.4, 49.1) | 38.4(32.2, 44.6) |
| Tonga | 2019 | EAP | LM | 54.9(46.5, 63.2) | 74.8(66.2, 83.5) | 82.5(74.0, 91.0) | 88.0(80.4, 95.5) | 95.2(90.9, 99.5) | 40.3(30.9, 49.7) |
| Tunisia | 2018 | MENA | LM | 85.7(82.2, 89.2) | 91.5(88.8, 94.2) | 98.2(96.9, 99.6) | 99.3(98.5, 100.1) | 99.4(98.7, 100.1) | 13.7(10.1, 17.3) |
| Tuvalu | 2019 | EAP | UM | 85.9(75.8, 96.0) | 97.7(93.3, 102.2) | 96.9(90.9, 102.9) | 97.9(94.0, 101.9) | 98.7(96.4, 101.1) | 12.8(2.1, 23.5) |
| Uganda | 2016 | SSA | L | 13.6(11.1, 16.1) | 20.1(17.4, 22.8) | 25.7(22.4, 28.9) | 31.5(28.1, 34.9) | 50.1(45.7, 54.4) | 36.5(31.5, 41.6) |
| Zambia | 2018 | SSA | LM | 12.2(9.5, 14.9) | 12.5(9.6, 15.4) | 14.3(11.7, 16.9) | 23.6(17.1, 30.0) | 47.5(41.0, 54.0) | 35.3(28.2, 42.4) |
| Zimbabwe | 2019 | SSA | L | 48.6(43.2, 54.0) | 60.4(56.3, 64.5) | 65.4(61.1, 69.8) | 72.4(68.7, 76.0) | 76.4(72.6, 80.3) | 27.9(21.2, 34.5) |

**Note:**

1. World Bank region group: “EAP” represents “East Asia and Pacific”; “ECA” represents “Europe and Central Asia”; “LAC” represents “Latin America and the Caribbean”; “MENA” represents “Middle East and North Africa”; “SA” represents “South Asia”; “SSA” represents “Sub-Saharan Africa”.

2. Income group: “L” represents “Low-income country”; “LM” represents “Lower-middle income country”; “UM” represents “Upper-middle income country”.

**Table S12.** National prevalence of young children living in households with basic sanitation conditions and associated disparities by place of residence

| Country | Year | WB region^1^ | Income group^2^ | National (%) | Rural (%) | Urban (%) | Difference  (urban-rural) |
| --- | --- | --- | --- | --- | --- | --- | --- |
| Algeria | 2019 | MENA | UM | 81.9(80.3, 83.4) | 76.3(73.3, 79.3) | 86.0(84.5, 87.4) | 9.7(6.3, 13.0) |
| Angola | 2016 | SSA | UM | 39.7(37.1, 42.3) | 20.8(17.4, 24.2) | 52.1(48.6, 55.6) | 31.3(26.4, 36.2) |
| Armenia | 2016 | ECA | LM | 78.4(75.2, 81.6) | 54.1(48.2, 59.9) | 96.4(94.5, 98.3) | 42.3(36.1, 48.5) |
| Bangladesh* | 2019 | SA | LM | 58.9(57.9, 59.9) | 58.6(57.5, 59.6) | 60.3(57.8, 62.9) | 1.8(-1.0, 4.5) |
| Benin | 2018 | SSA | L | 10.4(9.1, 11.7) | 5.7(4.4, 7.1) | 17.9(15.3, 20.5) | 12.2(9.2, 15.1) |
| Burundi* | 2017 | SSA | L | 41.2(39.4, 43.0) | 41.3(39.4, 43.1) | 40.3(33.7, 46.9) | -0.9(-7.8, 6.0) |
| CAR | 2018 | SSA | L | 8.2(6.9, 9.6) | 5.0(3.5, 6.6) | 15.8(13.0, 18.6) | 10.8(7.6, 14.0) |
| Cameroon | 2018 | SSA | LM | 37.7(34.4, 41.0) | 24.9(20.4, 29.4) | 53.5(49.8, 57.3) | 28.7(22.8, 34.5) |
| Chad | 2019 | SSA | L | 10.2(9.1, 11.4) | 4.9(4.0, 5.7) | 38.6(34.1, 43.1) | 33.8(29.2, 38.4) |
| Congo DR | 2018 | SSA | L | 11.5(9.4, 13.6) | 8.2(5.8, 10.7) | 16.8(12.9, 20.6) | 8.6(4.0, 13.1) |
| Cote d’Ivoire | 2016 | SSA | LM | 25.2(22.7, 27.8) | 13.3(10.9, 15.6) | 44.0(39.1, 48.8) | 30.7(25.3, 36.1) |
| Dominican Republic | 2019 | LAC | UM | 81.0(79.4, 82.6) | 70.6(66.7, 74.5) | 84.6(83.0, 86.2) | 13.9(9.7, 18.1) |
| Ethiopia | 2016 | SSA | L | 5.4(4.3, 6.4) | 3.9(2.9, 4.9) | 17.3(12.9, 21.6) | 13.4(8.9, 17.8) |
| Gambia | 2020 | SSA | L | 48.4(45.3, 51.6) | 30.0(25.6, 34.4) | 58.2(53.9, 62.4) | 28.1(22.0, 34.2) |
| Ghana | 2017 | SSA | LM | 17.9(15.8, 20.1) | 15.2(12.5, 18.0) | 21.5(18.1, 24.9) | 6.3(1.9, 10.7) |
| Guinea | 2018 | SSA | L | 22.8(20.6, 24.9) | 16.4(14.1, 18.6) | 38.8(35.2, 42.3) | 22.4(18.2, 26.6) |
| Guinea-Bissau | 2019 | SSA | L | 9.6(8.1, 11.0) | 4.0(2.7, 5.2) | 25.5(21.3, 29.7) | 21.5(17.1, 25.9) |
| Guyana* | 2019 | LAC | UM | 86.6(84.0, 89.3) | 86.7(83.6, 89.8) | 86.4(81.9, 91.0) | -0.3(-5.8, 5.2) |
| Haiti | 2017 | LAC | L | 26.7(24.3, 29.1) | 20.9(18.0, 23.7) | 37.5(33.1, 42.0) | 16.7(11.3, 22.0) |
| India | 2016 | SA | LM | 40.3(39.9, 40.8) | 30.9(30.4, 31.3) | 64.6(63.4, 65.7) | 33.7(32.4, 34.9) |
| Indonesia | 2017 | EAP | LM | 74.3(72.7, 75.8) | 67.4(65.2, 69.6) | 81.5(79.5, 83.6) | 14.1(11.1, 17.1) |
| Iraq | 2018 | MENA | UM | 91.0(89.7, 92.3) | 85.4(81.8, 89.0) | 93.7(92.6, 94.8) | 8.3(4.5, 12.1) |
| Kiribati | 2018 | EAP | LM | 42.3(37.8, 46.8) | 34.8(29.5, 40.1) | 49.0(42.1, 55.9) | 14.2(5.5, 23.0) |
| Kyrgyzstan | 2018 | ECA | LM | 98.1(97.3, 98.9) | 99.1(98.4, 99.8) | 95.9(94.0, 97.9) | -3.2(-5.2, -1.1) |
| Lao | 2017 | EAP | LM | 65.2(63.0, 67.4) | 56.3(53.5, 59.0) | 89.4(87.3, 91.4) | 33.1(29.6, 36.5) |
| Lesotho | 2018 | SSA | LM | 46.2(42.9, 49.6) | 49.4(45.1, 53.8) | 39.9(34.8, 45.0) | -9.5(-16.3, -2.8) |
| Liberia | 2019 | SSA | L | 19.4(16.5, 22.3) | 7.6(5.4, 9.8) | 29.8(24.9, 34.6) | 22.2(16.9, 27.5) |
| Madagascar | 2018 | SSA | L | 13.2(11.9, 14.5) | 12.5(11.0, 14.0) | 15.8(13.6, 18.0) | 3.3(0.7, 6.0) |
| Malawi* | 2020 | SSA | L | 40.7(38.9, 42.5) | 41.5(39.6, 43.3) | 35.6(29.3, 41.8) | -5.9(-12.4, 0.6) |
| Maldives* | 2017 | SA | UM | 98.0(97.1, 98.9) | 97.7(96.7, 98.7) | 98.6(96.9, 100.3) | 0.8(-1.1, 2.8) |
| Mali | 2018 | SSA | L | 29.0(26.7, 31.3) | 26.8(24.1, 29.4) | 37.7(33.3, 42.2) | 11.0(5.8, 16.1) |
| Mongolia | 2018 | EAP | LM | 68.2(65.1, 71.4) | 49.5(45.0, 53.9) | 77.7(73.7, 81.7) | 28.3(22.2, 34.3) |
| Myanmar | 2016 | EAP | LM | 42.2(39.4, 45.0) | 37.6(34.4, 40.7) | 58.5(52.5, 64.5) | 21.0(14.2, 27.8) |
| Nepal | 2019 | SA | L | 74.5(72.2, 76.7) | 79.6(76.6, 82.5) | 71.7(68.7, 74.7) | -7.8(-12.1, -3.6) |
| Nigeria | 2018 | SSA | LM | 31.5(29.8, 33.1) | 25.1(23.1, 27.2) | 41.5(38.8, 44.2) | 16.4(13.0, 19.8) |
| PNG | 2016-18 | SA | LM | 21.5(18.0, 25.0) | 18.5(14.6, 22.4) | 47.6(39.1, 56.0) | 29.1(19.7, 38.4) |
| Pakistan | 2018 | EAP | LM | 67.0(63.7, 70.4) | 57.7(53.2, 62.2) | 86.9(84.1, 89.8) | 29.2(23.9, 34.6) |
| Palestine* | 2019 | MENA | LM | 97.1(96.4, 97.8) | 96.7(95.3, 98.0) | 97.2(96.4, 98.0) | 0.5(-1.1, 2.1) |
| Paraguay | 2016 | LAC | UM | 78.0(75.5, 80.5) | 64.4(60.5, 68.3) | 87.2(84.0, 90.3) | 22.7(17.7, 27.7) |
| Philippines* | 2017 | EAP | LM | 68.2(66.1, 70.4) | 67.0(64.1, 69.8) | 69.8(66.3, 73.4) | 2.9(-1.8, 7.5) |
| Rwanda | 2020 | SSA | L | 56.2(54.4, 58.0) | 58.5(56.6, 60.5) | 45.1(40.7, 49.6) | -13.4(-18.3, -8.5) |
| Samoa* | 2019 | EAP | L | 95.4(93.9, 96.9) | 95.1(93.3, 96.8) | 97.2(95.6, 98.9) | 2.2(-0.2, 4.6) |
| STP* | 2019 | SSA | LM | 37.9(33.8, 42.1) | 34.6(29.3, 39.8) | 39.6(33.9, 45.4) | 5.0(-2.8, 12.8) |
| Senegal | 2019 | SSA | L | 54.9(50.3, 59.4) | 45.8(39.8, 51.7) | 70.7(65.1, 76.4) | 24.9(16.7, 33.2) |
| Sierra Leone | 2019 | SSA | L | 14.2(12.9, 15.6) | 7.2(5.8, 8.6) | 27.2(24.4, 30.0) | 19.9(16.8, 23.1) |
| South Africa | 2016 | SSA | UM | 54.9(51.2, 58.6) | 44.1(39.9, 48.4) | 60.9(55.6, 66.3) | 16.8(10.0, 23.6) |
| Suriname | 2018 | LAC | UM | 81.8(79.1, 84.4) | 66.4(61.1, 71.8) | 89.7(87.0, 92.5) | 23.3(17.3, 29.3) |
| Tajikistan* | 2017 | ECA | LM | 97.3(96.2, 98.3) | 97.8(96.8, 98.9) | 95.1(91.8, 98.3) | -2.8(-6.2, 0.6) |
| Timor-Leste | 2016 | EAP | LM | 51.8(49.0, 54.5) | 43.6(40.4, 46.7) | 72.1(67.4, 76.9) | 28.6(22.9, 34.2) |
| Togo | 2017 | SSA | L | 15.1(12.6, 17.6) | 5.7(3.9, 7.6) | 30.1(25.5, 34.8) | 24.4(19.4, 29.4) |
| Tonga | 2019 | EAP | LM | 91.7(89.4, 93.9) | 90.6(87.9, 93.3) | 95.8(93.1, 98.5) | 5.2(1.3, 9.1) |
| Tunisia | 2018 | MENA | LM | 96.1(95.1, 97.1) | 91.8(89.7, 94.0) | 98.5(97.7, 99.3) | 6.7(4.4, 9.0) |
| Tuvalu* | 2019 | EAP | UM | 83.4(78.2, 88.7) | 86.2(79.6, 92.9) | 82.0(74.8, 89.2) | -4.3(-14.3, 5.8) |
| Uganda | 2016 | SSA | L | 17.2(15.8, 18.6) | 15.1(13.6, 16.6) | 25.0(21.6, 28.5) | 10.0(6.2, 13.7) |
| Zambia | 2018 | SSA | LM | 28.1(25.7, 30.6) | 24.8(22.2, 27.5) | 34.2(29.0, 39.3) | 9.3(3.5, 15.1) |
| Zimbabwe | 2019 | SSA | L | 30.3(27.9, 32.6) | 28.4(25.6, 31.2) | 34.9(30.6, 39.1) | 6.5(1.4, 11.6) |

**Note:**

1. World Bank region group: “EAP” represents “East Asia and Pacific”; “ECA” represents “Europe and Central Asia”; “LAC” represents “Latin America and the Caribbean”; “MENA” represents “Middle East and North Africa”; “SA” represents “South Asia”; “SSA” represents “Sub-Saharan Africa”.

2. Income group: “L” represents “Low-income country”; “LM” represents “Lower-middle income country”; “UM” represents “Upper-middle income country”.

*. The disparity in prevalence between young children in urban and rural areas is not statistically significant at the 0.05 level.

**Table S13.** National prevalence of young children living in households with basic sanitation conditions and associated disparities by household wealth quintile

| **Country** | **Year** | **WB region^1^** | **Income group^2^** |  |  | **Wealth quintile** |  |  | **Difference (richest-poorest)** |
| --- | --- | --- | --- | --- | --- | --- | --- | --- | --- |
|  |  |  |  | **Poorest (%)** | **2 (%)** | **3 (%)** | **4 (%)** | **Richest (%)** |  |
| Algeria | 2019 | MENA | UM | 64.3(60.7, 67.9) | 78.6(75.8, 81.4) | 86.3(84.0, 88.5) | 93.4(91.8, 95.0) | 95.2(93.5, 96.9) | 30.9(26.9, 34.9) |
| Angola | 2016 | SSA | UM | 8.1(5.9, 10.4) | 30.0(26.3, 33.7) | 42.9(38.8, 47.1) | 53.2(46.9, 59.5) | 83.4(78.7, 88.1) | 75.2(70.0, 80.5) |
| Armenia | 2016 | ECA | LM | 38.6(29.9, 47.3) | 66.8(60.1, 73.5) | 87.5(83.0, 92.1) | 97.8(96.0, 99.6) | 98.6(97.1, 100.2) | 60.0(51.2, 68.9) |
| Bangladesh | 2019 | SA | LM | 39.8(37.9, 41.6) | 49.0(47.1, 50.8) | 59.9(58.1, 61.7) | 65.5(63.4, 67.6) | 81.5(79.5, 83.5) | 41.7(39.0, 44.5) |
| Benin | 2018 | SSA | L | 0.1(-0.1, 0.4) | 1.0(0.3, 1.6) | 2.9(1.5, 4.3) | 13.4(11.0, 15.8) | 38.9(35.0, 42.8) | 38.7(34.8, 42.7) |
| Burundi | 2017 | SSA | L | 17.3(14.9, 19.8) | 35.7(32.8, 38.7) | 45.9(42.7, 49.2) | 57.0(53.6, 60.5) | 56.5(51.9, 61.1) | 39.1(33.9, 44.3) |
| CAR | 2018 | SSA | L | 1.0(0.1, 1.8) | 3.6(2.2, 5.0) | 6.7(3.4, 10.1) | 10.2(7.2, 13.2) | 26.1(22.4, 29.8) | 25.1(21.6, 28.7) |
| Cameroon | 2018 | SSA | LM | 7.7(4.4, 11.1) | 28.5(22.4, 34.5) | 41.5(37.2, 45.8) | 47.2(42.6, 51.9) | 78.0(74.4, 81.7) | 70.3(65.3, 75.3) |
| Chad | 2019 | SSA | L | 0.4(0.2, 0.6) | 1.7(0.8, 2.6) | 3.3(2.4, 4.3) | 10.2(8.0, 12.3) | 42.9(38.9, 47.0) | 42.6(38.5, 46.6) |
| Congo DR | 2018 | SSA | L | 5.9(3.5, 8.2) | 8.9(6.3, 11.5) | 10.4(7.0, 13.8) | 11.0(8.1, 14.0) | 25.9(19.3, 32.5) | 20.0(13.0, 27.1) |
| Cote d’Ivoire | 2016 | SSA | LM | 4.3(2.8, 5.8) | 11.3(8.0, 14.6) | 20.0(16.3, 23.6) | 33.3(28.0, 38.7) | 81.8(77.0, 86.5) | 77.4(72.4, 82.4) |
| Dominican Republic | 2019 | LAC | UM | 41.8(38.4, 45.1) | 83.2(80.4, 85.9) | 95.2(93.4, 97.1) | 97.2(95.5, 98.8) | 99.4(98.8, 100.0) | 57.6(54.2, 61.1) |
| Ethiopia | 2016 | SSA | L | 2.0(0.9, 3.1) | 3.1(1.2, 5.1) | 4.9(3.2, 6.7) | 4.4(2.7, 6.1) | 16.3(12.8, 19.7) | 14.3(10.6, 17.9) |
| Gambia | 2020 | SSA | L | 17.0(13.5, 20.5) | 35.3(29.6, 41.0) | 44.9(37.3, 52.4) | 63.9(57.8, 70.0) | 94.5(91.3, 97.7) | 77.5(72.8, 82.3) |
| Ghana | 2017 | SSA | LM | 10.3(7.1, 13.4) | 6.7(4.7, 8.8) | 13.7(9.8, 17.6) | 16.3(12.3, 20.3) | 46.2(40.7, 51.7) | 35.9(29.5, 42.3) |
| Guinea | 2018 | SSA | L | 4.3(2.7, 5.8) | 11.3(8.4, 14.2) | 28.7(24.9, 32.4) | 30.8(26.5, 35.1) | 50.9(46.0, 55.7) | 46.6(41.5, 51.7) |
| Guinea-Bissau | 2019 | SSA | L | 0.5(0.1, 0.9) | 2.1(0.8, 3.4) | 3.0(1.7, 4.3) | 12.2(8.1, 16.3) | 44.6(37.9, 51.2) | 44.1(37.4, 50.8) |
| Guyana | 2019 | LAC | UM | 73.8(68.0, 79.7) | 83.2(77.1, 89.4) | 95.7(93.1, 98.3) | 91.9(86.3, 97.5) | 97.9(96.1, 99.7) | 24.1(18.0, 30.2) |
| Haiti | 2017 | LAC | L | 6.6(3.5, 9.8) | 19.0(15.3, 22.7) | 26.7(21.7, 31.8) | 34.2(30.1, 38.2) | 63.3(57.9, 68.8) | 56.7(50.4, 63.0) |
| India | 2016 | SA | LM | 4.8(4.5, 5.2) | 20.3(19.7, 21.0) | 41.0(40.2, 41.9) | 71.2(70.2, 72.2) | 92.4(91.8, 93.0) | 87.6(86.9, 88.3) |
| Indonesia | 2017 | EAP | LM | 36.9(34.2, 39.7) | 65.3(62.5, 68.0) | 81.1(78.8, 83.4) | 92.9(91.5, 94.3) | 97.3(96.4, 98.2) | 60.3(57.5, 63.2) |
| Iraq | 2018 | MENA | UM | 78.0(74.3, 81.6) | 90.2(88.0, 92.4) | 94.7(93.1, 96.3) | 96.8(95.4, 98.2) | 98.8(98.1, 99.6) | 20.8(17.1, 24.6) |
| Kiribati | 2018 | EAP | LM | 17.3(11.4, 23.2) | 39.3(32.7, 46.0) | 26.9(20.4, 33.5) | 55.9(46.8, 65.0) | 78.6(70.9, 86.4) | 61.3(51.5, 71.1) |
| Kyrgyzstan* | 2018 | ECA | LM | 98.0(96.5, 99.5) | 99.2(97.8, 100.7) | 98.0(96.2, 99.7) | 96.4(94.2, 98.7) | 98.8(97.4, 100.1) | 0.8(-1.3, 2.8) |
| Lao | 2017 | EAP | LM | 21.2(17.9, 24.5) | 53.6(50.3, 56.9) | 81.5(78.9, 84.0) | 94.5(93.1, 96.0) | 99.0(98.4, 99.5) | 77.8(74.4, 81.1) |
| Lesotho | 2018 | SSA | LM | 24.0(18.3, 29.6) | 46.1(40.4, 51.9) | 57.0(50.4, 63.6) | 52.4(46.2, 58.6) | 56.6(50.4, 62.8) | 32.6(24.3, 41.0) |
| Liberia | 2019 | SSA | L | 2.3(-0.6, 5.3) | 6.4(3.5, 9.4) | 18.3(14.7, 21.9) | 24.7(18.0, 31.4) | 57.2(49.1, 65.4) | 54.9(46.1, 63.7) |
| Madagascar | 2018 | SSA | L | 3.2(2.2, 4.2) | 10.2(8.2, 12.2) | 14.1(11.7, 16.4) | 21.0(17.6, 24.4) | 25.4(21.6, 29.1) | 22.2(18.3, 26.0) |
| Malawi | 2020 | SSA | L | 21.0(18.2, 23.9) | 38.8(35.4, 42.2) | 49.8(46.7, 52.9) | 50.8(47.4, 54.2) | 53.7(48.8, 58.6) | 32.7(27.0, 38.3) |
| Maldives* | 2017 | SA | UM | 96.6(94.7, 98.5) | 97.2(95.4, 99.0) | 98.4(97.1, 99.7) | 99.7(99.0, 100.3) | 98.6(95.8, 101.3) | 2.0(-1.4, 5.3) |
| Mali | 2018 | SSA | L | 11.3(8.1, 14.5) | 18.5(14.8, 22.2) | 32.0(28.0, 36.0) | 40.6(36.0, 45.1) | 47.5(43.0, 51.9) | 36.2(30.7, 41.6) |
| Mongolia | 2018 | EAP | LM | 30.7(26.4, 35.0) | 58.2(53.7, 62.8) | 62.4(56.4, 68.5) | 90.7(87.6, 93.7) | 99.6(98.9, 100.2) | 68.8(64.4, 73.2) |
| Myanmar | 2016 | EAP | LM | 18.3(14.7, 21.9) | 33.2(28.7, 37.7) | 46.5(40.9, 52.0) | 58.4(53.4, 63.3) | 82.7(78.2, 87.2) | 64.4(58.6, 70.3) |
| Nepal | 2019 | SA | L | 77.7(74.3, 81.2) | 76.7(72.7, 80.8) | 78.3(74.6, 82.1) | 72.8(68.6, 77.0) | 64.3(57.6, 71.0) | -13.4(-21.0, -5.9) |
| Nigeria | 2018 | SSA | LM | 10.2(8.4, 12.1) | 23.0(19.9, 26.0) | 32.8(29.9, 35.6) | 40.1(36.9, 43.2) | 60.9(57.7, 64.1) | 50.7(47.0, 54.4) |
| PNG | 2016-18 | SA | LM | 3.8(2.2, 5.5) | 13.2(9.8, 16.6) | 14.9(11.1, 18.6) | 19.6(16.4, 22.8) | 62.5(53.2, 71.7) | 58.6(49.2, 68.0) |
| Pakistan | 2018 | EAP | LM | 23.2(18.2, 28.1) | 57.9(52.5, 63.3) | 78.9(74.8, 82.9) | 87.7(84.5, 90.9) | 98.0(97.0, 99.0) | 74.9(69.8, 79.9) |
| Palestine | 2019 | MENA | LM | 94.9(93.1, 96.7) | 95.7(93.1, 98.3) | 98.3(97.4, 99.1) | 97.7(96.4, 99.0) | 99.3(98.6, 100.0) | 4.4(2.5, 6.4) |
| Paraguay | 2016 | LAC | UM | 36.6(31.7, 41.6) | 79.5(74.9, 84.2) | 93.9(91.1, 96.8) | 97.2(94.5, 100.0) | 99.7(99.3, 100.1) | 63.0(58.1, 67.9) |
| Philippines | 2017 | EAP | LM | 40.4(36.7, 44.1) | 60.1(56.2, 64.1) | 75.1(70.9, 79.2) | 93.4(91.2, 95.6) | 99.0(98.1, 99.9) | 58.6(54.7, 62.4) |
| Rwanda | 2020 | SSA | L | 34.4(31.3, 37.5) | 58.5(54.6, 62.3) | 66.9(63.6, 70.1) | 61.1(56.9, 65.3) | 65.4(61.3, 69.5) | 31.0(25.8, 36.2) |
| Samoa | 2019 | EAP | L | 84.7(80.1, 89.3) | 98.6(97.5, 99.7) | 98.6(96.0, 101.3) | 98.8(97.6, 99.9) | 99.7(99.1, 100.2) | 15.0(10.3, 19.6) |
| STP | 2019 | SSA | LM | 7.8(4.0, 11.7) | 13.1(8.0, 18.2) | 30.4(24.4, 36.4) | 66.3(58.1, 74.5) | 88.4(83.2, 93.7) | 80.6(74.5, 86.7) |
| Senegal | 2019 | SSA | L | 16.2(12.3, 20.1) | 46.2(40.2, 52.3) | 65.4(59.2, 71.6) | 77.1(68.3, 85.9) | 85.9(80.0, 91.9) | 69.7(62.6, 76.8) |
| Sierra Leone | 2019 | SSA | L | 2.5(1.4, 3.7) | 6.0(4.3, 7.7) | 11.2(8.6, 13.8) | 23.7(20.8, 26.6) | 36.8(31.3, 42.3) | 34.3(28.7, 39.9) |
| South Africa | 2016 | SSA | UM | 32.2(25.6, 38.8) | 31.3(26.7, 35.9) | 50.0(43.2, 56.7) | 87.3(83.4, 91.1) | 98.5(97.1, 99.9) | 66.3(59.5, 73.1) |
| Suriname | 2018 | LAC | UM | 51.7(46.1, 57.2) | 90.2(87.3, 93.2) | 95.3(91.6, 99.1) | 99.0(98.5, 99.6) | 97.4(95.2, 99.7) | 45.8(39.8, 51.8) |
| Tajikistan | 2017 | ECA | LM | 98.3(97.2, 99.4) | 98.5(97.3, 99.6) | 98.1(96.9, 99.3) | 96.9(94.7, 99.0) | 94.1(90.9, 97.3) | -4.2(-7.6, -0.8) |
| Timor-Leste | 2016 | EAP | LM | 13.5(10.6, 16.5) | 38.7(34.3, 43.1) | 53.9(49.7, 58.1) | 70.5(66.3, 74.7) | 83.8(80.1, 87.5) | 70.3(65.5, 75.0) |
| Togo | 2017 | SSA | L | 1.3(0.0, 2.6) | 4.6(2.1, 7.2) | 8.9(5.6, 12.2) | 12.8(9.0, 16.6) | 54.1(48.4, 59.8) | 52.8(46.9, 58.7) |
| Tonga | 2019 | EAP | LM | 79.9(73.0, 86.8) | 87.1(80.7, 93.4) | 95.6(91.9, 99.2) | 99.6(98.9, 100.4) | 100.0(100.0, 100.0) | 20.1(13.2, 27.1) |
| Tunisia | 2018 | MENA | LM | 89.4(86.4, 92.5) | 93.6(91.1, 96.0) | 98.7(97.6, 99.9) | 99.1(98.0, 100.2) | 99.8(99.4, 100.2) | 10.4(7.3, 13.4) |
| Tuvalu | 2019 | EAP | UM | 65.8(52.2, 79.4) | 77.3(65.4, 89.2) | 87.1(77.6, 96.6) | 91.3(84.9, 97.7) | 97.5(94.4, 100.6) | 31.7(17.2, 46.1) |
| Uganda | 2016 | SSA | L | 3.2(1.9, 4.5) | 7.4(5.9, 9.0) | 13.1(11.0, 15.2) | 28.1(24.9, 31.3) | 38.2(34.4, 42.0) | 35.0(31.0, 39.0) |
| Zambia | 2018 | SSA | LM | 17.2(14.1, 20.4) | 24.6(21.2, 28.0) | 25.7(22.2, 29.2) | 24.1(20.1, 28.1) | 58.2(51.6, 64.9) | 41.0(33.6, 48.3) |
| Zimbabwe | 2019 | SSA | L | 8.0(5.9, 10.2) | 25.4(21.7, 29.1) | 47.1(42.9, 51.4) | 29.8(25.0, 34.7) | 48.8(43.2, 54.5) | 40.8(34.8, 46.8) |

**Note:**

1. World Bank region group: “EAP” represents “East Asia and Pacific”; “ECA” represents “Europe and Central Asia”; “LAC” represents “Latin America and the Caribbean”; “MENA” represents “Middle East and North Africa”; “SA” represents “South Asia”; “SSA” represents “Sub-Saharan Africa”.

2. Income group: “L” represents “Low-income country”; “LM” represents “Lower-middle income country”; “UM” represents “Upper-middle income country”.

*. The disparity in prevalence between young children living in the wealthiest and poorest quintiles is not statistically significant at the 0.05 level.

**Table S14.** National prevalence of young children living in households with ownership of landline or mobile phones and associated disparities by place of residence

| Country | Year | WB region^1^ | Income group^2^ | National (%) | Rural (%) | Urban (%) | Difference  (urban-rural) |
| --- | --- | --- | --- | --- | --- | --- | --- |
| Algeria | 2019 | MENA | UM | 95.3(94.6, 96.0) | 94.4(93.2, 95.6) | 95.9(95.1, 96.7) | 1.5(0.0, 2.9) |
| Angola | 2016 | SSA | UM | 64.3(62.2, 66.4) | 35.8(32.5, 39.0) | 83.1(81.1, 85.0) | 47.3(43.5, 51.1) |
| Armenia* | 2016 | ECA | LM | 99.5(99.0, 100.0) | 99.0(97.9, 100.0) | 99.9(99.8, 100.1) | 1.0(-0.1, 2.1) |
| Bangladesh | 2019 | SA | LM | 97.0(96.7, 97.3) | 96.9(96.5, 97.2) | 97.7(97.0, 98.3) | 0.8(0.1, 1.5) |
| Benin | 2018 | SSA | L | 86.5(85.2, 87.8) | 84.4(82.6, 86.1) | 89.9(88.1, 91.7) | 5.5(3.0, 8.0) |
| Burundi | 2017 | SSA | L | 49.9(48.3, 51.6) | 46.6(44.9, 48.3) | 83.2(79.6, 86.8) | 36.6(32.6, 40.6) |
| CAR | 2018 | SSA | L | 30.5(28.2, 32.9) | 15.8(13.5, 18.2) | 65.4(60.4, 70.5) | 49.6(44.0, 55.2) |
| Cameroon | 2018 | SSA | LM | 86.5(85.0, 88.0) | 79.9(77.5, 82.4) | 94.7(93.4, 95.9) | 14.8(12.0, 17.5) |
| Chad | 2019 | SSA | L | 70.8(69.3, 72.3) | 67.5(65.8, 69.3) | 87.7(85.5, 89.8) | 20.1(17.3, 22.9) |
| Congo DR | 2018 | SSA | L | 45.9(42.8, 49.0) | 27.4(24.2, 30.7) | 75.2(71.6, 78.7) | 47.7(42.9, 52.5) |
| Cote d’Ivoire | 2016 | SSA | LM | 92.8(91.5, 94.1) | 89.9(87.9, 91.9) | 97.4(96.5, 98.3) | 7.5(5.3, 9.6) |
| Dominican Republic | 2019 | LAC | UM | 93.5(92.5, 94.6) | 89.5(86.8, 92.2) | 94.9(93.9, 95.9) | 5.4(2.5, 8.3) |
| Ethiopia | 2016 | SSA | L | 51.6(48.7, 54.5) | 47.1(44.0, 50.2) | 88.2(82.8, 93.5) | 41.1(34.9, 47.2) |
| Gambia* | 2020 | SSA | L | 98.7(98.3, 99.1) | 98.1(97.3, 98.9) | 99.0(98.5, 99.5) | 0.9(-0.1, 1.8) |
| Ghana | 2017 | SSA | LM | 90.5(89.3, 91.8) | 86.8(84.9, 88.7) | 95.5(94.1, 96.8) | 8.7(6.3, 11.0) |
| Guinea | 2018 | SSA | L | 90.8(89.4, 92.2) | 88.4(86.5, 90.2) | 96.9(95.8, 98.0) | 8.5(6.4, 10.6) |
| Guinea-Bissau | 2019 | SSA | L | 95.6(94.5, 96.6) | 94.8(93.4, 96.1) | 98.0(96.9, 99.0) | 3.2(1.5, 4.9) |
| Guyana | 2019 | LAC | UM | 91.4(89.4, 93.4) | 89.8(87.3, 92.3) | 96.2(94.0, 98.3) | 6.4(3.1, 9.7) |
| Haiti | 2017 | LAC | L | 74.5(71.9, 77.2) | 67.8(64.4, 71.3) | 86.9(83.2, 90.5) | 19.0(14.0, 24.1) |
| India | 2016 | SA | LM | 92.9(92.7, 93.0) | 91.2(90.9, 91.4) | 97.1(96.8, 97.4) | 6.0(5.6, 6.3) |
| Indonesia | 2017 | EAP | LM | 94.1(93.5, 94.7) | 91.0(89.9, 92.0) | 97.5(97.0, 98.0) | 6.5(5.3, 7.7) |
| Iraq* | 2018 | MENA | UM | 98.9(98.6, 99.1) | 98.6(98.1, 99.1) | 99.0(98.6, 99.4) | 0.4(-0.2, 1.0) |
| Kiribati | 2018 | EAP | LM | 73.0(70.2, 75.9) | 57.7(53.3, 62.1) | 86.7(83.4, 90.0) | 29.0(23.5, 34.5) |
| Kyrgyzstan | 2018 | ECA | LM | 81.0(78.8, 83.1) | 83.0(80.4, 85.7) | 76.4(72.6, 80.1) | -6.7(-11.3, -2.1) |
| Lao | 2017 | EAP | LM | 89.3(88.1, 90.4) | 86.0(84.5, 87.6) | 97.9(97.1, 98.7) | 11.8(10.1, 13.6) |
| Lesotho | 2018 | SSA | LM | 93.6(92.5, 94.7) | 91.3(89.8, 92.8) | 98.0(96.6, 99.5) | 6.7(4.6, 8.8) |
| Liberia | 2019 | SSA | L | 68.6(65.7, 71.6) | 51.5(47.3, 55.7) | 83.7(80.2, 87.1) | 32.2(26.7, 37.6) |
| Madagascar | 2018 | SSA | L | 42.2(40.2, 44.3) | 36.7(34.4, 39.1) | 63.8(59.4, 68.1) | 27.0(22.1, 32.0) |
| Malawi | 2020 | SSA | L | 54.0(52.4, 55.7) | 49.4(47.7, 51.1) | 85.1(82.4, 87.8) | 35.7(32.5, 38.9) |
| Maldives* | 2017 | SA | UM | 99.6(99.3, 100.0) | 99.6(99.3, 100.0) | 99.6(98.9, 100.4) | 0.0(-0.8, 0.8) |
| Mali | 2018 | SSA | L | 92.0(90.8, 93.2) | 90.7(89.2, 92.2) | 97.0(96.0, 98.0) | 6.3(4.5, 8.0) |
| Mongolia | 2018 | EAP | LM | 99.5(99.2, 99.8) | 98.9(98.1, 99.6) | 99.8(99.6, 100.1) | 1.0(0.2, 1.7) |
| Myanmar | 2016 | EAP | LM | 67.2(63.7, 70.7) | 60.2(56.1, 64.4) | 91.4(88.4, 94.3) | 31.1(26.0, 36.2) |
| Nepal | 2019 | SA | L | 96.8(96.2, 97.5) | 95.6(94.5, 96.7) | 97.5(96.7, 98.3) | 1.9(0.5, 3.3) |
| Nigeria | 2018 | SSA | LM | 89.3(88.5, 90.2) | 85.2(83.9, 86.5) | 95.9(95.2, 96.6) | 10.7(9.3, 12.2) |
| PNG | 2016-18 | SA | LM | 56.8(53.6, 60.1) | 53.1(49.5, 56.7) | 89.2(85.1, 93.3) | 36.2(30.7, 41.6) |
| Pakistan | 2018 | EAP | LM | 94.5(93.6, 95.4) | 92.9(91.6, 94.1) | 98.0(97.2, 98.7) | 5.1(3.6, 6.6) |
| Palestine* | 2019 | MENA | LM | 98.4(97.9, 98.8) | 98.3(97.5, 99.0) | 98.4(97.8, 99.0) | 0.1(-0.8, 1.1) |
| Paraguay | 2016 | LAC | UM | 96.2(95.1, 97.2) | 93.5(91.5, 95.4) | 98.0(96.7, 99.2) | 4.5(2.2, 6.8) |
| Philippines | 2017 | EAP | LM | 89.5(88.3, 90.8) | 86.6(84.7, 88.5) | 93.2(91.6, 94.8) | 6.5(4.1, 9.0) |
| Rwanda | 2020 | SSA | L | 72.0(70.4, 73.5) | 68.0(66.1, 69.8) | 90.7(88.1, 93.4) | 22.7(19.5, 26.0) |
| Samoa* | 2019 | EAP | L | 93.5(91.9, 95.2) | 93.6(91.7, 95.5) | 93.2(89.9, 96.5) | -0.4(-4.2, 3.5) |
| STP* | 2019 | SSA | LM | 86.2(83.9, 88.5) | 87.7(85.1, 90.3) | 85.5(82.2, 88.7) | -2.3(-6.4, 1.9) |
| Senegal | 2019 | SSA | L | 97.4(96.6, 98.2) | 96.4(95.2, 97.7) | 99.0(98.5, 99.5) | 2.5(1.2, 3.9) |
| Sierra Leone | 2019 | SSA | L | 72.5(70.5, 74.4) | 60.8(58.2, 63.3) | 94.2(92.9, 95.5) | 33.4(30.5, 36.3) |
| South Africa* | 2016 | SSA | UM | 97.6(96.8, 98.4) | 97.4(96.4, 98.5) | 97.7(96.6, 98.8) | 0.2(-1.3, 1.8) |
| Suriname | 2018 | LAC | UM | 97.2(96.3, 98.1) | 94.9(92.9, 96.9) | 98.4(97.6, 99.3) | 3.5(1.3, 5.7) |
| Tajikistan | 2017 | ECA | LM | 97.4(96.5, 98.2) | 97.1(96.0, 98.1) | 98.5(97.8, 99.1) | 1.4(0.2, 2.6) |
| Timor-Leste | 2016 | EAP | LM | 92.5(91.4, 93.6) | 90.1(88.6, 91.5) | 98.6(98.0, 99.3) | 8.5(7.0, 10.1) |
| Togo | 2017 | SSA | L | 86.5(84.7, 88.2) | 80.9(78.6, 83.2) | 95.4(93.5, 97.3) | 14.5(11.5, 17.5) |
| Tonga* | 2019 | EAP | LM | 98.9(98.2, 99.6) | 98.8(98.0, 99.7) | 99.0(97.9, 100.1) | 0.1(-1.3, 1.5) |
| Tunisia | 2018 | MENA | LM | 97.5(96.8, 98.2) | 96.4(94.8, 98.0) | 98.1(97.4, 98.8) | 1.7(0.0, 3.5) |
| Tuvalu | 2019 | EAP | UM | 94.4(91.7, 97.1) | 86.7(80.2, 93.2) | 98.4(96.0, 100.7) | 11.6(4.6, 18.7) |
| Uganda | 2016 | SSA | L | 77.4(76.0, 78.7) | 73.7(72.1, 75.3) | 90.9(88.5, 93.4) | 17.2(14.3, 20.1) |
| Zambia | 2018 | SSA | LM | 72.6(70.8, 74.5) | 62.4(60.1, 64.6) | 91.3(89.7, 93.0) | 29.0(26.2, 31.8) |
| Zimbabwe | 2019 | SSA | L | 88.4(87.1, 89.6) | 84.9(83.3, 86.6) | 96.7(95.5, 97.8) | 11.7(9.7, 13.7) |

**Note:**

1. World Bank region group: “EAP” represents “East Asia and Pacific”; “ECA” represents “Europe and Central Asia”; “LAC” represents “Latin America and the Caribbean”; “MENA” represents “Middle East and North Africa”; “SA” represents “South Asia”; “SSA” represents “Sub-Saharan Africa”.

2. Income group: “L” represents “Low-income country”; “LM” represents “Lower-middle income country”; “UM” represents “Upper-middle income country”.

*. The disparity in prevalence between young children in urban and rural areas is not statistically significant at the 0.05 level.

**Table S15.** National prevalence of young children living in households with ownership of landline or mobile phones and associated disparities by household wealth quintile

| **Country** | **Year** | **WB region^1^** | **Income group^2^** | **Wealth** | | | | | **Difference (richest-poorest)** |
| --- | --- | --- | --- | --- | --- | --- | --- | --- | --- |
|  |  |  |  | **Poorest (%)** | **2 (%)** | **3 (%)** | **4 (%)** | **Richest (%)** |  |
| Algeria | 2019 | MENA | UM | 90.2(88.3, 92.0) | 94.8(93.7, 95.9) | 96.1(94.9, 97.2) | 98.0(97.2, 98.8) | 99.7(99.4, 100.0) | 9.5(7.7, 11.4) |
| Angola | 2016 | SSA | UM | 23.2(19.8, 26.5) | 42.8(39.5, 46.1) | 79.7(77.1, 82.4) | 96.6(95.2, 98.0) | 99.8(99.5, 100.2) | 76.7(73.3, 80.1) |
| Armenia* | 2016 | ECA | LM | 98.0(95.7, 100.3) | 99.8(99.4, 100.2) | 99.7(99.1, 100.3) | 100.0(100.0, 100.0) | 100.0(100.0, 100.0) | 2.0(-0.3, 4.3) |
| Bangladesh | 2019 | SA | LM | 92.7(91.8, 93.7) | 97.7(97.2, 98.3) | 98.4(97.9, 98.9) | 98.1(97.5, 98.7) | 98.6(98.2, 99.1) | 5.9(4.9, 6.9) |
| Benin | 2018 | SSA | L | 66.3(63.3, 69.3) | 82.2(79.5, 84.8) | 91.8(90.2, 93.4) | 96.4(95.5, 97.4) | 98.6(97.9, 99.3) | 32.3(29.2, 35.3) |
| Burundi | 2017 | SSA | L | 8.1(6.6, 9.6) | 29.0(26.3, 31.6) | 55.7(52.8, 58.6) | 78.3(75.7, 80.9) | 93.9(92.5, 95.4) | 85.8(83.8, 87.9) |
| CAR | 2018 | SSA | L | 2.7(1.0, 4.4) | 8.7(6.3, 11.1) | 23.9(20.2, 27.7) | 53.3(48.5, 58.1) | 86.7(83.4, 89.9) | 84.0(80.4, 87.5) |
| Cameroon | 2018 | SSA | LM | 65.0(60.2, 69.7) | 85.4(82.6, 88.2) | 92.3(90.4, 94.1) | 97.4(96.1, 98.7) | 98.5(97.5, 99.4) | 33.5(28.6, 38.4) |
| Chad | 2019 | SSA | L | 43.7(41.0, 46.5) | 66.5(63.7, 69.3) | 73.0(70.5, 75.5) | 84.4(82.0, 86.7) | 92.6(90.9, 94.2) | 48.9(45.6, 52.1) |
| Congo DR | 2018 | SSA | L | 8.1(6.3, 10.0) | 29.0(25.6, 32.3) | 47.6(42.5, 52.8) | 73.2(69.1, 77.2) | 90.8(87.3, 94.3) | 82.7(78.7, 86.7) |
| Cote d’Ivoire | 2016 | SSA | LM | 83.1(79.4, 86.8) | 92.4(90.4, 94.3) | 97.4(96.3, 98.5) | 97.7(96.4, 99.0) | 98.3(96.8, 99.7) | 15.1(11.1, 19.1) |
| Dominican Republic | 2019 | LAC | UM | 81.7(78.6, 84.9) | 92.9(90.4, 95.4) | 97.5(95.9, 99.0) | 99.4(98.7, 100.1) | 99.9(99.8, 100.0) | 18.2(15.0, 21.4) |
| Ethiopia | 2016 | SSA | L | 28.9(25.0, 32.8) | 35.7(30.6, 40.9) | 47.1(41.9, 52.2) | 73.8(69.5, 78.2) | 93.1(89.7, 96.6) | 64.2(59.1, 69.3) |
| Gambia | 2020 | SSA | L | 97.5(96.4, 98.6) | 98.2(97.0, 99.5) | 99.4(98.8, 100.0) | 99.3(98.8, 99.9) | 99.3(98.1, 100.4) | 1.8(0.2, 3.4) |
| Ghana | 2017 | SSA | LM | 76.1(72.4, 79.8) | 87.6(84.8, 90.3) | 95.0(93.4, 96.7) | 97.5(96.0, 99.0) | 99.2(98.3, 100.1) | 23.1(19.3, 26.9) |
| Guinea | 2018 | SSA | L | 79.2(75.9, 82.5) | 89.4(86.1, 92.8) | 95.0(93.1, 96.9) | 96.1(94.5, 97.7) | 98.9(98.0, 99.7) | 19.7(16.2, 23.1) |
| Guinea-Bissau | 2019 | SSA | L | 88.9(85.9, 91.9) | 95.2(93.4, 97.1) | 98.1(96.9, 99.2) | 98.4(97.1, 99.6) | 99.3(98.5, 100.1) | 10.4(7.3, 13.5) |
| Guyana | 2019 | LAC | UM | 78.3(73.1, 83.6) | 91.1(87.1, 95.2) | 97.2(94.4, 100.0) | 98.8(97.4, 100.3) | 100.0(100.0, 100.0) | 21.7(16.4, 26.9) |
| Haiti | 2017 | LAC | L | 43.1(38.4, 47.8) | 70.3(66.1, 74.5) | 81.4(76.9, 85.9) | 95.0(93.2, 96.9) | 98.5(97.3, 99.7) | 55.4(50.6, 60.3) |
| India | 2016 | SA | LM | 78.4(77.9, 79.0) | 94.6(94.3, 95.0) | 98.2(98.0, 98.4) | 99.5(99.3, 99.6) | 99.7(99.6, 99.9) | 21.3(20.7, 21.9) |
| Indonesia | 2017 | EAP | LM | 77.5(75.3, 79.7) | 95.2(94.0, 96.3) | 98.9(98.4, 99.4) | 99.8(99.7, 99.9) | 100.0(99.9, 100.0) | 22.4(20.2, 24.6) |
| Iraq | 2018 | MENA | UM | 97.0(96.1, 98.0) | 98.9(98.5, 99.4) | 99.1(98.5, 99.8) | 99.6(99.3, 99.9) | 99.9(99.8, 100.1) | 2.9(1.9, 3.8) |
| Kiribati | 2018 | EAP | LM | 39.9(34.3, 45.6) | 63.8(58.2, 69.5) | 78.0(72.1, 83.9) | 91.0(86.8, 95.2) | 98.7(97.5, 99.8) | 58.7(52.9, 64.6) |
| Kyrgyzstan | 2018 | ECA | LM | 83.8(79.4, 88.2) | 82.9(78.8, 87.0) | 81.0(75.5, 86.6) | 82.5(78.6, 86.4) | 71.6(66.5, 76.7) | -12.2(-19.0, -5.4) |
| Lao | 2017 | EAP | LM | 66.4(63.3, 69.5) | 92.8(91.2, 94.3) | 97.6(96.7, 98.5) | 99.4(99.0, 99.8) | 99.9(99.8, 100.1) | 33.5(30.4, 36.7) |
| Lesotho | 2018 | SSA | LM | 81.6(78.2, 85.0) | 94.8(92.7, 96.9) | 97.0(95.5, 98.5) | 97.8(95.5, 100.1) | 99.4(98.1, 100.6) | 17.8(14.2, 21.4) |
| Liberia | 2019 | SSA | L | 28.8(25.1, 32.4) | 61.1(56.2, 66.0) | 77.3(73.8, 80.9) | 93.3(90.5, 96.1) | 98.6(97.1, 100.1) | 69.8(65.8, 73.8) |
| Madagascar | 2018 | SSA | L | 9.4(7.8, 11.0) | 24.2(21.6, 26.8) | 46.4(43.1, 49.6) | 69.1(65.9, 72.4) | 91.7(89.2, 94.2) | 82.3(79.3, 85.2) |
| Malawi | 2020 | SSA | L | 16.2(14.3, 18.0) | 42.0(39.3, 44.8) | 60.7(57.9, 63.5) | 81.0(78.7, 83.3) | 94.8(93.4, 96.1) | 78.6(76.4, 80.9) |
| Maldives* | 2017 | SA | UM | 99.5(98.6, 100.3) | 99.4(98.7, 100.1) | 100.0(100.0, 100.0) | 99.3(97.9, 100.7) | 100.0(100.0, 100.0) | 0.5(-0.3, 1.4) |
| Mali | 2018 | SSA | L | 85.7(82.7, 88.8) | 88.2(85.5, 90.8) | 92.8(90.9, 94.7) | 95.8(94.4, 97.2) | 99.2(98.7, 99.7) | 13.5(10.4, 16.6) |
| Mongolia | 2018 | EAP | LM | 98.2(97.0, 99.4) | 99.5(98.8, 100.2) | 100.0(100.0, 100.0) | 99.8(99.3, 100.2) | 100.0(100.0, 100.0) | 1.8(0.6, 3.0) |
| Myanmar | 2016 | EAP | LM | 32.2(27.8, 36.7) | 59.5(54.3, 64.7) | 83.2(78.1, 88.3) | 96.0(93.8, 98.2) | 99.7(99.2, 100.3) | 67.5(63.0, 72.0) |
| Nepal | 2019 | SA | L | 92.3(90.4, 94.1) | 96.6(95.0, 98.2) | 97.7(96.4, 99.0) | 99.4(98.8, 99.9) | 99.4(98.7, 100.1) | 7.2(5.2, 9.2) |
| Nigeria | 2018 | SSA | LM | 69.8(67.7, 72.0) | 87.7(86.1, 89.2) | 95.9(95.0, 96.8) | 98.5(98.0, 99.0) | 99.5(99.1, 99.9) | 29.6(27.5, 31.8) |
| PNG | 2016-18 | SA | LM | 12.6(9.5, 15.7) | 46.0(40.9, 51.1) | 61.9(57.0, 66.8) | 78.2(75.0, 81.4) | 93.4(90.5, 96.3) | 80.8(76.4, 85.2) |
| Pakistan | 2018 | EAP | LM | 83.8(80.8, 86.8) | 93.7(91.4, 96.1) | 97.9(96.8, 99.1) | 99.2(98.7, 99.8) | 100.0(100.0, 100.0) | 16.2(13.1, 19.2) |
| Palestine | 2019 | MENA | LM | 95.6(94.0, 97.2) | 99.2(98.5, 99.9) | 98.3(97.3, 99.3) | 99.7(99.4, 100.1) | 99.6(98.8, 100.4) | 4.0(2.2, 5.8) |
| Paraguay | 2016 | LAC | UM | 86.1(82.4, 89.7) | 98.3(97.3, 99.3) | 99.9(99.6, 100.1) | 100.0(100.0, 100.0) | 100.0(100.0, 100.0) | 13.9(10.3, 17.6) |
| Philippines | 2017 | EAP | LM | 73.0(69.8, 76.2) | 90.1(87.7, 92.5) | 97.2(96.0, 98.5) | 99.5(99.0, 100.0) | 99.6(98.9, 100.3) | 26.6(23.4, 29.8) |
| Rwanda | 2020 | SSA | L | 27.6(24.9, 30.2) | 64.3(61.3, 67.3) | 85.4(82.8, 88.0) | 94.7(93.1, 96.2) | 99.2(98.5, 99.8) | 71.6(68.8, 74.4) |
| Samoa | 2019 | EAP | L | 86.0(80.6, 91.4) | 94.7(91.9, 97.4) | 93.1(89.6, 96.6) | 97.4(95.5, 99.3) | 99.5(98.9, 100.2) | 13.5(8.0, 19.0) |
| STP | 2019 | SSA | LM | 68.8(62.7, 74.9) | 83.2(77.8, 88.6) | 91.6(88.1, 95.2) | 95.5(92.3, 98.8) | 96.6(94.3, 98.8) | 27.7(21.1, 34.3) |
| Senegal | 2019 | SSA | L | 94.2(92.3, 96.2) | 98.2(97.3, 99.1) | 95.6(92.8, 98.5) | 99.8(99.5, 100.1) | 99.9(99.7, 100.1) | 5.7(3.7, 7.6) |
| Sierra Leone | 2019 | SSA | L | 34.1(30.8, 37.3) | 68.7(65.5, 71.9) | 79.5(76.6, 82.4) | 95.7(94.4, 97.1) | 99.0(98.3, 99.8) | 65.0(61.6, 68.4) |
| South Africa | 2016 | SSA | UM | 93.2(90.2, 96.3) | 98.2(97.0, 99.4) | 98.5(97.3, 99.6) | 99.2(98.3, 100.1) | 100.0(100.0, 100.0) | 6.8(3.7, 9.8) |
| Suriname | 2018 | LAC | UM | 93.4(90.9, 95.9) | 98.5(97.5, 99.5) | 98.3(96.3, 100.2) | 99.5(98.6, 100.3) | 100.0(100.0, 100.0) | 6.6(4.1, 9.1) |
| Tajikistan | 2017 | ECA | LM | 91.8(88.5, 95.2) | 97.8(96.5, 99.2) | 98.6(97.3, 99.9) | 99.5(99.1, 99.9) | 98.5(97.8, 99.2) | 6.6(3.2, 10.1) |
| Timor-Leste | 2016 | EAP | LM | 77.7(73.9, 81.6) | 91.0(88.7, 93.2) | 95.7(94.3, 97.2) | 99.0(98.3, 99.6) | 99.7(99.4, 100.0) | 21.9(18.1, 25.8) |
| Togo | 2017 | SSA | L | 64.7(60.0, 69.5) | 87.4(84.1, 90.7) | 88.3(84.6, 92.0) | 95.9(93.3, 98.5) | 99.5(98.8, 100.2) | 34.8(30.0, 39.6) |
| Tonga | 2019 | EAP | LM | 98.4(97.1, 99.7) | 98.0(95.3, 100.6) | 99.0(97.9, 100.0) | 99.4(98.2, 100.6) | 100.0(100.0, 100.0) | 1.6(0.3, 2.9) |
| Tunisia | 2018 | MENA | LM | 94.2(91.8, 96.7) | 97.8(96.6, 99.0) | 96.7(95.0, 98.4) | 99.0(97.8, 100.1) | 99.7(99.2, 100.2) | 5.5(2.9, 8.0) |
| Tuvalu | 2019 | EAP | UM | 83.1(74.7, 91.6) | 93.3(86.1, 100.6) | 97.5(94.0, 101.0) | 99.0(97.0, 101.0) | 100.0(100.0, 100.0) | 16.9(8.2, 25.5) |
| Uganda | 2016 | SSA | L | 40.1(37.7, 42.4) | 71.3(68.8, 73.9) | 89.3(87.4, 91.1) | 95.5(94.3, 96.8) | 98.7(98.0, 99.4) | 58.6(56.2, 61.1) |
| Zambia | 2018 | SSA | LM | 32.9(30.1, 35.8) | 68.2(65.0, 71.4) | 88.1(86.2, 90.1) | 93.7(91.8, 95.5) | 98.9(98.0, 99.7) | 65.9(63.0, 68.9) |
| Zimbabwe | 2019 | SSA | L | 72.2(68.6, 75.8) | 85.8(83.2, 88.5) | 94.4(92.5, 96.2) | 95.3(93.8, 96.8) | 99.3(98.7, 99.9) | 27.1(23.5, 30.8) |

**Note:**

1. World Bank region group: “EAP” represents “East Asia and Pacific”; “ECA” represents “Europe and Central Asia”; “LAC” represents “Latin America and the Caribbean”; “MENA” represents “Middle East and North Africa”; “SA” represents “South Asia”; “SSA” represents “Sub-Saharan Africa”.

2. Income group: “L” represents “Low-income country”; “LM” represents “Lower-middle income country”; “UM” represents “Upper-middle income country”.

*. The disparity in prevalence between young children living in the wealthiest and poorest quintiles is not statistically significant at the 0.05 level.

**Table S16.** National prevalence of young children with mother exposed to mass media at least once a week and associated disparities by place of residence

| Country | Year | WB region^1^ | Income group^2^ | National (%) | Rural (%) | Urban (%) | Difference  (urban-rural) |
| --- | --- | --- | --- | --- | --- | --- | --- |
| Algeria* | 2019 | MENA | UM | 89.6(88.6, 90.6) | 88.6(86.9, 90.3) | 90.3(89.1, 91.6) | 1.8(-0.4, 3.9) |
| Angola | 2016 | SSA | UM | 66.2(64.0, 68.5) | 39.9(36.4, 43.3) | 83.6(81.4, 85.7) | 43.7(39.6, 47.8) |
| Armenia* | 2016 | ECA | LM | 93.6(92.1, 95.1) | 92.6(90.4, 94.9) | 94.3(92.2, 96.3) | 1.6(-1.4, 4.7) |
| Bangladesh | 2019 | SA | LM | 61.0(59.9, 62.1) | 55.4(54.1, 56.7) | 81.7(79.9, 83.6) | 26.3(24.1, 28.5) |
| Benin | 2018 | SSA | L | 40.2(38.1, 42.2) | 34.3(31.8, 36.7) | 49.5(46.0, 53.0) | 15.2(10.9, 19.5) |
| Burundi | 2017 | SSA | L | 27.9(26.5, 29.3) | 26.1(24.7, 27.5) | 45.6(40.0, 51.2) | 19.5(13.7, 25.3) |
| CAR | 2018 | SSA | L | 19.1(17.2, 21.1) | 11.4(9.2, 13.6) | 37.4(33.6, 41.3) | 26.0(21.6, 30.5) |
| Cameroon | 2018 | SSA | LM | 41.7(38.7, 44.7) | 18.6(15.5, 21.7) | 70.2(66.4, 74.0) | 51.6(46.6, 56.5) |
| Chad | 2019 | SSA | L | 15.7(14.3, 17.1) | 11.1(9.7, 12.5) | 40.4(35.9, 45.0) | 29.3(24.6, 34.1) |
| Congo DR | 2018 | SSA | L | 27.0(24.3, 29.7) | 9.7(7.2, 12.2) | 54.2(48.7, 59.7) | 44.4(38.4, 50.5) |
| Cote d’Ivoire | 2016 | SSA | LM | 59.8(57.0, 62.7) | 44.6(41.0, 48.2) | 83.7(80.7, 86.7) | 39.1(34.3, 43.8) |
| Dominican Republic | 2019 | LAC | UM | 81.5(80.1, 83.0) | 77.8(74.8, 80.7) | 82.8(81.1, 84.5) | 5.0(1.6, 8.4) |
| Ethiopia | 2016 | SSA | L | 18.2(16.3, 20.2) | 12.5(10.7, 14.3) | 64.2(58.0, 70.4) | 51.7(45.3, 58.2) |
| Gambia | 2020 | SSA | L | 68.2(65.6, 70.7) | 54.4(50.2, 58.6) | 75.5(72.4, 78.5) | 21.1(15.8, 26.3) |
| Ghana | 2017 | SSA | LM | 75.1(73.0, 77.2) | 66.3(63.3, 69.2) | 86.6(84.1, 89.2) | 20.4(16.5, 24.3) |
| Guinea | 2018 | SSA | L | 41.2(38.5, 43.9) | 31.9(28.6, 35.2) | 64.7(60.7, 68.6) | 32.8(27.5, 38.1) |
| Guinea-Bissau* | 2019 | SSA | L | 77.6(75.6, 79.6) | 76.8(74.5, 79.2) | 79.9(75.9, 83.9) | 3.1(-1.6, 7.7) |
| Guyana | 2019 | LAC | UM | 83.0(80.5, 85.6) | 80.8(77.8, 83.8) | 89.7(85.9, 93.5) | 8.9(4.0, 13.8) |
| Haiti | 2017 | LAC | L | 62.4(59.5, 65.3) | 53.3(49.2, 57.4) | 79.3(76.3, 82.2) | 26.0(20.9, 31.0) |
| India | 2016 | SA | LM | 65.2(64.7, 65.6) | 56.5(56.0, 56.9) | 87.4(86.7, 88.1) | 31.0(30.1, 31.8) |
| Indonesia | 2017 | EAP | LM | 85.8(84.9, 86.7) | 82.2(80.7, 83.8) | 89.6(88.7, 90.5) | 7.4(5.6, 9.2) |
| Iraq | 2018 | MENA | UM | 92.2(91.4, 93.1) | 90.2(88.1, 92.4) | 93.2(92.3, 94.0) | 2.9(0.6, 5.3) |
| Kiribati | 2018 | EAP | LM | 39.5(36.0, 42.9) | 29.7(25.8, 33.6) | 47.9(42.5, 53.3) | 18.3(11.5, 25.0) |
| Kyrgyzstan* | 2018 | ECA | LM | 95.6(94.7, 96.6) | 95.2(93.8, 96.5) | 96.6(95.4, 97.8) | 1.5(-0.3, 3.2) |
| Lao | 2017 | EAP | LM | 71.1(69.1, 73.2) | 64.5(61.9, 67.0) | 89.0(87.1, 90.9) | 24.5(21.4, 27.7) |
| Lesotho | 2018 | SSA | LM | 58.7(55.8, 61.6) | 46.7(42.8, 50.6) | 79.3(75.1, 83.5) | 32.6(26.9, 38.3) |
| Liberia | 2019 | SSA | L | 28.7(25.5, 31.9) | 19.8(17.1, 22.5) | 36.6(31.1, 42.0) | 16.8(10.7, 22.9) |
| Madagascar | 2018 | SSA | L | 39.4(37.3, 41.5) | 33.0(30.7, 35.3) | 64.9(60.1, 69.6) | 31.9(26.6, 37.2) |
| Malawi | 2020 | SSA | L | 39.8(38.1, 41.5) | 35.6(33.8, 37.3) | 67.9(63.3, 72.5) | 32.3(27.4, 37.3) |
| Maldives | 2017 | SA | UM | 93.2(91.9, 94.4) | 91.2(89.8, 92.7) | 96.7(94.3, 99.0) | 5.4(2.6, 8.2) |
| Mali | 2018 | SSA | L | 56.9(54.4, 59.4) | 51.8(48.9, 54.8) | 77.2(73.9, 80.5) | 25.3(20.9, 29.8) |
| Mongolia | 2018 | EAP | LM | 93.1(91.9, 94.3) | 90.2(88.3, 92.2) | 94.6(93.1, 96.0) | 4.3(1.9, 6.8) |
| Myanmar | 2016 | EAP | LM | 57.8(54.6, 60.9) | 50.4(46.7, 54.0) | 83.6(79.7, 87.6) | 33.3(27.9, 38.7) |
| Nepal | 2019 | SA | L | 59.9(56.7, 63.1) | 49.1(45.0, 53.2) | 65.7(61.4, 69.9) | 16.6(10.6, 22.5) |
| Nigeria | 2018 | SSA | LM | 38.7(37.3, 40.1) | 26.4(24.8, 28.0) | 58.3(56.0, 60.7) | 32.0(29.1, 34.8) |
| PNG | 2016-18 | SA | LM | 24.7(21.4, 28.0) | 20.8(17.1, 24.5) | 58.1(51.4, 64.9) | 37.4(29.7, 45.1) |
| Pakistan | 2018 | EAP | LM | 48.8(45.5, 52.1) | 38.9(34.8, 42.9) | 69.9(66.2, 73.6) | 31.0(25.5, 36.5) |
| Palestine* | 2019 | MENA | LM | 75.1(73.4, 76.8) | 77.3(74.9, 79.7) | 74.4(72.4, 76.5) | -2.9(-6.1, 0.3) |
| Paraguay | 2016 | LAC | UM | 94.7(93.4, 96.0) | 89.8(86.9, 92.6) | 98.0(97.2, 98.8) | 8.3(5.3, 11.2) |
| Philippines | 2017 | EAP | LM | 81.7(80.2, 83.2) | 78.1(75.7, 80.4) | 86.3(84.5, 88.2) | 8.3(5.3, 11.3) |
| Rwanda | 2020 | SSA | L | 59.6(57.9, 61.3) | 55.3(53.4, 57.2) | 80.1(76.8, 83.4) | 24.8(21.0, 28.6) |
| Samoa | 2019 | EAP | L | 68.7(64.9, 72.4) | 66.4(62.0, 70.8) | 80.5(75.7, 85.2) | 14.1(7.5, 20.6) |
| STP* | 2019 | SSA | LM | 93.3(91.7, 94.9) | 93.1(90.8, 95.4) | 93.4(91.3, 95.5) | 0.3(-2.8, 3.5) |
| Senegal | 2019 | SSA | L | 69.2(66.0, 72.3) | 60.6(56.7, 64.6) | 84.2(80.0, 88.3) | 23.5(17.8, 29.3) |
| Sierra Leone | 2019 | SSA | L | 26.7(24.6, 28.8) | 19.3(17.0, 21.6) | 40.4(36.5, 44.4) | 21.1(16.6, 25.7) |
| South Africa | 2016 | SSA | UM | 80.1(77.5, 82.8) | 69.9(65.1, 74.7) | 85.9(82.7, 89.2) | 16.0(10.3, 21.8) |
| Suriname | 2018 | LAC | UM | 85.0(82.7, 87.3) | 74.2(69.4, 79.0) | 90.6(88.4, 92.8) | 16.4(11.1, 21.7) |
| Tajikistan | 2017 | ECA | LM | 85.0(82.8, 87.3) | 84.2(81.4, 87.0) | 88.1(85.7, 90.5) | 3.9(0.2, 7.6) |
| Timor-Leste | 2016 | EAP | LM | 38.7(36.1, 41.2) | 27.3(24.2, 30.4) | 66.9(63.3, 70.4) | 39.6(34.8, 44.3) |
| Togo | 2017 | SSA | L | 54.8(51.2, 58.4) | 40.2(36.1, 44.4) | 77.7(72.9, 82.5) | 37.5(31.1, 43.8) |
| Tonga* | 2019 | EAP | LM | 80.3(76.5, 84.0) | 80.6(76.4, 84.7) | 79.1(70.5, 87.7) | -1.5(-11.1, 8.2) |
| Tunisia* | 2018 | MENA | LM | 96.1(95.2, 97.0) | 95.6(93.9, 97.3) | 96.4(95.3, 97.5) | 0.8(-1.2, 2.9) |
| Tuvalu* | 2019 | EAP | UM | 75.7(70.0, 81.4) | 76.2(68.5, 84.0) | 75.5(68.0, 82.9) | -0.8(-11.8, 10.2) |
| Uganda | 2016 | SSA | L | 62.2(60.7, 63.7) | 58.5(56.9, 60.1) | 75.9(72.4, 79.4) | 17.4(13.5, 21.3) |
| Zambia | 2018 | SSA | LM | 21.8(20.4, 23.2) | 17.3(15.7, 18.9) | 29.9(27.2, 32.7) | 12.6(9.5, 15.8) |
| Zimbabwe | 2019 | SSA | L | 58.1(55.7, 60.4) | 46.0(43.1, 48.9) | 86.0(83.1, 88.9) | 40.0(36.0, 44.1) |

**Note:**

1. World Bank region group: “EAP” represents “East Asia and Pacific”; “ECA” represents “Europe and Central Asia”; “LAC” represents “Latin America and the Caribbean”; “MENA” represents “Middle East and North Africa”; “SA” represents “South Asia”; “SSA” represents “Sub-Saharan Africa”.

2. Income group: “L” represents “Low-income country”; “LM” represents “Lower-middle income country”; “UM” represents “Upper-middle income country”.

*. The disparity in prevalence between young children in urban and rural areas is not statistically significant at the 0.05 level.

**Table S17.** National prevalence of young children with mother exposed to mass media at least once a week and associated disparities by household wealth quintile

| **Country** | **Year** | **WB region^1^** | **Income group^2^** | **Wealth** | | | | | **Difference (richest-poorest)** |
| --- | --- | --- | --- | --- | --- | --- | --- | --- | --- |
|  |  |  |  | **Poorest (%)** | **2 (%)** | **3 (%)** | **4 (%)** | **Richest (%)** |  |
| Algeria | 2019 | MENA | UM | 84.1(81.9, 86.2) | 90.2(88.2, 92.2) | 92.7(91.2, 94.2) | 90.9(88.7, 93.0) | 91.9(89.7, 94.1) | 7.8(4.7, 10.9) |
| Angola | 2016 | SSA | UM | 27.8(24.1, 31.5) | 47.4(43.8, 50.9) | 79.6(76.8, 82.5) | 96.8(95.4, 98.1) | 98.6(97.4, 99.8) | 70.8(66.9, 74.7) |
| Armenia | 2016 | ECA | LM | 92.3(88.8, 95.7) | 93.0(89.9, 96.2) | 93.8(90.1, 97.4) | 90.9(86.4, 95.4) | 96.9(94.3, 99.4) | 4.6(0.3, 8.9) |
| Bangladesh | 2019 | SA | LM | 21.6(19.9, 23.2) | 53.5(51.5, 55.5) | 66.9(64.9, 68.8) | 78.0(76.2, 79.8) | 88.2(86.7, 89.7) | 66.6(64.4, 68.9) |
| Benin | 2018 | SSA | L | 22.4(18.9, 25.8) | 27.7(24.6, 30.8) | 35.2(31.6, 38.9) | 46.5(43.2, 49.7) | 74.5(71.6, 77.4) | 52.1(47.6, 56.6) |
| Burundi | 2017 | SSA | L | 6.9(5.5, 8.4) | 18.8(16.5, 21.0) | 27.0(24.7, 29.3) | 40.6(37.4, 43.9) | 54.4(50.7, 58.1) | 47.4(43.5, 51.3) |
| CAR | 2018 | SSA | L | 5.5(3.1, 7.9) | 8.7(5.9, 11.5) | 17.2(13.4, 21.1) | 23.5(19.8, 27.2) | 53.3(49.0, 57.5) | 47.8(42.9, 52.7) |
| Cameroon | 2018 | SSA | LM | 2.4(1.3, 3.5) | 16.0(13.1, 18.9) | 40.0(35.9, 44.2) | 78.5(75.0, 82.0) | 93.0(91.0, 94.9) | 90.6(88.3, 92.8) |
| Chad | 2019 | SSA | L | 6.0(4.4, 7.6) | 8.8(7.1, 10.5) | 11.0(9.3, 12.7) | 17.3(14.3, 20.3) | 41.6(37.8, 45.4) | 35.7(31.5, 39.8) |
| Congo DR | 2018 | SSA | L | 5.0(2.4, 7.7) | 8.2(5.0, 11.3) | 15.5(12.0, 19.0) | 40.2(34.2, 46.2) | 83.8(78.0, 89.6) | 78.8(72.4, 85.1) |
| Cote d’Ivoire | 2016 | SSA | LM | 28.2(24.2, 32.2) | 42.2(37.3, 47.1) | 70.7(67.0, 74.4) | 88.8(86.0, 91.7) | 92.9(89.6, 96.1) | 64.7(59.5, 69.8) |
| Dominican Republic | 2019 | LAC | UM | 69.4(65.8, 73.0) | 83.2(80.4, 86.0) | 83.0(79.8, 86.2) | 85.8(82.2, 89.3) | 89.6(86.5, 92.8) | 20.2(15.2, 25.2) |
| Ethiopia | 2016 | SSA | L | 3.7(1.9, 5.5) | 7.4(4.9, 9.8) | 11.3(8.5, 14.1) | 23.7(19.2, 28.2) | 62.6(57.3, 67.8) | 58.8(53.4, 64.3) |
| Gambia | 2020 | SSA | L | 47.9(43.3, 52.5) | 56.4(52.0, 60.8) | 72.6(67.6, 77.6) | 83.0(79.4, 86.6) | 88.6(85.1, 92.1) | 40.7(35.0, 46.5) |
| Ghana | 2017 | SSA | LM | 42.4(38.1, 46.6) | 67.3(63.2, 71.3) | 83.5(80.2, 86.8) | 91.8(89.4, 94.2) | 95.9(94.0, 97.7) | 53.5(48.9, 58.2) |
| Guinea | 2018 | SSA | L | 30.4(25.7, 35.1) | 27.0(23.1, 30.8) | 34.3(29.3, 39.3) | 52.9(48.5, 57.2) | 73.8(69.5, 78.1) | 43.5(37.0, 49.9) |
| Guinea-Bissau | 2019 | SSA | L | 66.2(62.7, 69.8) | 75.5(71.6, 79.4) | 82.5(78.8, 86.2) | 80.5(76.3, 84.7) | 87.8(83.2, 92.4) | 21.6(15.7, 27.4) |
| Guyana | 2019 | LAC | UM | 62.9(56.8, 69.0) | 90.8(85.3, 96.2) | 90.0(85.4, 94.6) | 89.7(84.4, 95.0) | 92.5(88.5, 96.5) | 29.6(22.1, 37.1) |
| Haiti | 2017 | LAC | L | 38.3(33.5, 43.2) | 50.7(45.4, 55.9) | 67.0(62.3, 71.7) | 80.8(77.1, 84.5) | 90.4(87.8, 92.9) | 52.0(46.5, 57.5) |
| India | 2016 | SA | LM | 22.3(21.7, 22.9) | 56.2(55.4, 57.0) | 81.3(80.7, 81.9) | 92.4(91.9, 92.9) | 97.1(96.7, 97.4) | 74.8(74.1, 75.5) |
| Indonesia | 2017 | EAP | LM | 66.2(63.4, 69.1) | 90.0(88.6, 91.4) | 92.4(91.2, 93.7) | 91.8(90.4, 93.2) | 89.3(87.7, 91.0) | 23.1(19.8, 26.3) |
| Iraq | 2018 | MENA | UM | 87.7(86.0, 89.5) | 93.4(92.0, 94.7) | 92.9(91.1, 94.7) | 94.1(92.8, 95.5) | 93.7(91.6, 95.9) | 6.0(3.3, 8.8) |
| Kiribati | 2018 | EAP | LM | 26.7(21.7, 31.7) | 26.4(20.8, 32.0) | 44.0(37.2, 50.7) | 49.0(43.2, 54.7) | 53.6(46.0, 61.2) | 26.9(17.7, 36.1) |
| Kyrgyzstan | 2018 | ECA | LM | 92.4(89.6, 95.1) | 97.1(95.6, 98.5) | 95.2(92.7, 97.8) | 96.2(94.3, 98.0) | 98.4(97.2, 99.6) | 6.0(3.0, 9.1) |
| Lao | 2017 | EAP | LM | 30.2(27.0, 33.4) | 70.6(67.7, 73.4) | 87.0(84.9, 89.0) | 93.4(91.7, 95.0) | 96.0(94.7, 97.3) | 65.8(62.4, 69.3) |
| Lesotho | 2018 | SSA | LM | 22.5(18.1, 26.9) | 45.5(40.0, 50.9) | 59.5(53.5, 65.4) | 81.5(76.2, 86.9) | 94.2(90.4, 97.9) | 71.7(65.9, 77.4) |
| Liberia | 2019 | SSA | L | 16.3(12.3, 20.2) | 22.4(17.9, 26.9) | 25.0(20.7, 29.3) | 36.4(28.1, 44.6) | 51.0(41.8, 60.2) | 34.7(24.7, 44.8) |
| Madagascar | 2018 | SSA | L | 11.0(8.9, 13.0) | 23.8(21.0, 26.6) | 39.8(36.2, 43.3) | 58.6(54.8, 62.5) | 90.5(88.5, 92.5) | 79.6(76.7, 82.5) |
| Malawi | 2020 | SSA | L | 19.5(16.7, 22.2) | 29.0(26.4, 31.6) | 37.3(34.5, 40.1) | 50.7(47.4, 54.1) | 79.7(76.2, 83.2) | 60.2(55.7, 64.7) |
| Maldives | 2017 | SA | UM | 89.0(86.6, 91.4) | 92.2(89.5, 94.8) | 93.3(90.7, 95.8) | 96.7(94.8, 98.7) | 95.4(91.0, 99.7) | 6.3(1.4, 11.3) |
| Mali | 2018 | SSA | L | 38.6(33.8, 43.5) | 48.4(44.1, 52.6) | 54.1(50.3, 57.9) | 67.1(63.3, 70.8) | 83.0(79.8, 86.2) | 44.4(38.5, 50.2) |
| Mongolia | 2018 | EAP | LM | 86.7(83.8, 89.6) | 93.3(90.9, 95.7) | 95.1(92.8, 97.4) | 93.7(91.0, 96.3) | 96.5(94.1, 98.8) | 9.8(6.0, 13.5) |
| Myanmar | 2016 | EAP | LM | 37.1(32.4, 41.8) | 49.6(44.5, 54.8) | 62.7(57.5, 67.9) | 76.7(72.4, 80.9) | 86.0(81.8, 90.2) | 48.9(42.7, 55.2) |
| Nepal | 2019 | SA | L | 26.8(23.5, 30.1) | 51.3(45.5, 57.0) | 61.9(57.2, 66.5) | 80.3(76.2, 84.4) | 90.5(87.5, 93.4) | 63.7(59.2, 68.1) |
| Nigeria | 2018 | SSA | LM | 15.7(14.0, 17.5) | 19.6(17.8, 21.4) | 38.5(36.3, 40.7) | 58.7(56.3, 61.1) | 74.4(71.9, 76.9) | 58.7(55.6, 61.7) |
| PNG | 2016-18 | SA | LM | 6.7(4.4, 9.1) | 9.2(6.3, 12.2) | 21.3(15.3, 27.2) | 28.1(24.7, 31.5) | 64.5(56.9, 72.2) | 57.8(49.7, 65.9) |
| Pakistan | 2018 | EAP | LM | 16.3(12.4, 20.3) | 35.0(30.4, 39.6) | 54.5(49.8, 59.3) | 70.0(66.1, 73.9) | 76.9(71.8, 82.0) | 60.6(54.1, 67.1) |
| Palestine | 2019 | MENA | LM | 63.6(59.6, 67.7) | 70.4(66.1, 74.6) | 75.5(72.1, 79.0) | 83.8(81.1, 86.6) | 84.6(81.8, 87.4) | 20.9(16.0, 25.9) |
| Paraguay | 2016 | LAC | UM | 85.2(80.9, 89.5) | 96.4(94.1, 98.7) | 98.6(97.7, 99.5) | 99.1(98.6, 99.7) | 96.7(94.4, 99.1) | 11.5(6.6, 16.4) |
| Philippines | 2017 | EAP | LM | 64.1(61.0, 67.1) | 82.5(79.6, 85.3) | 89.1(86.3, 92.0) | 93.7(91.8, 95.5) | 91.8(88.9, 94.8) | 27.8(23.5, 32.1) |
| Rwanda | 2020 | SSA | L | 24.6(21.8, 27.5) | 45.3(42.2, 48.5) | 66.4(63.0, 69.8) | 78.4(75.4, 81.4) | 92.9(90.9, 94.9) | 68.3(64.8, 71.8) |
| Samoa | 2019 | EAP | L | 55.3(47.0, 63.7) | 66.6(59.8, 73.3) | 74.1(68.0, 80.2) | 75.6(69.2, 81.9) | 77.6(71.0, 84.3) | 22.3(11.9, 32.7) |
| STP | 2019 | SSA | LM | 78.9(73.5, 84.3) | 96.4(93.8, 99.0) | 96.4(93.6, 99.2) | 99.5(98.9, 100.1) | 98.1(96.1, 100.2) | 19.2(13.5, 24.9) |
| Senegal | 2019 | SSA | L | 41.7(35.5, 47.9) | 57.4(52.5, 62.2) | 80.0(76.4, 83.5) | 90.3(87.0, 93.6) | 89.2(82.2, 96.1) | 47.4(38.0, 56.8) |
| Sierra Leone | 2019 | SSA | L | 16.8(12.7, 20.8) | 17.2(14.8, 19.6) | 21.5(18.5, 24.4) | 33.1(29.0, 37.3) | 55.0(49.1, 60.9) | 38.3(31.1, 45.4) |
| South Africa | 2016 | SSA | UM | 48.2(42.5, 53.9) | 78.9(74.4, 83.3) | 91.4(88.6, 94.3) | 94.8(92.4, 97.1) | 96.4(93.7, 99.2) | 48.3(41.9, 54.6) |
| Suriname | 2018 | LAC | UM | 67.8(62.5, 73.1) | 93.7(91.6, 95.9) | 89.7(85.5, 93.9) | 93.0(89.5, 96.5) | 95.0(91.9, 98.1) | 27.2(21.0, 33.4) |
| Tajikistan | 2017 | ECA | LM | 75.5(70.2, 80.9) | 83.7(80.2, 87.3) | 86.5(83.1, 89.9) | 89.1(86.1, 92.2) | 89.9(87.2, 92.6) | 14.4(8.4, 20.3) |
| Timor-Leste | 2016 | EAP | LM | 6.3(4.5, 8.1) | 17.7(14.5, 20.9) | 34.1(29.7, 38.5) | 59.0(54.6, 63.3) | 78.0(73.5, 82.6) | 71.7(66.9, 76.6) |
| Togo | 2017 | SSA | L | 25.7(20.4, 31.0) | 34.7(30.0, 39.5) | 52.3(46.5, 58.2) | 77.6(72.6, 82.6) | 89.7(86.2, 93.2) | 64.0(57.7, 70.3) |
| Tonga* | 2019 | EAP | LM | 77.3(70.2, 84.3) | 75.1(66.9, 83.3) | 81.7(72.2, 91.1) | 85.2(76.9, 93.5) | 84.1(76.9, 91.3) | 6.9(-3.0, 16.7) |
| Tunisia | 2018 | MENA | LM | 93.4(90.6, 96.2) | 96.8(95.2, 98.4) | 95.3(93.0, 97.5) | 97.8(96.6, 99.1) | 97.1(95.2, 99.0) | 3.7(0.3, 7.1) |
| Tuvalu* | 2019 | EAP | UM | 66.0(52.3, 79.8) | 70.3(57.4, 83.2) | 79.3(66.4, 92.2) | 81.1(69.3, 92.9) | 82.3(70.9, 93.7) | 16.3(-2.1, 34.7) |
| Uganda | 2016 | SSA | L | 35.0(32.2, 37.8) | 52.9(50.4, 55.4) | 68.4(65.6, 71.3) | 73.7(71.3, 76.2) | 87.2(84.6, 89.8) | 52.2(48.4, 56.0) |
| Zambia | 2018 | SSA | LM | 13.2(11.2, 15.2) | 16.8(14.3, 19.3) | 20.2(17.6, 22.8) | 26.3(23.0, 29.6) | 39.0(34.7, 43.4) | 25.9(21.1, 30.6) |
| Zimbabwe | 2019 | SSA | L | 29.9(26.3, 33.5) | 40.9(36.8, 45.0) | 55.9(51.8, 60.0) | 79.6(76.6, 82.7) | 95.7(93.8, 97.7) | 65.8(61.7, 69.9) |

1. World Bank region group: “EAP” represents “East Asia and Pacific”; “ECA” represents “Europe and Central Asia”; “LAC” represents “Latin America and the Caribbean”; “MENA” represents “Middle East and North Africa”; “SA” represents “South Asia”; “SSA” represents “Sub-Saharan Africa”.

2. Income group: “L” represents “Low-income country”; “LM” represents “Lower-middle income country”; “UM” represents “Upper-middle income country”.

*. The disparity in prevalence between young children living in the wealthiest and poorest quintiles is not statistically significant at the 0.05 level.

*(2) Sensitivity analysis by varying threshold for adequate quarantine condition: <= two persons per room as the lower bound, and <= four persons per room as the upper-bound*

**Table S18.** Aggregate-level prevalence of young children living in households with preparedness and adequate quarantine condition (lower- and upper-bound)

|  | **No. of countries** | **Prepared households with <= two persons/room (%)** | **Prepared households with <= four persons/room (%)** | **Adequate quarantine as <= two persons/room (%)** | **Adequate quarantine as <= four persons/room (%)** |
| --- | --- | --- | --- | --- | --- |
| Average | 56 | 9.0(8.1, 10.0) | 24.1(21.0, 27.2) | 27.8(25.0, 30.5) | 82.7(80.1, 85.3) |
| Region |  |  |  |  |  |
| East Asia and Pacific | 11 | 11.2(5.6, 16.8) | 30.3(18.9, 41.7) | 25.8(16.7, 34.9) | 75.8(68.2, 83.3) |
| Europe and Central Asia | 3 | 21.5(11.3, 31.8) | 65.3(56.3, 74.4) | 29.2(16.0, 42.4) | 93.7(89.3, 98.0) |
| Latin America and the Caribbean | 5 | 19.3(7.2, 31.5) | 41.4(16.5, 66.3) | 36.0(27.0, 45.0) | 87.1(81.3, 92.9) |
| Middle East and North Africa | 4 | 21.1(11.0, 31.2) | 65.8(54.8, 76.7) | 27.4(14.1, 40.6) | 86.9(80.9, 93.0) |
| South Asia | 5 | 15.5(10.0, 21.0) | 38.9(26.1, 51.7) | 26.4(17.0, 35.8) | 80.1(68.4, 91.9) |
| Sub-Saharan Africa | 28 | 2.3(1.9, 2.7) | 5.4(4.6, 6.3) | 27.2(23.4, 31.0) | 83.3(80.2, 86.4) |
| Country income class |  |  |  |  |  |
| Low-income | 21 | 2.1(1.7, 2.6) | 5.1(4.0, 6.1) | 27.8(23.0, 32.7) | 84.1(80.5, 87.7) |
| Lower-middle income | 25 | 11.4(9.0, 13.8) | 30.5(23.5, 37.4) | 25.9(21.9, 29.9) | 80.6(76.2, 85.0) |
| Upper-middle income | 10 | 18.4(12.7, 24.1) | 48.1(32.5, 63.7) | 32.4(24.3, 40.5) | 85.3(81.1, 89.5) |

**Table S19.** Aggregate-level residential disparities in the prevalence of young children living in households with preparedness and adequate quarantine condition (lower- and upper-bound)

|  | **No. of countries** | **Prepared households with <= two persons/room (%)** | **Prepared households with <= four persons/room (%)** | **Adequate quarantine as <= two persons/room (%)** | **Adequate quarantine as <= four persons/room (%)** |
| --- | --- | --- | --- | --- | --- |
| Average | 56 | 5.3(4.5, 6.2) | 12.0(9.8, 14.2) | 5.7(4.5, 6.8) | 4.6(3.5, 5.7) |
| Region |  |  |  |  |  |
| East Asia and Pacific | 11 | 6.3(2.7, 9.9) | 17.7(11.7, 23.8) | 5.8(1.8, 9.7) | 5.7(0.8, 10.7) |
| Europe and Central Asia | 3 | 8.5(5.1, 11.8) | 20.4(-0.1, 40.9) | 7.0(3.2, 10.8) | 0.9(-0.6, 2.3) |
| Latin America and the Caribbean | 5 | 8.0(2.8, 13.3) | 14.2(6.3, 22.1) | 8.0(1.2, 14.9) | 5.8(1.5, 10.1) |
| Middle East and North Africa | 4 | 7.2(2.5, 11.9) | 11.2(5.1, 17.4) | 6.1(1.9, 10.4) | 4.0(2.5, 5.5) |
| South Asia | 5 | 8.0(4.5, 11.4) | 16.5(8.0, 24.9) | 4.3(1.3, 7.3) | 5.6(2.2, 9.0) |
| Sub-Saharan Africa | 28 | 3.5(2.8, 4.2) | 7.9(6.4, 9.3) | 5.3(3.8, 6.7) | 4.4(2.9, 5.9) |
| Country income class |  |  |  |  |  |
| Low-income | 21 | 2.8(2.2, 3.3) | 6.3(5.1, 7.4) | 6.0(4.3, 7.8) | 4.4(2.7, 6.1) |
| Lower-middle income | 25 | 6.3(4.7, 7.9) | 15.9(11.8, 20.1) | 4.6(3.0, 6.2) | 4.5(2.7, 6.4) |
| Upper-middle income | 10 | 8.9(6.1, 11.6) | 14.3(10.5, 18.2) | 7.9(4.4, 11.5) | 5.5(3.2, 7.7) |

**Table S20.** Aggregate-level wealth disparities in the prevalence of young children living in households with preparedness and adequate quarantine condition (lower- and upper-bound)

|  | **No. of countries** | **Prepared households with <= two persons/room (%)** | **Prepared households with <= four persons/room (%)** | **Adequate quarantine as <= two persons/room (%)** | **Adequate quarantine as <= four persons/room (%)** |
| --- | --- | --- | --- | --- | --- |
| Average | 56 | 19.6(16.3, 22.9) | 37.2(29.3, 45.1) | 22.1(19.1, 25.0) | 18.2(14.7, 21.7) |
| Region |  |  |  |  |  |
| East Asia and Pacific | 11 | 26.8(16.3, 37.2) | 56.6(46.3, 66.8) | 26.1(18.7, 33.6) | 28.8(18.1, 39.5) |
| Europe and Central Asia | 3 | 15.1(6.2, 24.1) | 34.4(-1.3, 70.2) | 12.4(8.7, 16.2) | 6.8(2.9, 10.8) |
| Latin America and the Caribbean | 5 | 43.1(21.0, 65.3) | 59.6(36.0, 83.2) | 43.2(28.4, 58.1) | 21.7(15.6, 27.8) |
| Middle East and North Africa | 4 | 27.9(16.2, 39.7) | 42.9(31.6, 54.1) | 26.6(14.4, 38.8) | 19.4(13.0, 25.8) |
| South Asia | 5 | 23.0(12.4, 33.5) | 53.7(41.5, 65.9) | 21.9(14.1, 29.6) | 25.2(11.3, 39.0) |
| Sub-Saharan Africa | 28 | 10.8(8.8, 12.8) | 22.1(17.8, 26.3) | 17.4(14.1, 20.7) | 13.4(10.4, 16.4) |
| Country income class |  |  |  |  |  |
| Low-income | 21 | 7.6(6.0, 9.2) | 15.7(12.7, 18.8) | 16.4(12.9, 20.0) | 13.2(9.7, 16.6) |
| Lower-middle income | 25 | 23.2(17.9, 28.5) | 47.2(37.8, 56.7) | 23.0(18.8, 27.3) | 21.3(15.1, 27.4) |
| Upper-middle income | 10 | 35.7(23.1, 48.3) | 56.8(46.3, 67.3) | 32.2(20.9, 43.5) | 21.0(16.4, 25.6) |

**Table S21.** National prevalence of young children living in households with preparedness and associated disparities by place of residence (lower-bound)

| Country | Year | WB region^1^ | Income group^2^ | National (%) | Rural (%) | Urban (%) | Difference  (urban-rural) |
| --- | --- | --- | --- | --- | --- | --- | --- |
| Algeria | 2019 | MENA | UM | 18.6(17.3, 20.0) | 13.1(11.2, 15.1) | 22.7(20.9, 24.4) | 9.6(6.9, 12.2) |
| Angola | 2016 | SSA | UM | 3.9(2.9, 4.8) | 0.5(0.1, 0.9) | 6.1(4.4, 7.7) | 5.6(3.9, 7.3) |
| Armenia | 2016 | ECA | LM | 30.4(27.2, 33.5) | 23.1(19.2, 27.0) | 35.7(31.3, 40.1) | 12.6(6.7, 18.5) |
| Bangladesh | 2019 | SA | LM | 16.2(15.6, 16.9) | 14.2(13.5, 15.0) | 23.6(21.8, 25.5) | 9.4(7.4, 11.4) |
| Benin | 2018 | SSA | L | 0.6(0.4, 0.8) | 0.1(0.0, 0.2) | 1.3(0.8, 1.8) | 1.1(0.6, 1.6) |
| Burundi | 2017 | SSA | L | 0.5(0.4, 0.7) | 0.3(0.2, 0.5) | 2.8(1.4, 4.2) | 2.5(1.0, 3.9) |
| CAR | 2018 | SSA | L | 0.5(0.3, 0.8) | 0.1(-0.1, 0.3) | 1.6(1.0, 2.1) | 1.5(0.8, 2.1) |
| Cameroon | 2018 | SSA | LM | 4.8(4.0, 5.5) | 1.5(0.8, 2.2) | 8.8(7.4, 10.2) | 7.3(5.7, 8.9) |
| Chad | 2019 | SSA | L | 0.9(0.7, 1.2) | 0.1(0.0, 0.2) | 5.2(3.6, 6.7) | 5.0(3.5, 6.6) |
| Congo DR | 2018 | SSA | L | 0.5(0.3, 0.7) | 0.0(0.0, 0.0) | 1.3(0.8, 1.9) | 1.3(0.8, 1.9) |
| Cote d’Ivoire | 2016 | SSA | LM | 1.6(1.1, 2.1) | 0.3(0.1, 0.4) | 3.7(2.3, 5.1) | 3.4(2.0, 4.8) |
| Dominican Republic | 2019 | LAC | UM | 20.8(19.2, 22.4) | 13.9(11.8, 15.9) | 23.3(21.2, 25.3) | 9.4(6.5, 12.3) |
| Ethiopia | 2016 | SSA | L | 0.3(0.2, 0.4) | 0.0(0.0, 0.0) | 2.4(1.4, 3.5) | 2.4(1.4, 3.5) |
| Gambia | 2020 | SSA | L | 2.0(1.4, 2.5) | 0.8(0.3, 1.3) | 2.6(1.7, 3.4) | 1.8(0.8, 2.8) |
| Ghana | 2017 | SSA | LM | 3.1(2.3, 3.8) | 1.2(0.7, 1.7) | 5.5(3.9, 7.1) | 4.3(2.6, 6.0) |
| Guinea | 2018 | SSA | L | 1.3(0.9, 1.7) | 0.3(0.1, 0.5) | 3.9(2.6, 5.1) | 3.6(2.3, 4.8) |
| Guinea-Bissau | 2019 | SSA | L | 0.7(0.4, 1.0) | 0.2(0.0, 0.4) | 2.0(1.1, 2.9) | 1.8(0.8, 2.7) |
| Guyana* | 2019 | LAC | UM | 25.6(22.2, 29.0) | 25.7(21.7, 29.8) | 25.2(19.6, 30.8) | -0.5(-7.4, 6.3) |
| Haiti | 2017 | LAC | L | 2.4(1.8, 3.0) | 1.4(0.8, 2.1) | 4.1(2.8, 5.4) | 2.7(1.2, 4.1) |
| India | 2016 | SA | LM | 7.4(7.2, 7.6) | 4.8(4.6, 5.0) | 14.0(13.4, 14.7) | 9.2(8.5, 9.9) |
| Indonesia | 2017 | EAP | LM | 34.4(33.2, 35.7) | 31.7(30.0, 33.5) | 37.3(35.5, 39.0) | 5.5(3.0, 8.0) |
| Iraq | 2018 | MENA | UM | 9.5(8.5, 10.6) | 6.9(5.6, 8.2) | 10.8(9.4, 12.2) | 3.9(2.0, 5.8) |
| Kiribati* | 2018 | EAP | LM | 2.0(1.2, 2.8) | 1.6(0.7, 2.6) | 2.3(1.1, 3.6) | 0.7(-0.9, 2.2) |
| Kyrgyzstan | 2018 | ECA | LM | 22.5(20.4, 24.5) | 20.7(18.3, 23.1) | 26.3(22.6, 29.9) | 5.6(1.1, 10.0) |
| Lao | 2017 | EAP | LM | 10.9(10.0, 11.8) | 6.0(5.1, 6.9) | 23.9(21.6, 26.2) | 17.9(15.4, 20.3) |
| Lesotho | 2018 | SSA | LM | 2.1(1.3, 2.8) | 1.0(0.3, 1.6) | 4.3(2.5, 6.1) | 3.3(1.4, 5.2) |
| Liberia | 2019 | SSA | L | 0.4(0.1, 0.8) | 0.0(0.0, 0.0) | 0.8(0.1, 1.4) | 0.8(0.1, 1.4) |
| Madagascar | 2018 | SSA | L | 0.9(0.6, 1.1) | 0.4(0.2, 0.6) | 2.7(1.9, 3.6) | 2.3(1.5, 3.2) |
| Malawi | 2020 | SSA | L | 2.6(2.1, 3.2) | 1.7(1.4, 2.1) | 8.8(5.7, 12.0) | 7.1(3.9, 10.2) |
| Maldives | 2017 | SA | UM | 32.3(29.7, 34.8) | 37.9(35.5, 40.2) | 22.0(16.3, 27.8) | -15.8(-22.1, -9.6) |
| Mali | 2018 | SSA | L | 2.0(1.5, 2.4) | 1.3(0.9, 1.7) | 4.4(2.9, 5.9) | 3.0(1.5, 4.6) |
| Mongolia | 2018 | EAP | LM | 11.2(9.4, 13.0) | 5.7(4.0, 7.4) | 13.9(11.3, 16.4) | 8.1(5.1, 11.2) |
| Myanmar | 2016 | EAP | LM | 5.2(4.1, 6.4) | 3.3(2.1, 4.6) | 11.9(8.9, 14.9) | 8.6(5.3, 11.9) |
| Nepal | 2019 | SA | L | 18.4(16.5, 20.3) | 14.9(12.7, 17.1) | 20.3(17.6, 23.0) | 5.4(1.9, 8.9) |
| Nigeria | 2018 | SSA | LM | 3.2(2.8, 3.7) | 1.2(1.0, 1.4) | 6.4(5.4, 7.5) | 5.2(4.1, 6.3) |
| PNG | 2016-18 | SA | LM | 3.3(0.7, 5.8) | 2.6(-0.2, 5.5) | 8.6(5.9, 11.4) | 6.0(2.0, 10.0) |
| Pakistan | 2018 | EAP | LM | 3.7(3.0, 4.4) | 2.8(1.9, 3.7) | 5.6(4.4, 6.8) | 2.8(1.3, 4.3) |
| Palestine* | 2019 | MENA | LM | 14.8(13.4, 16.2) | 16.1(13.9, 18.3) | 14.4(12.7, 16.0) | -1.7(-4.5, 1.0) |
| Paraguay | 2016 | LAC | UM | 27.9(25.5, 30.3) | 19.8(17.0, 22.7) | 33.6(29.9, 37.2) | 13.7(9.1, 18.4) |
| Philippines* | 2017 | EAP | LM | 10.1(9.0, 11.1) | 9.4(8.2, 10.6) | 10.9(9.1, 12.6) | 1.5(-0.6, 3.6) |
| Rwanda | 2020 | SSA | L | 7.3(6.3, 8.3) | 6.0(5.1, 6.9) | 13.6(10.2, 17.0) | 7.6(4.1, 11.1) |
| Samoa | 2019 | EAP | L | 7.9(6.4, 9.4) | 6.7(5.1, 8.2) | 14.2(10.0, 18.4) | 7.5(3.0, 12.0) |
| STP | 2019 | SSA | LM | 10.3(8.4, 12.1) | 7.5(5.1, 9.9) | 11.7(9.1, 14.2) | 4.1(0.6, 7.7) |
| Senegal | 2019 | SSA | L | 3.5(2.3, 4.7) | 1.9(1.3, 2.5) | 6.3(3.1, 9.5) | 4.4(1.2, 7.7) |
| Sierra Leone | 2019 | SSA | L | 0.8(0.5, 1.0) | 0.2(0.1, 0.4) | 1.7(1.0, 2.4) | 1.5(0.8, 2.1) |
| South Africa | 2016 | SSA | UM | 15.2(12.7, 17.7) | 6.1(4.5, 7.7) | 20.4(16.6, 24.1) | 14.2(10.2, 18.3) |
| Suriname | 2018 | LAC | UM | 20.2(17.9, 22.5) | 11.8(9.4, 14.1) | 24.9(21.7, 28.2) | 13.2(9.1, 17.2) |
| Tajikistan | 2017 | ECA | LM | 12.0(10.7, 13.4) | 10.3(8.7, 11.8) | 18.8(16.0, 21.6) | 8.5(5.4, 11.7) |
| Timor-Leste | 2016 | EAP | LM | 5.8(4.5, 7.0) | 3.2(1.8, 4.5) | 12.3(9.6, 14.9) | 9.1(6.1, 12.1) |
| Togo | 2017 | SSA | L | 2.2(1.5, 2.9) | 0.5(0.2, 0.8) | 4.9(3.3, 6.5) | 4.4(2.8, 6.1) |
| Tonga* | 2019 | EAP | LM | 22.3(18.5, 26.2) | 22.1(17.5, 26.8) | 23.1(17.4, 28.8) | 1.0(-6.4, 8.5) |
| Tunisia | 2018 | MENA | LM | 41.7(39.3, 44.1) | 32.2(28.7, 35.8) | 47.0(44.1, 49.9) | 14.8(10.2, 19.4) |
| Tuvalu* | 2019 | EAP | UM | 10.6(7.2, 14.0) | 11.6(5.8, 17.4) | 10.1(5.9, 14.3) | -1.5(-8.8, 5.8) |
| Uganda | 2016 | SSA | L | 1.8(1.4, 2.1) | 0.9(0.6, 1.1) | 5.1(3.6, 6.6) | 4.2(2.7, 5.7) |
| Zambia | 2018 | SSA | LM | 0.8(0.5, 1.2) | 0.2(0.1, 0.3) | 2.0(1.1, 3.0) | 1.9(0.9, 2.8) |
| Zimbabwe | 2019 | SSA | L | 5.3(4.3, 6.2) | 3.5(2.6, 4.4) | 9.6(7.1, 12.0) | 6.1(3.5, 8.6) |

**Note:**

1. World Bank region group: “EAP” represents “East Asia and Pacific”; “ECA” represents “Europe and Central Asia”; “LAC” represents “Latin America and the Caribbean”; “MENA” represents “Middle East and North Africa”; “SA” represents “South Asia”; “SSA” represents “Sub-Saharan Africa”.

2. Income group: “L” represents “Low-income country”; “LM” represents “Lower-middle income country”; “UM” represents “Upper-middle income country”.

*. The disparity in prevalence between young children in urban and rural areas is not statistically significant at the 0.05 level.

**Table S22.** National prevalence of young children living in households with preparedness and associated disparities by household wealth quintile (lower-bound)

| **Country** | **Year** | **WB region^1^** | **Income group^2^** | **Wealth quintile** | | | | | **Difference (richest-poorest)** |
| --- | --- | --- | --- | --- | --- | --- | --- | --- | --- |
|  |  |  |  | **Poorest (%)** | **2 (%)** | **3 (%)** | **4 (%)** | **Richest (%)** |  |
| Algeria | 2019 | MENA | UM | 5.8(4.5, 7.0) | 10.7(9.1, 12.3) | 18.8(16.6, 21.1) | 28.5(25.4, 31.7) | 39.4(36.1, 42.7) | 33.6(30.1, 37.1) |
| Angola | 2016 | SSA | UM | 0.0(0.0, 0.0) | 0.3(0.0, 0.6) | 1.6(1.0, 2.2) | 4.1(2.4, 5.8) | 19.4(14.1, 24.7) | 19.4(14.1, 24.7) |
| Armenia | 2016 | ECA | LM | 13.7(9.3, 18.0) | 28.8(22.9, 34.8) | 35.3(28.6, 42.0) | 32.0(25.3, 38.7) | 40.0(32.5, 47.5) | 26.3(17.5, 35.1) |
| Bangladesh | 2019 | SA | LM | 0.7(0.4, 0.9) | 3.8(3.2, 4.4) | 12.3(11.1, 13.4) | 24.2(22.5, 25.8) | 40.6(38.5, 42.7) | 39.9(37.8, 42.0) |
| Benin | 2018 | SSA | L | 0.0(0.0, 0.0) | 0.0(0.0, 0.0) | 0.0(0.0, 0.0) | 0.1(0.0, 0.3) | 3.1(2.0, 4.1) | 3.1(2.0, 4.1) |
| Burundi | 2017 | SSA | L | 0.0(0.0, 0.0) | 0.1(0.0, 0.2) | 0.0(0.0, 0.0) | 0.5(0.1, 0.9) | 2.6(1.6, 3.5) | 2.6(1.6, 3.5) |
| CAR | 2018 | SSA | L | 0.0(0.0, 0.0) | 0.0(0.0, 0.0) | 0.0(0.0, 0.0) | 0.5(-0.3, 1.3) | 3.1(2.0, 4.2) | 3.1(2.0, 4.2) |
| Cameroon | 2018 | SSA | LM | 0.0(0.0, 0.0) | 0.6(0.1, 1.1) | 1.9(1.0, 2.8) | 4.4(3.2, 5.7) | 22.3(19.1, 25.5) | 22.3(19.1, 25.5) |
| Chad | 2019 | SSA | L | 0.0(0.0, 0.0) | 0.0(0.0, 0.0) | 0.0(0.0, 0.0) | 0.1(0.0, 0.3) | 5.5(4.0, 7.1) | 5.5(4.0, 7.1) |
| Congo DR | 2018 | SSA | L | 0.0(0.0, 0.0) | 0.0(0.0, 0.0) | 0.0(0.0, 0.1) | 0.3(0.0, 0.7) | 2.9(1.5, 4.3) | 2.9(1.5, 4.3) |
| Cote d’Ivoire | 2016 | SSA | LM | 0.0(0.0, 0.0) | 0.1(0.0, 0.2) | 0.6(0.1, 1.0) | 1.1(0.4, 1.7) | 9.2(5.9, 12.4) | 9.2(5.9, 12.4) |
| Dominican Republic | 2019 | LAC | UM | 1.7(1.1, 2.3) | 8.2(6.6, 9.9) | 19.4(16.7, 22.2) | 36.5(31.6, 41.3) | 53.4(48.4, 58.4) | 51.7(46.7, 56.7) |
| Ethiopia | 2016 | SSA | L | 0.0(0.0, 0.0) | 0.0(0.0, 0.0) | 0.0(0.0, 0.0) | 0.0(0.0, 0.0) | 1.9(1.1, 2.7) | 1.9(1.1, 2.7) |
| Gambia | 2020 | SSA | L | 0.3(0.1, 0.5) | 0.6(0.0, 1.1) | 0.3(-0.1, 0.7) | 3.3(1.5, 5.2) | 6.5(4.2, 8.8) | 6.2(3.9, 8.5) |
| Ghana | 2017 | SSA | LM | 0.1(0.0, 0.3) | 0.2(0.0, 0.3) | 1.0(0.2, 1.9) | 0.6(0.2, 1.1) | 14.6(11.4, 17.9) | 14.5(11.2, 17.8) |
| Guinea | 2018 | SSA | L | 0.0(0.0, 0.0) | 0.1(-0.1, 0.4) | 0.5(0.0, 0.9) | 1.1(0.4, 1.8) | 6.6(4.5, 8.8) | 6.6(4.5, 8.8) |
| Guinea-Bissau | 2019 | SSA | L | 0.0(0.0, 0.0) | 0.0(0.0, 0.0) | 0.0(0.0, 0.0) | 1.1(0.3, 1.9) | 3.7(2.0, 5.3) | 3.7(2.0, 5.3) |
| Guyana | 2019 | LAC | UM | 6.5(4.3, 8.8) | 20.2(15.3, 25.1) | 29.3(21.0, 37.6) | 37.6(29.2, 46.0) | 51.7(43.5, 59.9) | 45.2(36.7, 53.6) |
| Haiti | 2017 | LAC | L | 0.0(0.0, 0.0) | 0.3(-0.2, 0.9) | 0.9(0.1, 1.7) | 1.8(0.7, 3.0) | 12.3(9.1, 15.6) | 12.3(9.1, 15.6) |
| India | 2016 | SA | LM | 0.0(0.0, 0.0) | 0.8(0.7, 0.9) | 4.0(3.7, 4.3) | 11.9(11.3, 12.4) | 29.0(28.0, 30.0) | 29.0(27.9, 30.0) |
| Indonesia | 2017 | EAP | LM | 6.6(5.6, 7.7) | 23.7(21.6, 25.8) | 36.7(34.3, 39.0) | 47.5(45.1, 49.9) | 59.7(57.1, 62.4) | 53.1(50.3, 55.9) |
| Iraq | 2018 | MENA | UM | 4.0(2.6, 5.4) | 5.4(4.2, 6.6) | 8.7(6.9, 10.4) | 15.9(12.9, 18.9) | 16.4(13.8, 19.1) | 12.5(9.5, 15.4) |
| Kiribati | 2018 | EAP | LM | 0.0(0.0, 0.0) | 2.2(0.6, 3.9) | 1.2(0.0, 2.3) | 0.9(-0.1, 1.9) | 6.3(3.0, 9.6) | 6.3(2.9, 9.7) |
| Kyrgyzstan* | 2018 | ECA | LM | 21.5(17.2, 25.8) | 15.7(11.6, 19.8) | 25.0(20.1, 29.8) | 24.6(20.1, 29.2) | 28.2(23.0, 33.5) | 6.7(-0.2, 13.6) |
| Lao | 2017 | EAP | LM | 0.0(0.0, 0.1) | 2.2(1.5, 2.9) | 6.4(5.0, 7.8) | 16.1(14.1, 18.2) | 39.0(35.8, 42.2) | 38.9(35.7, 42.1) |
| Lesotho | 2018 | SSA | LM | 0.0(0.0, 0.0) | 0.0(0.0, 0.1) | 0.5(-0.3, 1.3) | 0.4(-0.1, 0.8) | 11.6(7.3, 15.9) | 11.6(7.3, 15.9) |
| Liberia | 2019 | SSA | L | 0.0(0.0, 0.0) | 0.0(0.0, 0.0) | 0.0(0.0, 0.0) | 0.0(0.0, 0.0) | 2.5(0.4, 4.6) | 2.5(0.4, 4.6) |
| Madagascar | 2018 | SSA | L | 0.0(0.0, 0.0) | 0.0(0.0, 0.0) | 0.0(0.0, 0.1) | 0.6(0.2, 1.0) | 5.1(3.7, 6.5) | 5.1(3.7, 6.5) |
| Malawi | 2020 | SSA | L | 0.0(0.0, 0.1) | 0.1(0.0, 0.3) | 1.4(0.9, 2.0) | 2.6(1.4, 3.8) | 12.2(9.5, 14.8) | 12.1(9.4, 14.8) |
| Maldives* | 2017 | SA | UM | 32.9(29.5, 36.4) | 36.6(32.0, 41.1) | 37.2(32.5, 41.8) | 24.5(17.0, 32.0) | 27.8(18.1, 37.5) | -5.2(-15.5, 5.1) |
| Mali | 2018 | SSA | L | 0.2(-0.1, 0.5) | 0.8(0.3, 1.4) | 1.3(0.5, 2.1) | 1.8(1.0, 2.7) | 6.6(4.8, 8.5) | 6.4(4.6, 8.3) |
| Mongolia | 2018 | EAP | LM | 0.5(0.1, 0.9) | 1.6(0.7, 2.5) | 4.5(2.9, 6.1) | 19.1(14.8, 23.5) | 26.0(21.2, 30.8) | 25.5(20.7, 30.4) |
| Myanmar | 2016 | EAP | LM | 0.6(-0.2, 1.3) | 1.4(0.3, 2.4) | 2.8(1.3, 4.4) | 7.1(4.7, 9.5) | 21.9(15.7, 28.1) | 21.3(15.1, 27.6) |
| Nepal | 2019 | SA | L | 5.4(4.1, 6.7) | 13.7(10.9, 16.5) | 18.7(15.7, 21.8) | 25.5(22.2, 28.9) | 33.9(27.4, 40.5) | 28.5(21.8, 35.2) |
| Nigeria | 2018 | SSA | LM | 0.0(0.0, 0.0) | 0.1(0.0, 0.3) | 0.6(0.4, 0.9) | 4.1(3.3, 5.0) | 14.2(12.2, 16.2) | 14.2(12.2, 16.2) |
| PNG | 2016-18 | SA | LM | 0.0(0.0, 0.0) | 0.1(-0.1, 0.3) | 0.1(0.0, 0.2) | 1.0(0.4, 1.5) | 17.4(5.0, 29.7) | 17.4(5.0, 29.8) |
| Pakistan | 2018 | EAP | LM | 0.0(0.0, 0.0) | 0.3(0.0, 0.6) | 2.5(1.2, 3.7) | 7.6(5.0, 10.2) | 9.7(7.1, 12.2) | 9.7(7.1, 12.2) |
| Palestine | 2019 | MENA | LM | 3.1(1.8, 4.5) | 7.9(5.4, 10.4) | 15.2(11.9, 18.6) | 19.6(16.4, 22.7) | 31.5(27.9, 35.2) | 28.4(24.5, 32.3) |
| Paraguay | 2016 | LAC | UM | 2.8(1.8, 3.9) | 16.7(13.4, 20.0) | 33.7(28.7, 38.6) | 42.7(37.5, 47.9) | 65.7(59.5, 71.8) | 62.8(56.5, 69.1) |
| Philippines | 2017 | EAP | LM | 1.5(1.0, 2.0) | 4.2(3.2, 5.3) | 9.7(7.7, 11.7) | 15.1(11.9, 18.2) | 32.7(27.4, 38.1) | 31.2(25.9, 36.6) |
| Rwanda | 2020 | SSA | L | 0.2(0.0, 0.5) | 1.9(1.1, 2.8) | 6.0(4.6, 7.3) | 8.3(6.1, 10.4) | 22.6(18.9, 26.3) | 22.4(18.7, 26.1) |
| Samoa | 2019 | EAP | L | 0.6(0.0, 1.2) | 2.3(0.6, 4.0) | 6.3(3.7, 8.9) | 13.6(9.2, 17.9) | 22.9(16.7, 29.0) | 22.3(16.1, 28.5) |
| STP | 2019 | SSA | LM | 0.2(-0.2, 0.5) | 1.7(-0.1, 3.5) | 4.0(1.9, 6.2) | 16.2(11.8, 20.7) | 38.9(31.9, 46.0) | 38.8(31.6, 45.9) |
| Senegal | 2019 | SSA | L | 1.0(0.2, 1.7) | 1.9(1.0, 2.8) | 1.4(0.6, 2.3) | 2.0(0.9, 3.0) | 13.5(7.8, 19.2) | 12.5(6.7, 18.3) |
| Sierra Leone | 2019 | SSA | L | 0.0(0.0, 0.0) | 0.0(0.0, 0.1) | 0.3(0.0, 0.7) | 0.5(0.2, 0.9) | 3.8(2.3, 5.3) | 3.8(2.3, 5.3) |
| South Africa | 2016 | SSA | UM | 1.0(0.4, 1.7) | 3.5(2.1, 5.0) | 7.9(5.3, 10.5) | 21.4(16.1, 26.7) | 63.9(56.1, 71.6) | 62.8(55.0, 70.6) |
| Suriname | 2018 | LAC | UM | 4.2(2.2, 6.2) | 18.3(14.4, 22.1) | 22.7(17.3, 28.1) | 34.8(29.0, 40.6) | 48.1(39.8, 56.3) | 43.9(35.4, 52.4) |
| Tajikistan | 2017 | ECA | LM | 7.9(5.7, 10.1) | 8.6(6.3, 10.9) | 10.5(8.1, 13.0) | 13.0(10.1, 15.9) | 21.7(18.4, 24.9) | 13.8(9.8, 17.8) |
| Timor-Leste | 2016 | EAP | LM | 0.0(0.0, 0.0) | 0.4(0.0, 0.9) | 2.0(1.0, 3.0) | 6.3(2.4, 10.2) | 20.7(16.9, 24.4) | 20.7(16.9, 24.5) |
| Togo | 2017 | SSA | L | 0.0(0.0, 0.0) | 0.0(0.0, 0.1) | 0.4(-0.2, 0.9) | 0.6(-0.1, 1.3) | 11.2(8.0, 14.5) | 11.2(8.0, 14.5) |
| Tonga | 2019 | EAP | LM | 5.0(2.2, 7.9) | 12.3(6.9, 17.7) | 26.4(17.7, 35.2) | 33.9(24.7, 43.1) | 43.3(32.5, 54.0) | 38.2(27.0, 49.4) |
| Tunisia | 2018 | MENA | LM | 22.4(18.3, 26.4) | 34.9(30.3, 39.5) | 42.7(37.5, 47.8) | 49.9(44.8, 54.9) | 60.3(54.8, 65.9) | 37.9(31.0, 44.8) |
| Tuvalu | 2019 | EAP | UM | 5.8(1.2, 10.3) | 5.5(-0.3, 11.3) | 9.8(1.4, 18.3) | 10.7(3.5, 17.9) | 23.8(10.4, 37.3) | 18.1(3.2, 32.9) |
| Uganda | 2016 | SSA | L | 0.0(0.0, 0.0) | 0.0(0.0, 0.0) | 0.3(0.1, 0.5) | 0.9(0.4, 1.3) | 8.1(6.3, 9.9) | 8.1(6.3, 9.9) |
| Zambia | 2018 | SSA | LM | 0.0(0.0, 0.0) | 0.0(0.0, 0.0) | 0.0(0.0, 0.1) | 0.4(0.0, 0.9) | 4.8(2.7, 6.9) | 4.8(2.7, 6.9) |
| Zimbabwe | 2019 | SSA | L | 0.2(-0.1, 0.4) | 1.5(0.8, 2.3) | 5.6(4.0, 7.2) | 4.8(3.2, 6.4) | 17.7(13.6, 21.9) | 17.6(13.4, 21.7) |

**Note:**

1. World Bank region group: “EAP” represents “East Asia and Pacific”; “ECA” represents “Europe and Central Asia”; “LAC” represents “Latin America and the Caribbean”; “MENA” represents “Middle East and North Africa”; “SA” represents “South Asia”; “SSA” represents “Sub-Saharan Africa”.

2. Income group: “L” represents “Low-income country”; “LM” represents “Lower-middle income country”; “UM” represents “Upper-middle income country”.

*. The disparity in prevalence between young children living in the wealthiest and poorest quintiles is not statistically significant at the 0.05 level.

**Table S23.** National prevalence of young children living in households with preparedness and associated disparities by place of residence (upper-bound)

| Country | Year | WB region^1^ | Income group^2^ | National (%) | Rural (%) | Urban (%) | Difference  (urban-rural) |
| --- | --- | --- | --- | --- | --- | --- | --- |
| Algeria | 2019 | MENA | UM | 55.4(53.2, 57.5) | 46.9(43.1, 50.6) | 61.7(59.3, 64.0) | 14.8(10.4, 19.2) |
| Angola | 2016 | SSA | UM | 9.6(8.0, 11.2) | 1.1(0.2, 1.9) | 15.4(12.7, 18.0) | 14.3(11.5, 17.1) |
| Armenia | 2016 | ECA | LM | 71.0(67.6, 74.4) | 48.0(42.5, 53.6) | 88.3(85.2, 91.5) | 40.3(33.9, 46.7) |
| Bangladesh | 2019 | SA | LM | 31.2(30.3, 32.2) | 27.4(26.4, 28.3) | 45.6(43.0, 48.1) | 18.2(15.5, 21.0) |
| Benin | 2018 | SSA | L | 1.7(1.3, 2.0) | 0.5(0.2, 0.7) | 3.5(2.6, 4.5) | 3.1(2.1, 4.0) |
| Burundi | 2017 | SSA | L | 1.4(1.0, 1.8) | 0.7(0.5, 1.0) | 8.1(5.1, 11.1) | 7.4(4.4, 10.3) |
| CAR | 2018 | SSA | L | 1.0(0.7, 1.3) | 0.1(-0.1, 0.4) | 3.1(2.3, 4.0) | 3.0(2.1, 3.9) |
| Cameroon | 2018 | SSA | LM | 10.4(9.0, 11.7) | 2.4(1.3, 3.5) | 20.2(17.6, 22.7) | 17.8(15.0, 20.5) |
| Chad | 2019 | SSA | L | 2.0(1.6, 2.4) | 0.3(0.2, 0.5) | 11.0(8.7, 13.2) | 10.7(8.4, 12.9) |
| Congo DR | 2018 | SSA | L | 1.8(1.2, 2.3) | 0.0(0.0, 0.0) | 4.5(3.1, 5.9) | 4.5(3.1, 5.9) |
| Cote d’Ivoire | 2016 | SSA | LM | 6.1(4.8, 7.4) | 1.1(0.7, 1.5) | 14.2(11.0, 17.3) | 13.1(9.9, 16.3) |
| Dominican Republic | 2019 | LAC | UM | 38.2(36.1, 40.2) | 25.8(22.9, 28.7) | 42.7(40.1, 45.2) | 16.9(13.0, 20.7) |
| Ethiopia | 2016 | SSA | L | 0.7(0.3, 1.0) | 0.0(0.0, 0.0) | 6.1(3.3, 8.9) | 6.1(3.3, 8.9) |
| Gambia | 2020 | SSA | L | 4.3(3.2, 5.3) | 1.7(0.9, 2.5) | 5.6(4.1, 7.2) | 4.0(2.2, 5.7) |
| Ghana | 2017 | SSA | LM | 8.3(6.8, 9.8) | 4.8(2.9, 6.7) | 13.0(10.6, 15.4) | 8.2(5.1, 11.2) |
| Guinea | 2018 | SSA | L | 3.1(2.4, 3.8) | 0.9(0.5, 1.4) | 8.7(6.6, 10.7) | 7.7(5.6, 9.8) |
| Guinea-Bissau | 2019 | SSA | L | 1.8(1.3, 2.3) | 0.6(0.2, 1.0) | 5.3(3.6, 7.0) | 4.8(3.0, 6.5) |
| Guyana* | 2019 | LAC | UM | 53.8(48.8, 58.9) | 53.6(47.4, 59.7) | 54.8(47.0, 62.7) | 1.3(-8.7, 11.2) |
| Haiti | 2017 | LAC | L | 5.0(4.0, 6.1) | 2.8(1.8, 3.8) | 9.2(6.9, 11.4) | 6.4(3.9, 8.8) |
| India | 2016 | SA | LM | 22.5(22.1, 22.9) | 14.7(14.4, 15.0) | 42.7(41.5, 43.8) | 27.9(26.8, 29.1) |
| Indonesia | 2017 | EAP | LM | 56.9(55.4, 58.4) | 49.0(46.9, 51.2) | 65.2(63.2, 67.2) | 16.2(13.2, 19.1) |
| Iraq | 2018 | MENA | UM | 63.6(61.6, 65.6) | 55.0(50.7, 59.2) | 67.7(65.2, 70.1) | 12.7(7.8, 17.6) |
| Kiribati | 2018 | EAP | LM | 10.3(8.0, 12.5) | 5.2(3.4, 7.0) | 14.8(11.0, 18.6) | 9.6(5.3, 13.8) |
| Kyrgyzstan | 2018 | ECA | LM | 69.5(67.1, 71.9) | 71.3(68.5, 74.0) | 65.8(61.4, 70.2) | -5.5(-10.7, -0.2) |
| Lao | 2017 | EAP | LM | 29.3(27.7, 30.9) | 19.6(17.9, 21.2) | 55.6(52.3, 58.9) | 36.0(32.4, 39.7) |
| Lesotho | 2018 | SSA | LM | 4.7(3.5, 5.8) | 2.6(1.5, 3.7) | 8.9(6.1, 11.6) | 6.2(3.3, 9.2) |
| Liberia | 2019 | SSA | L | 1.0(0.5, 1.6) | 0.0(0.0, 0.0) | 1.9(0.9, 3.0) | 1.9(0.9, 3.0) |
| Madagascar | 2018 | SSA | L | 2.4(2.0, 2.8) | 1.4(1.0, 1.7) | 6.6(5.2, 8.0) | 5.3(3.8, 6.7) |
| Malawi | 2020 | SSA | L | 5.8(5.1, 6.6) | 4.3(3.7, 4.9) | 16.4(11.8, 21.1) | 12.2(7.4, 16.9) |
| Maldives* | 2017 | SA | UM | 82.1(79.4, 84.7) | 82.5(80.4, 84.7) | 81.3(74.9, 87.7) | -1.2(-8.0, 5.5) |
| Mali | 2018 | SSA | L | 4.0(3.3, 4.7) | 2.7(2.1, 3.3) | 9.1(6.7, 11.5) | 6.4(3.9, 8.9) |
| Mongolia | 2018 | EAP | LM | 39.9(35.5, 44.2) | 18.8(15.4, 22.3) | 50.0(43.9, 56.0) | 31.1(24.2, 38.1) |
| Myanmar | 2016 | EAP | LM | 16.3(14.4, 18.1) | 11.0(9.1, 12.9) | 34.6(30.0, 39.3) | 23.6(18.5, 28.6) |
| Nepal | 2019 | SA | L | 36.7(34.3, 39.1) | 30.9(27.5, 34.4) | 39.9(36.7, 43.1) | 8.9(4.2, 13.7) |
| Nigeria | 2018 | SSA | LM | 6.7(6.0, 7.4) | 2.7(2.2, 3.1) | 13.2(11.5, 15.0) | 10.6(8.8, 12.4) |
| PNG | 2016-18 | SA | LM | 5.6(3.0, 8.2) | 3.7(0.9, 6.6) | 22.0(16.6, 27.3) | 18.3(12.2, 24.3) |
| Pakistan | 2018 | EAP | LM | 22.1(19.7, 24.6) | 14.4(11.6, 17.2) | 38.8(34.9, 42.7) | 24.4(19.6, 29.3) |
| Palestine* | 2019 | MENA | LM | 62.2(60.1, 64.3) | 64.2(61.0, 67.3) | 61.6(59.0, 64.2) | -2.6(-6.7, 1.5) |
| Paraguay | 2016 | LAC | UM | 61.9(58.9, 64.8) | 47.4(43.4, 51.4) | 72.3(68.1, 76.4) | 24.9(19.1, 30.6) |
| Philippines | 2017 | EAP | LM | 39.3(37.1, 41.5) | 35.9(33.0, 38.9) | 43.5(40.0, 47.0) | 7.6(2.9, 12.2) |
| Rwanda | 2020 | SSA | L | 10.7(9.6, 11.8) | 9.4(8.3, 10.5) | 17.0(13.2, 20.7) | 7.6(3.7, 11.5) |
| Samoa | 2019 | EAP | L | 30.8(27.2, 34.4) | 28.0(24.1, 32.0) | 45.3(39.3, 51.3) | 17.2(10.0, 24.5) |
| STP* | 2019 | SSA | LM | 19.8(16.9, 22.7) | 16.8(12.8, 20.7) | 21.3(17.4, 25.2) | 4.5(-1.1, 10.1) |
| Senegal | 2019 | SSA | L | 7.1(5.4, 8.9) | 3.8(2.7, 4.8) | 13.2(8.3, 18.1) | 9.4(4.4, 14.5) |
| Sierra Leone | 2019 | SSA | L | 1.5(1.1, 1.9) | 0.4(0.2, 0.7) | 3.4(2.4, 4.5) | 3.0(1.9, 4.1) |
| South Africa | 2016 | SSA | UM | 23.8(21.0, 26.6) | 10.0(7.7, 12.3) | 31.7(27.4, 36.0) | 21.7(16.8, 26.5) |
| Suriname | 2018 | LAC | UM | 48.3(45.1, 51.5) | 35.6(31.3, 39.9) | 55.7(51.4, 60.0) | 20.1(14.0, 26.2) |
| Tajikistan | 2017 | ECA | LM | 55.4(51.9, 58.8) | 52.1(47.9, 56.3) | 67.6(62.6, 72.6) | 15.5(8.9, 22.1) |
| Timor-Leste | 2016 | EAP | LM | 10.9(9.4, 12.4) | 5.1(3.6, 6.6) | 25.4(21.5, 29.3) | 20.3(16.1, 24.5) |
| Togo | 2017 | SSA | L | 4.7(3.4, 6.0) | 0.9(0.3, 1.4) | 10.8(7.9, 13.8) | 10.0(7.0, 13.0) |
| Tonga* | 2019 | EAP | LM | 50.2(45.3, 55.0) | 49.3(43.6, 55.0) | 53.4(44.2, 62.6) | 4.1(-6.8, 15.0) |
| Tunisia | 2018 | MENA | LM | 81.9(79.7, 84.0) | 72.2(68.2, 76.2) | 87.3(85.2, 89.4) | 15.1(10.6, 19.6) |
| Tuvalu* | 2019 | EAP | UM | 44.6(37.8, 51.4) | 40.4(33.4, 47.5) | 46.7(37.3, 56.1) | 6.2(-5.8, 18.2) |
| Uganda | 2016 | SSA | L | 3.9(3.3, 4.5) | 2.3(1.7, 2.8) | 10.1(8.1, 12.0) | 7.8(5.7, 9.8) |
| Zambia | 2018 | SSA | LM | 2.2(1.6, 2.8) | 0.5(0.3, 0.8) | 5.3(3.6, 6.9) | 4.7(3.0, 6.4) |
| Zimbabwe | 2019 | SSA | L | 13.0(11.5, 14.6) | 9.4(7.6, 11.2) | 21.8(18.6, 25.1) | 12.5(8.7, 16.2) |

**Note:**

1. World Bank region group: “EAP” represents “East Asia and Pacific”; “ECA” represents “Europe and Central Asia”; “LAC” represents “Latin America and the Caribbean”; “MENA” represents “Middle East and North Africa”; “SA” represents “South Asia”; “SSA” represents “Sub-Saharan Africa”.

2. Income group: “L” represents “Low-income country”; “LM” represents “Lower-middle income country”; “UM” represents “Upper-middle income country”.

*. The disparity in prevalence between young children in urban and rural areas is not statistically significant at the 0.05 level.

**Table S24.** National prevalence of young children living in households with preparedness and associated disparities by household wealth quintile (upper-bound)

| **Country** | **Year** | **WB region^1^** | **Income group^2^** | **Wealth quintile** | | | | | **Difference (richest-poorest)** |
| --- | --- | --- | --- | --- | --- | --- | --- | --- | --- |
|  |  |  |  | **Poorest (%)** | **2 (%)** | **3 (%)** | **4 (%)** | **Richest (%)** |  |
| Algeria | 2019 | MENA | UM | 26.0(22.9, 29.1) | 45.1(42.2, 48.1) | 63.5(60.4, 66.7) | 76.0(73.3, 78.8) | 83.3(80.2, 86.4) | 57.3(53.0, 61.6) |
| Angola | 2016 | SSA | UM | 0.0(0.0, 0.0) | 0.6(0.2, 1.0) | 4.9(3.5, 6.3) | 13.3(9.9, 16.7) | 44.9(38.2, 51.5) | 44.9(38.2, 51.5) |
| Armenia | 2016 | ECA | LM | 31.4(23.7, 39.2) | 58.9(52.0, 65.8) | 79.4(73.0, 85.8) | 87.3(82.4, 92.2) | 94.9(91.7, 98.1) | 63.4(55.0, 71.9) |
| Bangladesh | 2019 | SA | LM | 3.2(2.5, 3.8) | 14.4(13.2, 15.6) | 29.3(27.6, 31.1) | 43.2(41.2, 45.2) | 67.3(65.0, 69.6) | 64.1(61.8, 66.5) |
| Benin | 2018 | SSA | L | 0.0(0.0, 0.0) | 0.0(0.0, 0.1) | 0.0(0.0, 0.0) | 0.5(0.1, 0.8) | 8.8(6.9, 10.7) | 8.8(6.9, 10.7) |
| Burundi | 2017 | SSA | L | 0.0(0.0, 0.0) | 0.1(0.0, 0.2) | 0.0(0.0, 0.1) | 1.0(0.5, 1.6) | 7.1(5.2, 9.0) | 7.1(5.2, 9.0) |
| CAR | 2018 | SSA | L | 0.0(0.0, 0.0) | 0.0(0.0, 0.0) | 0.0(0.0, 0.0) | 0.8(-0.1, 1.7) | 6.0(4.3, 7.6) | 6.0(4.3, 7.6) |
| Cameroon | 2018 | SSA | LM | 0.0(0.0, 0.0) | 1.1(0.4, 1.8) | 4.4(2.9, 5.9) | 12.0(9.6, 14.3) | 45.4(41.0, 49.7) | 45.4(41.0, 49.7) |
| Chad | 2019 | SSA | L | 0.0(0.0, 0.0) | 0.0(0.0, 0.1) | 0.0(0.0, 0.1) | 0.6(0.2, 0.9) | 11.6(9.4, 13.7) | 11.6(9.4, 13.7) |
| Congo DR | 2018 | SSA | L | 0.0(0.0, 0.0) | 0.0(0.0, 0.0) | 0.1(0.0, 0.2) | 0.7(0.0, 1.4) | 10.3(6.9, 13.6) | 10.3(6.9, 13.6) |
| Cote d’Ivoire | 2016 | SSA | LM | 0.1(0.0, 0.2) | 0.3(0.1, 0.6) | 1.4(0.6, 2.1) | 5.7(3.7, 7.7) | 34.6(28.1, 41.0) | 34.5(28.0, 41.0) |
| Dominican Republic | 2019 | LAC | UM | 4.7(3.5, 5.9) | 23.6(20.4, 26.9) | 43.8(39.8, 47.8) | 64.0(59.2, 68.8) | 79.3(74.9, 83.8) | 74.6(70.0, 79.2) |
| Ethiopia | 2016 | SSA | L | 0.0(0.0, 0.0) | 0.0(0.0, 0.0) | 0.0(0.0, 0.0) | 0.0(0.0, 0.0) | 4.8(2.5, 7.0) | 4.8(2.5, 7.0) |
| Gambia | 2020 | SSA | L | 1.0(0.3, 1.6) | 0.8(0.2, 1.4) | 2.8(0.4, 5.2) | 4.5(2.4, 6.5) | 14.7(10.7, 18.8) | 13.8(9.7, 17.9) |
| Ghana | 2017 | SSA | LM | 1.5(0.0, 3.0) | 0.6(0.2, 1.1) | 4.4(1.8, 7.1) | 6.0(2.6, 9.4) | 32.4(27.8, 36.9) | 30.8(26.1, 35.6) |
| Guinea | 2018 | SSA | L | 0.3(-0.1, 0.6) | 0.3(-0.1, 0.7) | 1.2(0.4, 2.0) | 2.6(1.3, 3.8) | 15.4(12.0, 18.7) | 15.1(11.7, 18.5) |
| Guinea-Bissau | 2019 | SSA | L | 0.0(0.0, 0.0) | 0.1(-0.1, 0.3) | 0.7(0.0, 1.5) | 2.2(1.0, 3.4) | 9.0(5.8, 12.2) | 9.0(5.8, 12.2) |
| Guyana | 2019 | LAC | UM | 21.8(16.1, 27.4) | 52.0(42.8, 61.2) | 68.4(58.1, 78.7) | 70.4(60.8, 80.0) | 85.4(79.7, 91.0) | 63.6(55.8, 71.3) |
| Haiti | 2017 | LAC | L | 0.0(0.0, 0.0) | 1.6(0.5, 2.8) | 2.0(1.0, 3.1) | 5.2(3.1, 7.3) | 23.3(19.2, 27.5) | 23.3(19.2, 27.5) |
| India | 2016 | SA | LM | 0.2(0.1, 0.2) | 3.2(3.0, 3.5) | 15.3(14.8, 15.9) | 41.8(40.7, 42.9) | 76.3(75.2, 77.3) | 76.1(75.1, 77.2) |
| Indonesia | 2017 | EAP | LM | 13.8(12.2, 15.4) | 45.2(42.5, 47.9) | 65.0(62.5, 67.5) | 78.6(76.6, 80.7) | 84.5(82.4, 86.5) | 70.6(68.0, 73.2) |
| Iraq | 2018 | MENA | UM | 37.3(33.8, 40.9) | 62.7(58.7, 66.8) | 67.9(63.0, 72.9) | 76.7(73.6, 79.8) | 80.0(76.7, 83.3) | 42.7(37.8, 47.5) |
| Kiribati | 2018 | EAP | LM | 0.8(-0.2, 1.7) | 5.3(2.8, 7.8) | 5.7(2.9, 8.4) | 11.4(6.5, 16.2) | 31.5(23.8, 39.2) | 30.8(22.9, 38.6) |
| Kyrgyzstan* | 2018 | ECA | LM | 65.5(60.7, 70.2) | 73.1(68.4, 77.8) | 71.9(66.1, 77.7) | 70.9(66.1, 75.8) | 65.6(59.6, 71.6) | 0.1(-7.5, 7.8) |
| Lao | 2017 | EAP | LM | 0.9(0.4, 1.4) | 9.7(8.2, 11.2) | 27.5(24.8, 30.2) | 51.8(48.8, 54.8) | 78.7(75.9, 81.4) | 77.7(75.0, 80.5) |
| Lesotho | 2018 | SSA | LM | 0.0(0.0, 0.0) | 0.2(0.0, 0.5) | 1.9(0.3, 3.5) | 4.6(2.5, 6.8) | 21.2(15.3, 27.1) | 21.2(15.3, 27.1) |
| Liberia | 2019 | SSA | L | 0.0(0.0, 0.0) | 0.0(0.0, 0.0) | 0.1(-0.1, 0.3) | 0.5(-0.2, 1.3) | 5.6(2.5, 8.7) | 5.6(2.5, 8.7) |
| Madagascar | 2018 | SSA | L | 0.0(0.0, 0.0) | 0.0(0.0, 0.1) | 0.3(0.1, 0.5) | 2.9(1.9, 3.9) | 12.7(10.6, 14.7) | 12.7(10.6, 14.8) |
| Malawi | 2020 | SSA | L | 0.1(0.0, 0.1) | 1.1(0.6, 1.5) | 3.8(2.9, 4.6) | 7.0(5.4, 8.6) | 23.7(19.7, 27.7) | 23.6(19.6, 27.6) |
| Maldives | 2017 | SA | UM | 74.2(69.9, 78.6) | 82.3(78.2, 86.3) | 85.0(80.9, 89.0) | 80.2(71.7, 88.7) | 89.0(79.6, 98.5) | 14.8(4.4, 25.3) |
| Mali | 2018 | SSA | L | 0.2(-0.1, 0.5) | 1.5(0.6, 2.3) | 2.3(1.0, 3.5) | 5.1(3.5, 6.7) | 12.8(10.1, 15.5) | 12.6(9.9, 15.3) |
| Mongolia | 2018 | EAP | LM | 2.6(1.3, 3.8) | 13.0(10.1, 15.8) | 22.3(16.9, 27.7) | 62.2(56.1, 68.3) | 86.6(82.5, 90.7) | 84.0(79.7, 88.3) |
| Myanmar | 2016 | EAP | LM | 1.5(0.5, 2.5) | 5.5(3.7, 7.3) | 13.2(10.2, 16.2) | 28.7(24.4, 33.0) | 53.6(47.9, 59.2) | 52.1(46.3, 57.8) |
| Nepal | 2019 | SA | L | 11.9(9.8, 13.9) | 30.8(26.6, 35.0) | 39.6(35.2, 44.0) | 52.9(48.2, 57.5) | 56.8(50.6, 63.0) | 44.9(38.4, 51.5) |
| Nigeria | 2018 | SSA | LM | 0.1(0.0, 0.3) | 0.6(0.3, 0.9) | 2.3(1.7, 3.0) | 8.0(6.7, 9.2) | 28.6(25.6, 31.7) | 28.5(25.4, 31.6) |
| PNG | 2016-18 | SA | LM | 0.0(0.0, 0.0) | 0.1(-0.1, 0.3) | 0.2(0.0, 0.4) | 2.5(1.4, 3.5) | 29.3(18.2, 40.4) | 29.3(18.2, 40.4) |
| Pakistan | 2018 | EAP | LM | 0.6(0.0, 1.2) | 2.7(1.5, 4.0) | 15.9(13.0, 18.8) | 37.9(33.5, 42.3) | 64.1(58.4, 69.7) | 63.4(57.8, 69.1) |
| Palestine | 2019 | MENA | LM | 40.4(35.8, 45.1) | 57.7(53.1, 62.3) | 61.9(57.7, 66.2) | 75.2(71.9, 78.6) | 81.1(78.1, 84.2) | 40.7(35.1, 46.2) |
| Paraguay | 2016 | LAC | UM | 15.7(12.6, 18.8) | 56.3(50.9, 61.6) | 83.1(79.0, 87.2) | 88.3(84.1, 92.5) | 92.7(89.6, 95.8) | 77.0(72.6, 81.3) |
| Philippines | 2017 | EAP | LM | 10.0(8.3, 11.7) | 26.0(23.0, 29.1) | 45.5(41.3, 49.7) | 70.4(66.7, 74.1) | 78.3(72.2, 84.5) | 68.3(61.9, 74.8) |
| Rwanda | 2020 | SSA | L | 0.7(0.2, 1.2) | 4.3(3.1, 5.5) | 9.9(8.0, 11.7) | 14.1(11.6, 16.5) | 27.8(23.8, 31.9) | 27.2(23.1, 31.3) |
| Samoa | 2019 | EAP | L | 6.5(2.2, 10.9) | 16.3(12.0, 20.5) | 36.4(29.4, 43.4) | 49.8(43.2, 56.4) | 61.3(53.1, 69.5) | 54.8(45.6, 64.0) |
| STP | 2019 | SSA | LM | 1.1(0.1, 2.0) | 5.1(2.2, 8.0) | 10.8(7.2, 14.4) | 32.0(24.5, 39.5) | 66.5(57.7, 75.3) | 65.4(56.6, 74.3) |
| Senegal | 2019 | SSA | L | 2.6(1.2, 3.9) | 3.6(2.2, 5.1) | 2.5(1.5, 3.5) | 4.4(2.3, 6.4) | 27.4(19.5, 35.3) | 24.9(16.8, 32.9) |
| Sierra Leone | 2019 | SSA | L | 0.0(0.0, 0.0) | 0.0(0.0, 0.1) | 0.5(0.1, 0.9) | 1.2(0.4, 2.1) | 7.6(5.2, 10.0) | 7.6(5.2, 10.0) |
| South Africa | 2016 | SSA | UM | 2.6(1.4, 3.8) | 6.5(4.3, 8.7) | 14.8(11.0, 18.6) | 43.3(36.3, 50.3) | 79.0(73.0, 85.1) | 76.5(70.3, 82.7) |
| Suriname | 2018 | LAC | UM | 18.6(14.9, 22.3) | 53.5(47.4, 59.6) | 60.7(53.9, 67.5) | 74.2(67.6, 80.8) | 78.2(70.5, 85.8) | 59.5(50.9, 68.2) |
| Tajikistan | 2017 | ECA | LM | 32.9(26.8, 39.0) | 46.4(40.0, 52.7) | 59.3(53.9, 64.8) | 65.4(60.1, 70.6) | 72.7(68.2, 77.3) | 39.8(32.3, 47.4) |
| Timor-Leste | 2016 | EAP | LM | 0.0(0.0, 0.1) | 0.7(0.2, 1.3) | 4.4(2.9, 5.8) | 12.0(7.9, 16.1) | 38.7(34.1, 43.3) | 38.7(34.1, 43.3) |
| Togo | 2017 | SSA | L | 0.0(0.0, 0.0) | 0.2(-0.2, 0.7) | 0.4(-0.2, 0.9) | 2.7(0.9, 4.5) | 22.9(18.0, 27.7) | 22.9(18.0, 27.7) |
| Tonga | 2019 | EAP | LM | 21.2(14.5, 27.8) | 42.8(31.2, 54.4) | 58.2(46.7, 69.7) | 64.3(53.4, 75.2) | 79.4(71.9, 86.9) | 58.2(48.6, 67.9) |
| Tunisia | 2018 | MENA | LM | 63.6(58.7, 68.5) | 76.4(72.0, 80.7) | 82.6(78.6, 86.7) | 93.2(90.6, 95.8) | 94.1(91.4, 96.8) | 30.5(24.9, 36.1) |
| Tuvalu | 2019 | EAP | UM | 16.3(7.3, 25.3) | 36.5(25.4, 47.6) | 45.6(33.5, 57.7) | 60.5(45.1, 75.9) | 69.8(56.2, 83.3) | 53.5(36.1, 70.9) |
| Uganda | 2016 | SSA | L | 0.0(0.0, 0.1) | 0.1(0.0, 0.3) | 1.0(0.4, 1.6) | 3.8(2.7, 4.9) | 15.9(13.3, 18.4) | 15.8(13.2, 18.4) |
| Zambia | 2018 | SSA | LM | 0.0(0.0, 0.1) | 0.4(0.1, 0.7) | 0.4(0.0, 0.7) | 1.6(0.5, 2.7) | 11.2(8.1, 14.3) | 11.2(8.1, 14.3) |
| Zimbabwe | 2019 | SSA | L | 0.9(0.3, 1.5) | 5.8(3.9, 7.7) | 15.8(12.6, 19.0) | 15.2(11.8, 18.6) | 34.6(29.8, 39.4) | 33.7(28.9, 38.5) |

**Note:**

1. World Bank region group: “EAP” represents “East Asia and Pacific”; “ECA” represents “Europe and Central Asia”; “LAC” represents “Latin America and the Caribbean”; “MENA” represents “Middle East and North Africa”; “SA” represents “South Asia”; “SSA” represents “Sub-Saharan Africa”.

2. Income group: “L” represents “Low-income country”; “LM” represents “Lower-middle income country”; “UM” represents “Upper-middle income country”.

*. The disparity in prevalence between young children living in the wealthiest and poorest quintiles is not statistically significant at the 0.05 level.

**Table S25.** National prevalence of young children living in households with adequate quarantine condition and associated disparities by place of residence (lower-bound)

| Country | Year | WB region^1^ | Income group^2^ | National (%) | Rural (%) | Urban (%) | Difference  (urban-rural) |
| --- | --- | --- | --- | --- | --- | --- | --- |
| Algeria | 2019 | MENA | UM | 30.2(28.8, 31.7) | 25.1(22.9, 27.2) | 34.0(32.1, 35.9) | 8.9(6.1, 11.8) |
| Angola | 2016 | SSA | UM | 21.2(19.8, 22.6) | 19.5(17.7, 21.3) | 22.4(20.3, 24.5) | 2.9(0.2, 5.7) |
| Armenia* | 2016 | ECA | LM | 39.7(36.5, 42.9) | 41.0(36.4, 45.7) | 38.7(34.3, 43.1) | -2.3(-8.7, 4.0) |
| Bangladesh* | 2019 | SA | LM | 33.7(32.9, 34.6) | 33.6(32.7, 34.6) | 34.0(32.0, 36.0) | 0.3(-1.9, 2.5) |
| Benin* | 2018 | SSA | L | 25.6(24.3, 27.0) | 24.9(23.1, 26.7) | 26.8(24.7, 28.8) | 1.8(-0.9, 4.6) |
| Burundi* | 2017 | SSA | L | 26.8(25.6, 28.0) | 26.6(25.4, 27.9) | 28.8(24.8, 32.8) | 2.2(-2.0, 6.4) |
| CAR | 2018 | SSA | L | 23.0(21.1, 24.8) | 21.7(19.3, 24.0) | 26.0(23.0, 28.9) | 4.3(0.5, 8.1) |
| Cameroon* | 2018 | SSA | LM | 29.0(27.3, 30.7) | 28.8(26.2, 31.4) | 29.2(27.3, 31.2) | 0.4(-2.8, 3.7) |
| Chad | 2019 | SSA | L | 26.2(24.9, 27.6) | 25.6(24.1, 27.1) | 29.5(26.4, 32.7) | 3.9(0.4, 7.4) |
| Congo DR | 2018 | SSA | L | 20.7(19.3, 22.2) | 23.3(21.5, 25.1) | 16.7(14.3, 19.0) | -6.7(-9.6, -3.7) |
| Cote d’Ivoire | 2016 | SSA | LM | 20.6(19.1, 22.1) | 22.0(19.9, 24.0) | 18.5(16.3, 20.7) | -3.5(-6.5, -0.4) |
| Dominican Republic | 2019 | LAC | UM | 43.5(41.7, 45.4) | 39.9(36.7, 43.1) | 44.8(42.5, 47.1) | 4.9(1.0, 8.8) |
| Ethiopia | 2016 | SSA | L | 7.3(6.2, 8.3) | 5.8(4.8, 6.8) | 19.1(14.7, 23.6) | 13.4(8.8, 17.9) |
| Gambia* | 2020 | SSA | L | 32.4(29.9, 34.9) | 33.7(29.6, 37.8) | 31.8(28.7, 34.9) | -1.9(-7.1, 3.3) |
| Ghana* | 2017 | SSA | LM | 17.3(15.6, 18.9) | 16.5(14.2, 18.8) | 18.3(15.9, 20.7) | 1.8(-1.6, 5.1) |
| Guinea | 2018 | SSA | L | 30.3(28.3, 32.3) | 32.1(29.7, 34.6) | 25.6(22.4, 28.8) | -6.5(-10.7, -2.4) |
| Guinea-Bissau | 2019 | SSA | L | 45.0(42.8, 47.2) | 50.2(47.3, 53.2) | 30.1(27.3, 32.8) | -20.2(-24.2, -16.1) |
| Guyana* | 2019 | LAC | UM | 40.3(36.8, 43.9) | 39.3(35.0, 43.7) | 43.2(37.7, 48.8) | 3.9(-3.2, 10.9) |
| Haiti* | 2017 | LAC | L | 22.0(20.2, 23.8) | 22.1(19.8, 24.3) | 21.9(19.0, 24.7) | -0.2(-3.8, 3.4) |
| India | 2016 | SA | LM | 17.9(17.6, 18.2) | 16.9(16.6, 17.2) | 20.5(19.8, 21.3) | 3.7(2.9, 4.5) |
| Indonesia | 2017 | EAP | LM | 51.9(50.7, 53.1) | 53.9(52.2, 55.7) | 49.7(48.0, 51.4) | -4.2(-6.7, -1.8) |
| Iraq | 2018 | MENA | UM | 11.7(10.5, 12.9) | 9.3(7.9, 10.7) | 12.8(11.2, 14.4) | 3.5(1.4, 5.7) |
| Kiribati | 2018 | EAP | LM | 14.6(12.7, 16.4) | 16.7(14.1, 19.4) | 12.6(9.9, 15.3) | -4.2(-8.0, -0.4) |
| Kyrgyzstan | 2018 | ECA | LM | 31.1(28.8, 33.4) | 28.9(26.2, 31.6) | 35.9(32.0, 39.8) | 7.0(2.2, 11.7) |
| Lao | 2017 | EAP | LM | 18.8(17.7, 19.9) | 13.9(12.7, 15.1) | 31.9(29.4, 34.4) | 18.0(15.3, 20.8) |
| Lesotho | 2018 | SSA | LM | 19.2(17.3, 21.2) | 16.1(14.0, 18.2) | 25.4(21.6, 29.2) | 9.3(4.9, 13.7) |
| Liberia | 2019 | SSA | L | 37.6(35.2, 40.0) | 41.0(38.4, 43.7) | 34.6(30.7, 38.6) | -6.4(-11.2, -1.6) |
| Madagascar | 2018 | SSA | L | 8.3(7.6, 9.0) | 7.3(6.5, 8.1) | 12.5(10.6, 14.3) | 5.2(3.2, 7.2) |
| Malawi | 2020 | SSA | L | 28.9(27.6, 30.3) | 27.9(26.5, 29.3) | 36.3(32.6, 40.0) | 8.4(4.5, 12.4) |
| Maldives | 2017 | SA | UM | 36.8(34.2, 39.4) | 43.6(41.3, 45.9) | 24.4(18.1, 30.6) | -19.2(-25.9, -12.5) |
| Mali | 2018 | SSA | L | 34.7(32.8, 36.6) | 35.6(33.4, 37.8) | 31.2(27.8, 34.6) | -4.4(-8.5, -0.3) |
| Mongolia* | 2018 | EAP | LM | 21.6(19.1, 24.0) | 19.4(15.4, 23.3) | 22.1(19.2, 25.0) | 2.8(-2.2, 7.7) |
| Myanmar | 2016 | EAP | LM | 15.4(13.8, 17.1) | 14.0(12.2, 15.9) | 20.3(16.6, 24.0) | 6.2(2.1, 10.4) |
| Nepal | 2019 | SA | L | 35.5(33.4, 37.6) | 32.7(29.9, 35.4) | 37.1(34.2, 39.9) | 4.4(0.4, 8.4) |
| Nigeria | 2018 | SSA | LM | 27.6(26.6, 28.7) | 26.7(25.4, 28.0) | 29.1(27.5, 30.7) | 2.4(0.3, 4.4) |
| PNG* | 2016-18 | SA | LM | 33.3(30.6, 35.9) | 33.8(30.9, 36.7) | 29.2(25.2, 33.3) | -4.6(-9.6, 0.5) |
| Pakistan* | 2018 | EAP | LM | 8.2(7.2, 9.3) | 7.8(6.4, 9.1) | 9.3(7.6, 10.9) | 1.5(-0.6, 3.7) |
| Palestine* | 2019 | MENA | LM | 20.4(18.8, 22.0) | 21.2(18.8, 23.5) | 20.2(18.1, 22.2) | -1.0(-4.1, 2.1) |
| Paraguay | 2016 | LAC | UM | 37.6(35.1, 40.1) | 30.5(27.2, 33.8) | 42.4(38.7, 46.0) | 11.9(7.0, 16.8) |
| Philippines* | 2017 | EAP | LM | 15.8(14.6, 17.1) | 15.8(14.4, 17.3) | 15.8(13.6, 18.0) | -0.1(-2.6, 2.5) |
| Rwanda | 2020 | SSA | L | 45.9(44.2, 47.6) | 44.6(42.8, 46.5) | 51.8(47.6, 56.0) | 7.1(2.6, 11.7) |
| Samoa | 2019 | EAP | L | 14.4(12.4, 16.3) | 12.4(10.4, 14.4) | 24.6(19.5, 29.7) | 12.2(6.7, 17.8) |
| STP* | 2019 | SSA | LM | 32.9(29.8, 35.9) | 31.5(26.5, 36.5) | 33.5(29.8, 37.3) | 2.0(-4.3, 8.4) |
| Senegal* | 2019 | SSA | L | 23.8(21.7, 26.0) | 22.7(20.3, 25.2) | 25.7(21.6, 29.9) | 3.0(-1.9, 7.9) |
| Sierra Leone | 2019 | SSA | L | 36.4(34.7, 38.1) | 39.4(37.2, 41.7) | 30.8(28.3, 33.3) | -8.6(-12.0, -5.2) |
| South Africa* | 2016 | SSA | UM | 48.1(44.9, 51.3) | 48.2(44.5, 51.8) | 48.0(43.5, 52.6) | -0.2(-6.0, 5.7) |
| Suriname | 2018 | LAC | UM | 36.7(34.1, 39.2) | 24.0(20.6, 27.4) | 43.2(39.8, 46.6) | 19.2(14.4, 24.0) |
| Tajikistan | 2017 | ECA | LM | 17.0(15.4, 18.6) | 15.0(13.2, 16.8) | 24.6(21.5, 27.7) | 9.6(6.0, 13.2) |
| Timor-Leste* | 2016 | EAP | LM | 40.7(38.7, 42.7) | 40.0(37.7, 42.3) | 42.3(38.4, 46.3) | 2.3(-2.2, 6.9) |
| Togo* | 2017 | SSA | L | 25.2(23.3, 27.2) | 26.3(23.8, 28.8) | 23.6(20.3, 26.8) | -2.7(-6.8, 1.4) |
| Tonga* | 2019 | EAP | LM | 39.6(35.1, 44.1) | 40.1(34.6, 45.5) | 37.9(31.4, 44.3) | -2.2(-10.7, 6.3) |
| Tunisia | 2018 | MENA | LM | 47.4(45.0, 49.8) | 39.6(35.7, 43.4) | 51.8(48.9, 54.7) | 12.2(7.4, 17.0) |
| Tuvalu* | 2019 | EAP | UM | 18.1(13.3, 22.8) | 21.9(14.9, 29.0) | 16.1(10.0, 22.2) | -5.9(-15.4, 3.6) |
| Uganda | 2016 | SSA | L | 22.8(21.6, 23.9) | 21.1(19.8, 22.3) | 29.1(26.5, 31.7) | 8.0(5.1, 10.9) |
| Zambia | 2018 | SSA | LM | 19.9(18.6, 21.2) | 18.8(17.3, 20.3) | 21.8(19.3, 24.4) | 3.0(0.1, 6.0) |
| Zimbabwe | 2019 | SSA | L | 25.8(24.1, 27.4) | 23.7(21.9, 25.5) | 30.8(27.4, 34.2) | 7.1(3.3, 11.0) |

**Note:**

1. World Bank region group: “EAP” represents “East Asia and Pacific”; “ECA” represents “Europe and Central Asia”; “LAC” represents “Latin America and the Caribbean”; “MENA” represents “Middle East and North Africa”; “SA” represents “South Asia”; “SSA” represents “Sub-Saharan Africa”.

2. Income group: “L” represents “Low-income country”; “LM” represents “Lower-middle income country”; “UM” represents “Upper-middle income country”.

*. The disparity in prevalence between young children in urban and rural areas is not statistically significant at the 0.05 level.

**Table S26.** National prevalence of young children living in households with adequate quarantine condition and associated disparities by household wealth quintile (lower-bound)

| **Country** | **Year** | **WB region^1^** | **Income group^2^** | **Wealth quintile** | | | | | **Difference (richest-poorest)** | |  |
| --- | --- | --- | --- | --- | --- | --- | --- | --- | --- | --- | --- |
|  |  |  |  | **Poorest (%)** | **2 (%)** | **3 (%)** | **4 (%)** | **Richest (%)** |  |  |  |
| Algeria | 2019 | MENA | UM | 18.6(16.6, 20.6) | 24.3(21.9, 26.7) | 31.5(28.8, 34.1) | 38.3(35.0, 41.6) | 46.4(43.2, 49.6) | | 27.7(24.1, 31.4) | |
| Angola | 2016 | SSA | UM | 17.1(14.8, 19.5) | 21.0(18.8, 23.1) | 17.8(15.0, 20.6) | 20.7(17.6, 23.9) | 34.0(28.9, 39.2) | | 16.9(11.2, 22.6) | |
| Armenia* | 2016 | ECA | LM | 33.1(26.4, 39.7) | 46.6(40.2, 53.1) | 40.9(33.9, 47.8) | 35.1(28.4, 41.7) | 41.7(34.2, 49.2) | | 8.6(-1.3, 18.6) | |
| Bangladesh | 2019 | SA | LM | 15.9(14.7, 17.2) | 22.1(20.7, 23.6) | 36.1(34.3, 38.0) | 44.1(42.1, 46.2) | 51.4(49.2, 53.6) | | 35.4(32.9, 38.0) | |
| Benin | 2018 | SSA | L | 21.1(18.3, 24.0) | 25.1(22.4, 27.7) | 26.4(23.4, 29.5) | 27.1(24.6, 29.7) | 29.2(26.4, 31.9) | | 8.0(4.0, 12.0) | |
| Burundi | 2017 | SSA | L | 22.0(19.9, 24.1) | 25.1(22.6, 27.6) | 26.9(24.5, 29.4) | 28.1(25.6, 30.6) | 33.8(30.5, 37.1) | | 11.8(8.0, 15.6) | |
| CAR | 2018 | SSA | L | 19.1(15.3, 22.8) | 18.7(15.5, 21.9) | 23.5(20.1, 26.9) | 23.9(20.4, 27.4) | 33.5(29.6, 37.4) | | 14.4(9.0, 19.8) | |
| Cameroon | 2018 | SSA | LM | 17.5(14.3, 20.8) | 30.7(27.0, 34.3) | 31.6(28.2, 35.0) | 28.0(24.5, 31.5) | 41.2(37.5, 44.9) | | 23.6(18.8, 28.5) | |
| Chad | 2019 | SSA | L | 12.5(10.9, 14.1) | 19.8(17.6, 22.0) | 28.3(25.8, 30.8) | 36.9(33.7, 40.0) | 37.2(33.9, 40.5) | | 24.7(21.1, 28.4) | |
| Congo DR | 2018 | SSA | L | 21.5(18.8, 24.1) | 23.0(20.5, 25.5) | 23.3(20.2, 26.4) | 20.2(16.4, 24.0) | 13.9(11.0, 16.7) | | -7.6(-11.5, -3.7) | |
| Cote d’Ivoire* | 2016 | SSA | LM | 20.5(17.6, 23.4) | 21.9(18.7, 25.0) | 22.8(19.9, 25.8) | 17.0(13.7, 20.3) | 20.5(16.2, 24.7) | | 0.0(-5.2, 5.2) | |
| Dominican Republic | 2019 | LAC | UM | 26.3(23.4, 29.1) | 32.3(29.0, 35.6) | 44.2(40.0, 48.3) | 54.9(50.2, 59.6) | 70.8(66.0, 75.6) | | 44.5(38.8, 50.2) | |
| Ethiopia | 2016 | SSA | L | 2.5(1.6, 3.4) | 3.1(1.9, 4.4) | 6.6(4.6, 8.6) | 11.4(8.8, 14.0) | 17.4(14.2, 20.6) | | 14.9(11.7, 18.1) | |
| Gambia | 2020 | SSA | L | 26.2(22.6, 29.8) | 35.3(30.5, 40.1) | 29.6(24.8, 34.3) | 31.9(25.8, 38.0) | 41.3(34.5, 48.1) | | 15.1(7.4, 22.8) | |
| Ghana | 2017 | SSA | LM | 17.9(15.0, 20.7) | 13.7(10.2, 17.3) | 13.4(10.3, 16.5) | 11.7(9.1, 14.2) | 30.4(26.0, 34.9) | | 12.6(7.2, 17.9) | |
| Guinea* | 2018 | SSA | L | 24.8(21.4, 28.2) | 33.0(28.9, 37.1) | 36.1(31.8, 40.5) | 29.6(25.8, 33.4) | 28.2(24.0, 32.4) | | 3.4(-2.0, 8.8) | |
| Guinea-Bissau | 2019 | SSA | L | 48.3(43.9, 52.7) | 51.0(46.1, 55.9) | 51.5(46.7, 56.2) | 38.5(33.9, 43.0) | 27.4(23.2, 31.6) | | -21.0(-27.0, -14.9) | |
| Guyana | 2019 | LAC | UM | 18.2(14.6, 21.8) | 36.1(29.5, 42.6) | 45.2(36.8, 53.6) | 55.1(46.8, 63.4) | 63.2(55.5, 71.0) | | 45.0(36.6, 53.5) | |
| Haiti | 2017 | LAC | L | 16.7(13.9, 19.5) | 20.0(16.8, 23.2) | 19.6(16.2, 23.1) | 24.3(20.0, 28.6) | 34.6(29.6, 39.6) | | 17.9(12.2, 23.6) | |
| India | 2016 | SA | LM | 9.6(9.2, 10.0) | 13.9(13.4, 14.4) | 17.1(16.5, 17.7) | 21.8(21.0, 22.5) | 34.4(33.3, 35.5) | | 24.8(23.7, 25.9) | |
| Indonesia | 2017 | EAP | LM | 33.3(31.0, 35.6) | 47.7(45.1, 50.3) | 53.5(51.1, 55.8) | 57.3(54.9, 59.8) | 69.0(66.5, 71.4) | | 35.7(32.3, 39.0) | |
| Iraq | 2018 | MENA | UM | 7.2(5.5, 8.8) | 7.2(5.7, 8.8) | 10.3(8.3, 12.2) | 18.6(15.1, 22.1) | 17.7(15.0, 20.4) | | 10.5(7.4, 13.7) | |
| Kiribati* | 2018 | EAP | LM | 12.6(9.0, 16.1) | 18.1(13.8, 22.3) | 14.5(10.5, 18.6) | 11.9(7.9, 15.9) | 16.0(11.1, 20.9) | | 3.4(-2.7, 9.5) | |
| Kyrgyzstan | 2018 | ECA | LM | 26.5(21.9, 31.0) | 23.9(19.1, 28.8) | 35.8(30.3, 41.2) | 34.1(29.5, 38.6) | 39.2(33.2, 45.1) | | 12.7(5.1, 20.3) | |
| Lao | 2017 | EAP | LM | 4.4(3.5, 5.3) | 10.0(8.5, 11.5) | 15.7(13.7, 17.8) | 27.0(24.5, 29.5) | 47.1(43.7, 50.5) | | 42.7(39.2, 46.2) | |
| Lesotho | 2018 | SSA | LM | 9.7(7.3, 12.0) | 12.1(9.3, 14.9) | 15.3(11.7, 18.9) | 23.7(18.4, 28.9) | 40.8(34.6, 47.0) | | 31.1(24.4, 37.8) | |
| Liberia | 2019 | SSA | L | 38.9(34.4, 43.5) | 42.2(38.1, 46.3) | 47.4(42.6, 52.2) | 28.8(22.0, 35.6) | 28.4(21.7, 35.1) | | -10.6(-18.7, -2.4) | |
| Madagascar | 2018 | SSA | L | 4.8(3.9, 5.8) | 5.1(4.1, 6.0) | 5.8(4.5, 7.0) | 10.9(9.2, 12.7) | 19.9(17.1, 22.6) | | 15.0(12.1, 17.9) | |
| Malawi | 2020 | SSA | L | 18.0(15.9, 20.1) | 24.4(22.0, 26.9) | 33.4(30.6, 36.2) | 34.3(31.7, 37.0) | 42.0(38.2, 45.8) | | 24.0(19.7, 28.4) | |
| Maldives | 2017 | SA | UM | 40.6(36.9, 44.3) | 41.1(36.5, 45.7) | 41.0(36.2, 45.8) | 27.8(20.2, 35.4) | 31.0(22.3, 39.6) | | -9.6(-19.0, -0.2) | |
| Mali | 2018 | SSA | L | 28.4(24.3, 32.5) | 34.4(31.0, 37.9) | 40.7(37.3, 44.0) | 30.1(26.8, 33.5) | 40.3(36.3, 44.3) | | 12.0(6.2, 17.7) | |
| Mongolia | 2018 | EAP | LM | 8.7(2.7, 14.6) | 7.6(5.0, 10.2) | 12.9(9.3, 16.6) | 26.3(21.5, 31.2) | 29.8(24.6, 35.0) | | 21.2(13.3, 29.0) | |
| Myanmar | 2016 | EAP | LM | 9.7(7.5, 11.8) | 11.0(8.4, 13.7) | 13.3(10.1, 16.4) | 19.4(15.8, 22.9) | 32.2(26.1, 38.3) | | 22.6(16.0, 29.1) | |
| Nepal | 2019 | SA | L | 25.5(22.6, 28.4) | 32.4(28.4, 36.5) | 35.0(30.8, 39.2) | 38.8(35.1, 42.6) | 50.2(44.1, 56.3) | | 24.7(18.0, 31.5) | |
| Nigeria | 2018 | SSA | LM | 22.5(20.3, 24.7) | 25.0(23.0, 26.9) | 28.7(26.8, 30.6) | 28.8(26.6, 31.0) | 35.7(33.2, 38.2) | | 13.1(9.8, 16.5) | |
| PNG | 2016-18 | SA | LM | 26.7(23.0, 30.5) | 29.4(24.9, 33.8) | 32.9(28.5, 37.2) | 36.7(32.9, 40.5) | 41.7(31.9, 51.4) | | 14.9(4.5, 25.4) | |
| Pakistan | 2018 | EAP | LM | 3.0(1.8, 4.2) | 5.7(3.6, 7.8) | 7.0(4.9, 9.0) | 12.6(9.6, 15.5) | 14.8(11.0, 18.6) | | 11.8(7.8, 15.8) | |
| Palestine | 2019 | MENA | LM | 5.6(3.8, 7.4) | 12.7(9.4, 16.0) | 22.8(19.3, 26.3) | 25.4(22.0, 28.7) | 39.6(35.8, 43.5) | | 34.0(29.8, 38.2) | |
| Paraguay | 2016 | LAC | UM | 13.9(10.6, 17.2) | 27.6(23.6, 31.6) | 41.1(36.1, 46.1) | 49.9(44.8, 55.0) | 72.8(67.4, 78.2) | | 58.9(52.4, 65.4) | |
| Philippines | 2017 | EAP | LM | 6.8(5.4, 8.3) | 10.7(9.0, 12.5) | 16.1(13.4, 18.7) | 19.4(15.5, 23.2) | 38.4(32.5, 44.3) | | 31.6(25.5, 37.6) | |
| Rwanda | 2020 | SSA | L | 30.3(27.7, 32.9) | 42.7(39.4, 46.0) | 46.4(42.9, 49.8) | 48.4(45.0, 51.9) | 66.1(62.3, 69.9) | | 35.8(31.2, 40.4) | |
| Samoa | 2019 | EAP | L | 1.8(0.7, 3.0) | 7.3(4.5, 10.0) | 13.1(9.5, 16.7) | 22.5(16.9, 28.1) | 35.7(28.8, 42.5) | | 33.8(26.8, 40.8) | |
| STP | 2019 | SSA | LM | 19.9(14.3, 25.4) | 20.0(15.2, 24.9) | 28.5(23.1, 33.9) | 44.1(37.9, 50.3) | 60.2(53.7, 66.7) | | 40.3(32.1, 48.5) | |
| Senegal | 2019 | SSA | L | 20.4(16.6, 24.2) | 22.1(17.1, 27.2) | 19.1(14.8, 23.4) | 25.3(20.2, 30.4) | 34.7(28.6, 40.8) | | 14.3(7.1, 21.5) | |
| Sierra Leone* | 2019 | SSA | L | 36.4(33.2, 39.6) | 42.7(39.4, 45.9) | 36.7(33.3, 40.0) | 31.4(27.9, 34.8) | 33.1(29.0, 37.1) | | -3.4(-8.6, 1.9) | |
| South Africa | 2016 | SSA | UM | 36.3(30.2, 42.4) | 46.7(40.7, 52.7) | 44.8(39.0, 50.6) | 47.9(41.5, 54.2) | 76.0(69.1, 82.8) | | 39.6(30.4, 48.8) | |
| Suriname | 2018 | LAC | UM | 16.5(12.9, 20.1) | 32.8(28.6, 36.9) | 41.5(35.5, 47.5) | 51.3(45.0, 57.7) | 66.7(60.0, 73.4) | | 50.2(42.6, 57.9) | |
| Tajikistan | 2017 | ECA | LM | 13.2(10.1, 16.4) | 14.0(10.9, 17.0) | 14.9(12.1, 17.6) | 18.0(14.6, 21.3) | 26.5(22.9, 30.1) | | 13.2(8.4, 18.1) | |
| Timor-Leste | 2016 | EAP | LM | 24.1(20.8, 27.4) | 36.9(32.9, 40.9) | 44.6(40.8, 48.5) | 48.1(43.9, 52.2) | 50.4(45.3, 55.6) | | 26.4(20.2, 32.5) | |
| Togo | 2017 | SSA | L | 22.7(19.0, 26.5) | 26.4(22.3, 30.5) | 23.3(19.3, 27.2) | 18.8(14.5, 23.1) | 36.4(31.1, 41.7) | | 13.7(7.1, 20.2) | |
| Tonga | 2019 | EAP | LM | 21.6(15.2, 28.1) | 32.1(22.3, 42.0) | 45.8(35.2, 56.4) | 48.4(38.5, 58.3) | 56.7(44.4, 68.9) | | 35.0(20.7, 49.3) | |
| Tunisia | 2018 | MENA | LM | 30.4(26.0, 34.9) | 41.1(36.0, 46.1) | 48.1(43.2, 53.1) | 53.4(48.4, 58.4) | 65.2(59.6, 70.8) | | 34.8(27.5, 42.0) | |
| Tuvalu* | 2019 | EAP | UM | 15.4(7.4, 23.4) | 9.1(1.9, 16.3) | 18.7(9.3, 28.1) | 17.0(6.5, 27.6) | 31.8(15.6, 48.1) | | 16.4(-2.0, 34.8) | |
| Uganda | 2016 | SSA | L | 10.4(8.9, 11.8) | 19.5(17.4, 21.7) | 25.1(22.5, 27.7) | 27.8(25.0, 30.7) | 33.9(31.0, 36.7) | | 23.5(20.3, 26.6) | |
| Zambia | 2018 | SSA | LM | 14.7(12.8, 16.6) | 18.3(15.8, 20.8) | 20.9(18.4, 23.3) | 20.6(17.8, 23.4) | 28.3(24.1, 32.4) | | 13.5(9.0, 18.1) | |
| Zimbabwe | 2019 | SSA | L | 14.1(11.7, 16.5) | 21.2(18.4, 23.9) | 31.0(27.8, 34.3) | 25.3(21.7, 28.9) | 42.7(37.7, 47.7) | | 28.6(23.0, 34.2) | |

**Note:**

1. World Bank region group: “EAP” represents “East Asia and Pacific”; “ECA” represents “Europe and Central Asia”; “LAC” represents “Latin America and the Caribbean”; “MENA” represents “Middle East and North Africa”; “SA” represents “South Asia”; “SSA” represents “Sub-Saharan Africa”.

2. Income group: “L” represents “Low-income country”; “LM” represents “Lower-middle income country”; “UM” represents “Upper-middle income country”.

*. The disparity in prevalence between young children living in the wealthiest and poorest quintiles is not statistically significant at the 0.05 level.

**Table S27.** National prevalence of young children living in households with adequate quarantine condition and associated disparities by place of residence (upper-bound)

| Country | Year | WB region^1^ | Income group^2^ | National (%) | Rural (%) | Urban (%) | Difference  (urban-rural) |
| --- | --- | --- | --- | --- | --- | --- | --- |
| Algeria | 2019 | MENA | UM | 88.3(87.3, 89.3) | 85.5(84.0, 87.1) | 90.4(89.1, 91.7) | 4.8(2.8, 6.9) |
| Angola | 2016 | SSA | UM | 73.8(71.9, 75.7) | 70.9(68.1, 73.7) | 75.7(73.1, 78.4) | 4.8(1.0, 8.7) |
| Armenia* | 2016 | ECA | LM | 97.4(96.4, 98.5) | 97.4(96.0, 98.9) | 97.4(96.0, 98.9) | 0.0(-2.1, 2.1) |
| Bangladesh* | 2019 | SA | LM | 90.3(89.7, 90.9) | 90.1(89.4, 90.8) | 90.9(89.6, 92.2) | 0.8(-0.6, 2.3) |
| Benin* | 2018 | SSA | L | 83.6(82.3, 85.0) | 82.8(81.0, 84.7) | 84.9(83.1, 86.7) | 2.0(-0.5, 4.6) |
| Burundi | 2017 | SSA | L | 94.6(93.9, 95.2) | 94.8(94.1, 95.5) | 92.4(90.2, 94.5) | -2.4(-4.7, -0.1) |
| CAR* | 2018 | SSA | L | 77.4(75.6, 79.3) | 76.6(74.6, 78.6) | 79.4(75.4, 83.5) | 2.8(-1.7, 7.4) |
| Cameroon* | 2018 | SSA | LM | 88.3(86.8, 89.8) | 89.1(86.9, 91.4) | 87.3(85.4, 89.2) | -1.8(-4.8, 1.1) |
| Chad* | 2019 | SSA | L | 81.9(80.8, 83.0) | 82.1(80.8, 83.3) | 81.3(79.1, 83.4) | -0.8(-3.3, 1.7) |
| Congo DR | 2018 | SSA | L | 83.5(81.8, 85.1) | 85.4(83.6, 87.2) | 80.4(77.6, 83.1) | -5.0(-8.3, -1.7) |
| Cote d’Ivoire | 2016 | SSA | LM | 82.6(80.8, 84.5) | 84.6(82.4, 86.9) | 79.5(76.4, 82.6) | -5.2(-9.0, -1.3) |
| Dominican Republic* | 2019 | LAC | UM | 93.4(92.3, 94.4) | 91.6(89.1, 94.1) | 94.0(92.8, 95.1) | 2.4(-0.4, 5.1) |
| Ethiopia | 2016 | SSA | L | 48.4(45.4, 51.3) | 45.2(42.1, 48.3) | 73.6(69.1, 78.1) | 28.4(22.9, 33.8) |
| Gambia* | 2020 | SSA | L | 93.8(92.5, 95.1) | 94.0(92.5, 95.5) | 93.6(91.8, 95.5) | -0.3(-2.7, 2.0) |
| Ghana* | 2017 | SSA | LM | 70.0(67.8, 72.2) | 71.0(68.3, 73.8) | 68.6(65.0, 72.1) | -2.5(-7.0, 2.0) |
| Guinea* | 2018 | SSA | L | 86.3(84.7, 87.8) | 87.1(85.2, 89.1) | 84.1(81.7, 86.5) | -3.0(-6.1, 0.1) |
| Guinea-Bissau | 2019 | SSA | L | 92.8(91.6, 94.0) | 96.1(95.1, 97.1) | 83.4(80.2, 86.6) | -12.7(-16.1, -9.4) |
| Guyana* | 2019 | LAC | UM | 88.8(86.4, 91.3) | 88.1(85.1, 91.2) | 90.9(87.3, 94.5) | 2.8(-1.9, 7.5) |
| Haiti | 2017 | LAC | L | 77.3(75.5, 79.2) | 78.9(76.5, 81.2) | 74.5(71.8, 77.3) | -4.3(-8.0, -0.6) |
| India | 2016 | SA | LM | 70.9(70.5, 71.3) | 69.1(68.7, 69.5) | 75.5(74.5, 76.5) | 6.4(5.3, 7.4) |
| Indonesia* | 2017 | EAP | LM | 93.2(92.6, 93.7) | 93.2(92.4, 94.0) | 93.1(92.4, 93.9) | 0.0(-1.1, 1.1) |
| Iraq | 2018 | MENA | UM | 76.4(74.6, 78.2) | 72.6(70.0, 75.2) | 78.2(75.8, 80.5) | 5.5(2.1, 9.0) |
| Kiribati* | 2018 | EAP | LM | 67.7(64.6, 70.8) | 70.9(67.3, 74.5) | 64.9(60.1, 69.7) | -6.0(-12.0, 0.1) |
| Kyrgyzstan* | 2018 | ECA | LM | 93.2(91.7, 94.6) | 92.9(91.0, 94.7) | 93.8(91.4, 96.3) | 1.0(-2.1, 4.0) |
| Lao | 2017 | EAP | LM | 66.9(65.3, 68.6) | 61.1(59.1, 63.1) | 82.5(80.2, 84.9) | 21.4(18.3, 24.6) |
| Lesotho | 2018 | SSA | LM | 76.7(74.5, 78.9) | 71.8(68.8, 74.7) | 86.5(83.8, 89.1) | 14.7(10.8, 18.6) |
| Liberia | 2019 | SSA | L | 84.9(82.7, 87.0) | 88.4(86.6, 90.2) | 81.8(78.0, 85.5) | -6.6(-10.8, -2.4) |
| Madagascar | 2018 | SSA | L | 60.8(59.3, 62.4) | 59.3(57.6, 61.1) | 66.8(63.6, 69.9) | 7.4(3.8, 11.0) |
| Malawi | 2020 | SSA | L | 92.1(91.4, 92.9) | 91.6(90.8, 92.5) | 95.6(93.9, 97.2) | 3.9(2.1, 5.8) |
| Maldives | 2017 | SA | UM | 93.6(91.7, 95.4) | 96.6(95.6, 97.7) | 88.0(83.2, 92.7) | -8.7(-13.6, -3.8) |
| Mali* | 2018 | SSA | L | 89.4(88.2, 90.6) | 89.2(87.8, 90.7) | 90.1(88.1, 92.0) | 0.8(-1.6, 3.2) |
| Mongolia | 2018 | EAP | LM | 76.5(73.4, 79.6) | 68.9(63.4, 74.4) | 78.4(74.9, 82.0) | 9.6(3.0, 16.1) |
| Myanmar* | 2016 | EAP | LM | 68.5(66.2, 70.8) | 67.5(64.8, 70.1) | 72.1(67.6, 76.6) | 4.7(-0.6, 9.9) |
| Nepal | 2019 | SA | L | 89.2(87.7, 90.6) | 87.0(84.8, 89.1) | 90.4(88.5, 92.3) | 3.4(0.5, 6.3) |
| Nigeria* | 2018 | SSA | LM | 80.7(79.7, 81.7) | 81.5(80.1, 82.8) | 79.6(78.1, 81.0) | -1.9(-3.9, 0.1) |
| PNG* | 2016-18 | SA | LM | 80.6(78.8, 82.3) | 80.4(78.5, 82.4) | 81.7(78.0, 85.4) | 1.2(-2.9, 5.4) |
| Pakistan | 2018 | EAP | LM | 56.6(54.1, 59.2) | 53.0(49.8, 56.3) | 64.3(60.6, 68.1) | 11.3(6.3, 16.3) |
| Palestine* | 2019 | MENA | LM | 88.9(87.4, 90.3) | 87.7(85.4, 90.1) | 89.3(87.5, 91.0) | 1.5(-1.4, 4.5) |
| Paraguay | 2016 | LAC | UM | 89.2(87.2, 91.3) | 86.0(82.9, 89.1) | 91.4(88.8, 94.1) | 5.4(1.3, 9.5) |
| Philippines* | 2017 | EAP | LM | 72.3(70.4, 74.2) | 72.4(70.2, 74.6) | 72.1(68.8, 75.4) | -0.3(-4.3, 3.7) |
| Rwanda* | 2020 | SSA | L | 95.8(95.1, 96.4) | 95.7(95.0, 96.5) | 96.0(94.5, 97.4) | 0.2(-1.4, 1.8) |
| Samoa | 2019 | EAP | L | 59.8(55.9, 63.8) | 57.6(53.1, 62.0) | 71.6(65.7, 77.6) | 14.1(6.6, 21.6) |
| STP* | 2019 | SSA | LM | 87.5(85.1, 89.9) | 87.0(83.7, 90.2) | 87.7(84.5, 91.0) | 0.7(-3.9, 5.3) |
| Senegal* | 2019 | SSA | L | 89.8(88.2, 91.5) | 89.7(87.7, 91.8) | 89.9(87.2, 92.6) | 0.2(-3.3, 3.6) |
| Sierra Leone | 2019 | SSA | L | 91.0(90.0, 91.9) | 92.3(91.2, 93.4) | 88.4(86.6, 90.3) | -3.9(-6.1, -1.7) |
| South Africa* | 2016 | SSA | UM | 90.1(88.2, 92.1) | 89.0(86.1, 92.0) | 90.7(88.2, 93.3) | 1.7(-2.2, 5.6) |
| Suriname | 2018 | LAC | UM | 86.7(84.9, 88.5) | 77.2(73.8, 80.7) | 91.6(89.7, 93.5) | 14.4(10.4, 18.3) |
| Tajikistan* | 2017 | ECA | LM | 90.3(88.8, 91.8) | 89.8(88.0, 91.6) | 92.2(90.0, 94.4) | 2.4(-0.4, 5.2) |
| Timor-Leste* | 2016 | EAP | LM | 88.4(87.0, 89.8) | 87.9(86.4, 89.4) | 89.7(86.4, 92.9) | 1.8(-1.8, 5.4) |
| Togo | 2017 | SSA | L | 85.5(83.7, 87.3) | 87.2(85.0, 89.3) | 82.8(79.5, 86.1) | -4.4(-8.3, -0.4) |
| Tonga* | 2019 | EAP | LM | 88.9(85.9, 91.9) | 88.8(85.2, 92.4) | 89.2(85.4, 93.1) | 0.4(-4.9, 5.8) |
| Tunisia | 2018 | MENA | LM | 94.0(92.9, 95.1) | 91.5(89.4, 93.5) | 95.4(94.1, 96.7) | 4.0(1.5, 6.4) |
| Tuvalu* | 2019 | EAP | UM | 69.9(63.9, 75.9) | 66.8(59.0, 74.6) | 71.5(63.4, 79.6) | 4.6(-6.8, 16.1) |
| Uganda | 2016 | SSA | L | 77.3(75.9, 78.6) | 76.3(74.8, 77.9) | 80.8(78.3, 83.4) | 4.5(1.5, 7.5) |
| Zambia | 2018 | SSA | LM | 82.9(81.7, 84.1) | 80.9(79.5, 82.4) | 86.4(84.4, 88.4) | 5.5(3.0, 7.9) |
| Zimbabwe | 2019 | SSA | L | 90.1(88.8, 91.3) | 89.1(87.6, 90.7) | 92.3(90.3, 94.3) | 3.1(0.6, 5.7) |

**Note:**

1. World Bank region group: “EAP” represents “East Asia and Pacific”; “ECA” represents “Europe and Central Asia”; “LAC” represents “Latin America and the Caribbean”; “MENA” represents “Middle East and North Africa”; “SA” represents “South Asia”; “SSA” represents “Sub-Saharan Africa”.

2. Income group: “L” represents “Low-income country”; “LM” represents “Lower-middle income country”; “UM” represents “Upper-middle income country”.

*. The disparity in prevalence between young children in urban and rural areas is not statistically significant at the 0.05 level.

**Table S28.** National prevalence of young children living in households with adequate quarantine condition and associated disparities by household wealth quintile (upper-bound)

| **Country** | **Year** | **WB region^1^** | **Income group^2^** | **Wealth quintile** | | | | | **Difference (richest-poorest)** |
| --- | --- | --- | --- | --- | --- | --- | --- | --- | --- |
|  |  |  |  | **Poorest (%)** | **2 (%)** | **3 (%)** | **4 (%)** | **Richest (%)** |  |
| Algeria | 2019 | MENA | UM | 76.7(74.5, 79.0) | 84.9(82.8, 86.9) | 92.3(90.6, 93.9) | 96.3(95.2, 97.5) | 97.3(95.3, 99.2) | 20.5(17.5, 23.6) |
| Angola | 2016 | SSA | UM | 68.0(64.6, 71.4) | 73.5(70.7, 76.4) | 69.8(66.2, 73.5) | 73.8(68.9, 78.6) | 89.9(86.8, 93.0) | 21.9(17.3, 26.5) |
| Armenia | 2016 | ECA | LM | 95.4(92.8, 98.1) | 98.3(96.0, 100.6) | 95.9(92.2, 99.6) | 97.7(95.7, 99.6) | 99.3(98.2, 100.3) | 3.8(1.0, 6.7) |
| Bangladesh | 2019 | SA | LM | 80.3(78.6, 81.9) | 88.3(87.0, 89.6) | 93.8(92.7, 94.8) | 94.0(92.9, 95.1) | 96.0(94.9, 97.1) | 15.7(13.8, 17.7) |
| Benin | 2018 | SSA | L | 77.3(74.2, 80.5) | 83.5(81.2, 85.7) | 82.8(80.1, 85.5) | 85.6(83.4, 87.9) | 90.1(88.0, 92.2) | 12.8(9.0, 16.6) |
| Burundi | 2017 | SSA | L | 91.7(90.2, 93.3) | 95.3(94.1, 96.5) | 95.2(94.0, 96.5) | 95.9(94.3, 97.4) | 95.1(93.5, 96.6) | 3.3(1.1, 5.6) |
| CAR | 2018 | SSA | L | 69.7(65.5, 73.8) | 74.8(70.9, 78.6) | 77.3(74.0, 80.7) | 81.6(78.2, 85.0) | 88.5(85.7, 91.3) | 18.8(13.9, 23.8) |
| Cameroon | 2018 | SSA | LM | 84.1(79.6, 88.5) | 91.5(89.4, 93.6) | 86.3(83.5, 89.0) | 87.2(84.0, 90.3) | 94.0(92.0, 96.0) | 10.0(5.1, 14.8) |
| Chad | 2019 | SSA | L | 69.5(67.1, 71.8) | 78.7(76.4, 81.0) | 86.0(84.2, 87.9) | 90.8(88.8, 92.8) | 86.8(85.0, 88.7) | 17.4(14.4, 20.3) |
| Congo DR* | 2018 | SSA | L | 79.8(76.7, 83.0) | 85.2(82.8, 87.5) | 88.8(87.1, 90.6) | 81.9(78.6, 85.2) | 81.6(77.2, 86.0) | 1.8(-3.7, 7.2) |
| Cote d’Ivoire* | 2016 | SSA | LM | 82.7(80.0, 85.5) | 84.1(81.0, 87.2) | 84.3(81.1, 87.5) | 76.7(72.3, 81.1) | 85.3(80.3, 90.3) | 2.6(-3.1, 8.3) |
| Dominican Republic | 2019 | LAC | UM | 85.8(83.3, 88.3) | 89.7(86.8, 92.6) | 97.4(96.1, 98.8) | 97.6(95.9, 99.3) | 99.7(99.2, 100.2) | 13.9(11.4, 16.4) |
| Ethiopia | 2016 | SSA | L | 32.7(29.0, 36.4) | 39.1(35.1, 43.2) | 46.5(40.1, 52.8) | 63.2(58.6, 67.8) | 73.0(68.7, 77.3) | 40.3(34.5, 46.0) |
| Gambia* | 2020 | SSA | L | 91.6(89.2, 94.0) | 92.5(90.1, 94.9) | 95.8(93.6, 97.9) | 94.0(90.9, 97.1) | 95.5(91.9, 99.2) | 4.0(-0.5, 8.4) |
| Ghana | 2017 | SSA | LM | 68.2(64.2, 72.3) | 63.1(57.8, 68.3) | 66.5(62.2, 70.8) | 67.5(62.7, 72.3) | 86.2(82.9, 89.5) | 18.0(12.7, 23.2) |
| Guinea | 2018 | SSA | L | 79.4(76.0, 82.9) | 90.2(87.6, 92.9) | 91.1(88.5, 93.7) | 83.0(80.1, 85.9) | 89.1(86.7, 91.5) | 9.7(5.5, 13.9) |
| Guinea-Bissau | 2019 | SSA | L | 93.3(90.8, 95.8) | 96.7(94.8, 98.7) | 97.5(96.0, 99.0) | 88.4(85.4, 91.4) | 83.5(79.5, 87.5) | -9.8(-14.5, -5.1) |
| Guyana | 2019 | LAC | UM | 73.8(69.0, 78.5) | 90.5(86.0, 95.1) | 92.6(84.7, 100.6) | 98.3(96.5, 100.2) | 98.4(96.1, 100.7) | 24.6(19.3, 29.9) |
| Haiti | 2017 | LAC | L | 71.8(67.9, 75.7) | 81.7(78.3, 85.1) | 73.3(68.8, 77.8) | 75.9(71.3, 80.4) | 88.8(85.8, 91.9) | 17.1(12.1, 22.0) |
| India | 2016 | SA | LM | 55.1(54.4, 55.7) | 66.1(65.4, 66.9) | 72.7(71.9, 73.5) | 80.1(78.9, 81.3) | 91.4(90.6, 92.2) | 36.3(35.3, 37.4) |
| Indonesia | 2017 | EAP | LM | 84.1(82.4, 85.8) | 93.1(91.9, 94.3) | 94.5(93.5, 95.5) | 96.5(95.6, 97.3) | 98.1(97.2, 99.0) | 14.0(12.0, 15.9) |
| Iraq | 2018 | MENA | UM | 61.3(57.8, 64.9) | 75.8(72.1, 79.6) | 77.5(72.8, 82.3) | 84.8(82.0, 87.6) | 86.5(82.9, 90.0) | 25.2(20.1, 30.2) |
| Kiribati | 2018 | EAP | LM | 64.9(59.4, 70.4) | 68.6(62.5, 74.7) | 64.0(55.6, 72.3) | 64.0(56.7, 71.2) | 78.3(71.4, 85.2) | 13.4(4.5, 22.3) |
| Kyrgyzstan | 2018 | ECA | LM | 85.0(80.5, 89.6) | 95.6(93.2, 97.9) | 97.6(96.2, 99.0) | 95.1(92.6, 97.7) | 94.1(90.2, 98.1) | 9.1(3.1, 15.1) |
| Lao | 2017 | EAP | LM | 44.2(41.2, 47.3) | 55.3(52.2, 58.4) | 70.6(67.7, 73.6) | 84.1(81.4, 86.8) | 94.5(93.0, 95.9) | 50.2(46.8, 53.6) |
| Lesotho | 2018 | SSA | LM | 66.4(62.0, 70.9) | 68.9(63.7, 74.0) | 74.0(68.5, 79.5) | 88.3(84.6, 92.0) | 91.0(87.0, 94.9) | 24.5(18.5, 30.6) |
| Liberia | 2019 | SSA | L | 85.7(83.2, 88.2) | 90.9(88.4, 93.4) | 92.1(89.1, 95.1) | 75.7(68.5, 82.9) | 77.8(70.9, 84.8) | -7.8(-15.2, -0.4) |
| Madagascar | 2018 | SSA | L | 46.8(44.4, 49.3) | 52.7(49.9, 55.5) | 63.0(60.0, 66.0) | 75.1(72.0, 78.1) | 79.2(75.5, 82.9) | 32.4(28.0, 36.8) |
| Malawi | 2020 | SSA | L | 86.8(85.1, 88.5) | 90.7(88.8, 92.6) | 94.7(93.3, 96.0) | 94.1(92.6, 95.6) | 97.8(96.7, 98.8) | 10.9(8.9, 13.0) |
| Maldives* | 2017 | SA | UM | 93.5(90.7, 96.3) | 96.2(94.0, 98.4) | 94.4(90.8, 98.1) | 88.5(81.8, 95.3) | 94.4(88.8, 100.0) | 0.9(-5.4, 7.2) |
| Mali | 2018 | SSA | L | 86.8(84.0, 89.7) | 88.7(86.1, 91.2) | 89.8(87.3, 92.3) | 87.9(85.4, 90.4) | 94.8(93.2, 96.4) | 8.0(4.7, 11.2) |
| Mongolia | 2018 | EAP | LM | 67.6(58.0, 77.1) | 57.2(50.1, 64.2) | 61.9(55.3, 68.5) | 79.9(74.8, 84.9) | 91.4(88.0, 94.9) | 23.8(13.7, 34.0) |
| Myanmar | 2016 | EAP | LM | 62.5(58.3, 66.8) | 63.7(59.4, 68.0) | 66.4(61.5, 71.3) | 74.9(70.3, 79.4) | 83.3(78.9, 87.7) | 20.8(14.5, 27.0) |
| Nepal | 2019 | SA | L | 81.5(78.5, 84.5) | 86.6(83.0, 90.1) | 89.3(86.0, 92.6) | 94.4(92.4, 96.4) | 97.0(95.6, 98.4) | 15.6(12.3, 18.9) |
| Nigeria | 2018 | SSA | LM | 78.9(76.8, 81.0) | 78.9(76.4, 81.5) | 82.6(80.7, 84.6) | 79.6(77.6, 81.6) | 84.7(82.6, 86.9) | 5.8(2.8, 8.8) |
| PNG | 2016-18 | SA | LM | 73.4(69.3, 77.5) | 76.7(73.1, 80.4) | 85.1(82.3, 87.9) | 82.0(79.0, 84.9) | 86.3(82.2, 90.4) | 12.9(7.1, 18.7) |
| Pakistan | 2018 | EAP | LM | 30.6(27.1, 34.0) | 49.5(44.7, 54.2) | 55.1(49.9, 60.2) | 68.6(63.9, 73.3) | 87.5(84.3, 90.8) | 56.9(52.2, 61.7) |
| Palestine | 2019 | MENA | LM | 75.2(71.0, 79.4) | 90.0(87.4, 92.7) | 90.8(88.5, 93.1) | 94.2(92.3, 96.2) | 97.4(95.8, 99.0) | 22.2(17.7, 26.7) |
| Paraguay | 2016 | LAC | UM | 71.7(66.2, 77.1) | 89.7(85.8, 93.5) | 95.9(93.8, 98.0) | 97.0(95.0, 98.9) | 99.3(98.7, 99.9) | 27.6(22.1, 33.1) |
| Philippines | 2017 | EAP | LM | 56.4(53.3, 59.5) | 68.7(65.1, 72.3) | 76.3(72.3, 80.2) | 84.5(81.5, 87.5) | 90.5(84.8, 96.2) | 34.1(27.6, 40.6) |
| Rwanda | 2020 | SSA | L | 89.6(87.6, 91.7) | 97.6(96.6, 98.7) | 97.2(96.0, 98.4) | 97.2(96.1, 98.2) | 98.7(97.7, 99.8) | 9.1(6.8, 11.4) |
| Samoa | 2019 | EAP | L | 30.3(23.5, 37.0) | 48.5(42.2, 54.9) | 65.9(57.4, 74.4) | 78.5(72.1, 84.8) | 91.2(85.8, 96.6) | 60.9(52.2, 69.7) |
| STP | 2019 | SSA | LM | 80.2(74.4, 86.1) | 82.0(75.9, 88.0) | 87.2(82.6, 91.8) | 92.8(88.5, 97.0) | 99.0(97.1, 100.8) | 18.7(12.6, 24.8) |
| Senegal | 2019 | SSA | L | 84.1(80.3, 88.0) | 89.5(86.1, 92.9) | 88.3(83.2, 93.5) | 93.9(90.8, 97.1) | 95.4(92.2, 98.6) | 11.3(6.2, 16.3) |
| Sierra Leone* | 2019 | SSA | L | 90.4(88.5, 92.3) | 93.1(91.5, 94.8) | 93.3(91.6, 95.1) | 88.3(85.9, 90.6) | 88.6(85.6, 91.7) | -1.8(-5.4, 1.8) |
| South Africa | 2016 | SSA | UM | 81.2(75.8, 86.6) | 92.4(89.7, 95.0) | 90.8(87.2, 94.4) | 90.2(84.4, 96.1) | 99.5(98.7, 100.2) | 18.2(12.7, 23.7) |
| Suriname | 2018 | LAC | UM | 71.1(66.5, 75.7) | 87.5(83.6, 91.3) | 95.5(92.9, 98.1) | 96.5(93.5, 99.5) | 97.6(94.2, 101.1) | 26.5(20.8, 32.3) |
| Tajikistan | 2017 | ECA | LM | 83.0(79.2, 86.8) | 88.4(84.8, 91.9) | 91.2(87.4, 94.9) | 96.2(94.7, 97.7) | 92.2(89.5, 94.8) | 9.2(4.5, 13.8) |
| Timor-Leste | 2016 | EAP | LM | 74.9(70.7, 79.1) | 89.7(87.3, 92.1) | 92.5(90.3, 94.6) | 91.6(88.6, 94.6) | 93.6(90.6, 96.7) | 18.8(13.6, 24.0) |
| Togo | 2017 | SSA | L | 85.3(80.7, 89.9) | 88.2(84.9, 91.5) | 81.8(78.0, 85.5) | 79.9(74.9, 85.0) | 93.0(90.3, 95.7) | 7.7(2.3, 13.0) |
| Tonga | 2019 | EAP | LM | 71.1(61.6, 80.5) | 90.4(84.5, 96.4) | 95.4(91.0, 99.8) | 92.4(86.8, 98.1) | 99.3(98.0, 100.7) | 28.2(18.6, 37.9) |
| Tunisia | 2018 | MENA | LM | 87.8(84.8, 90.8) | 93.1(90.7, 95.5) | 92.7(90.0, 95.4) | 98.2(96.9, 99.6) | 98.2(96.6, 99.9) | 10.4(7.0, 13.8) |
| Tuvalu | 2019 | EAP | UM | 46.7(33.0, 60.3) | 63.5(50.8, 76.3) | 67.6(56.0, 79.1) | 85.6(75.0, 96.3) | 88.7(79.7, 97.8) | 42.0(24.8, 59.3) |
| Uganda | 2016 | SSA | L | 61.4(58.5, 64.3) | 75.8(73.3, 78.3) | 82.3(79.7, 84.9) | 84.1(81.5, 86.8) | 86.1(83.5, 88.7) | 24.7(20.8, 28.7) |
| Zambia | 2018 | SSA | LM | 75.3(72.6, 78.0) | 81.5(79.0, 83.9) | 87.1(84.6, 89.6) | 85.7(82.6, 88.7) | 88.7(84.3, 93.0) | 13.4(8.3, 18.5) |
| Zimbabwe | 2019 | SSA | L | 82.6(80.1, 85.1) | 89.6(86.4, 92.8) | 93.2(91.0, 95.5) | 91.0(88.8, 93.2) | 96.4(94.3, 98.5) | 13.7(10.5, 17.0) |

**Note:**

1. World Bank region group: “EAP” represents “East Asia and Pacific”; “ECA” represents “Europe and Central Asia”; “LAC” represents “Latin America and the Caribbean”; “MENA” represents “Middle East and North Africa”; “SA” represents “South Asia”; “SSA” represents “Sub-Saharan Africa”.

2. Income group: “L” represents “Low-income country”; “LM” represents “Lower-middle income country”; “UM” represents “Upper-middle income country”.

*. The disparity in prevalence between young children living in the wealthiest and poorest quintiles is not statistically significant at the 0.05 level.

*(3) Additional figures*

**Figure S1.** Country-level percentage of young children living in households with preparedness (lower-bound)


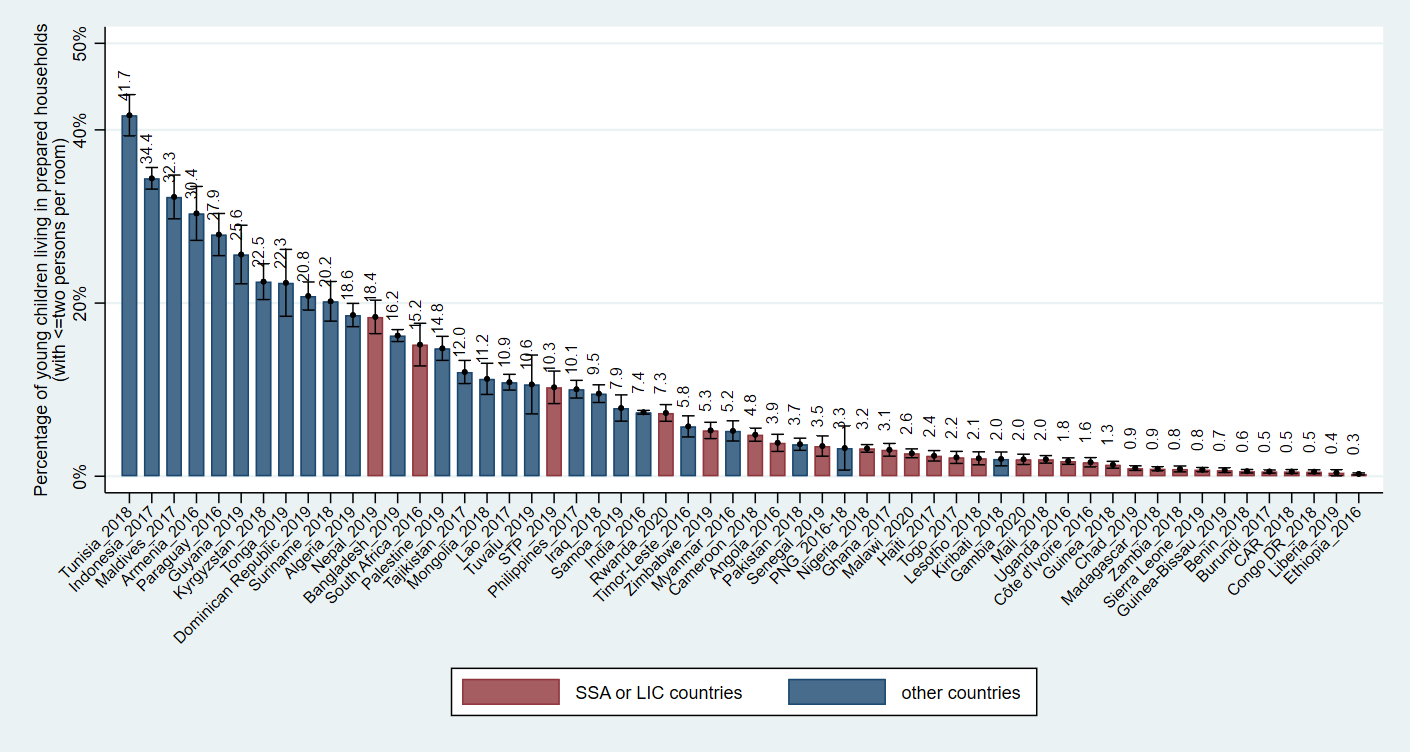


**Figure S2.** Country-level percentage of young children living in households with preparedness (upper-bound)


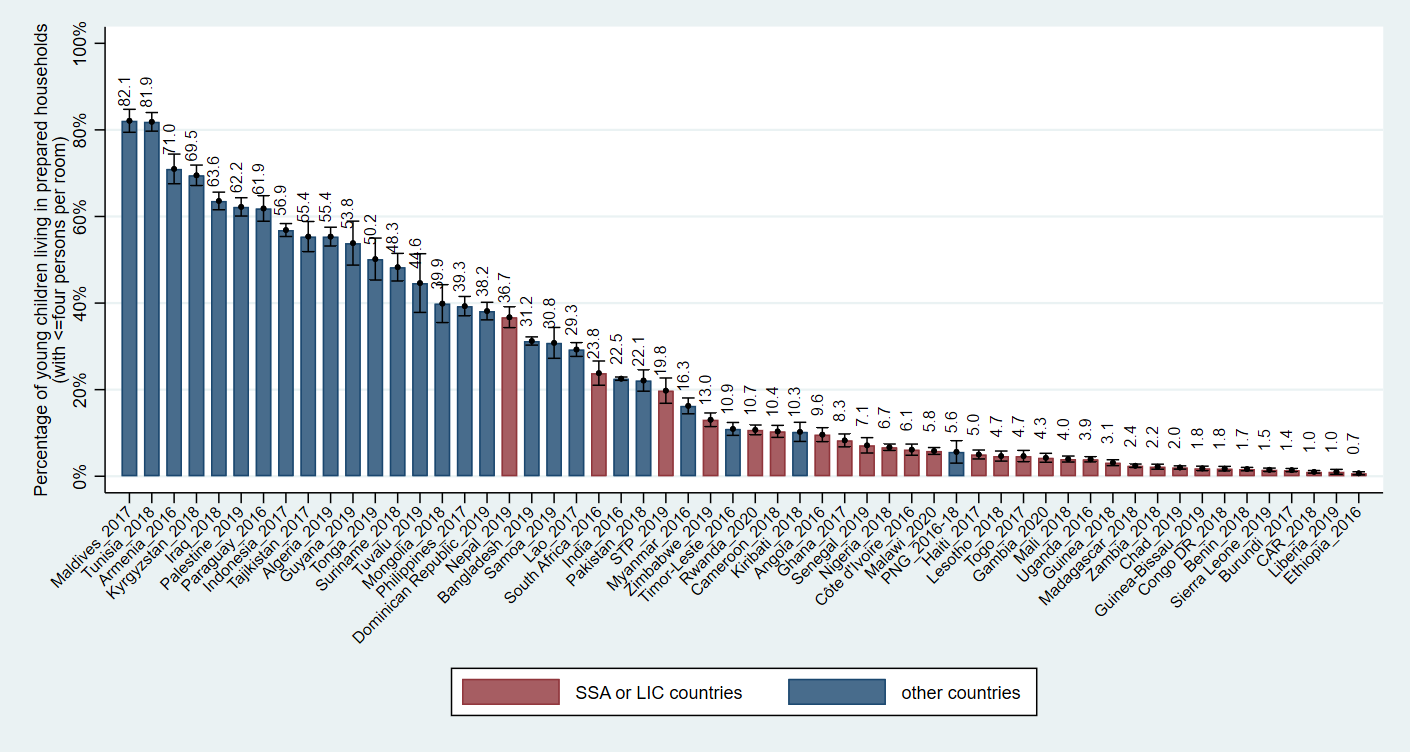


**Figure S3.** Country-level percentage of young children living in households with adequate quarantine condition


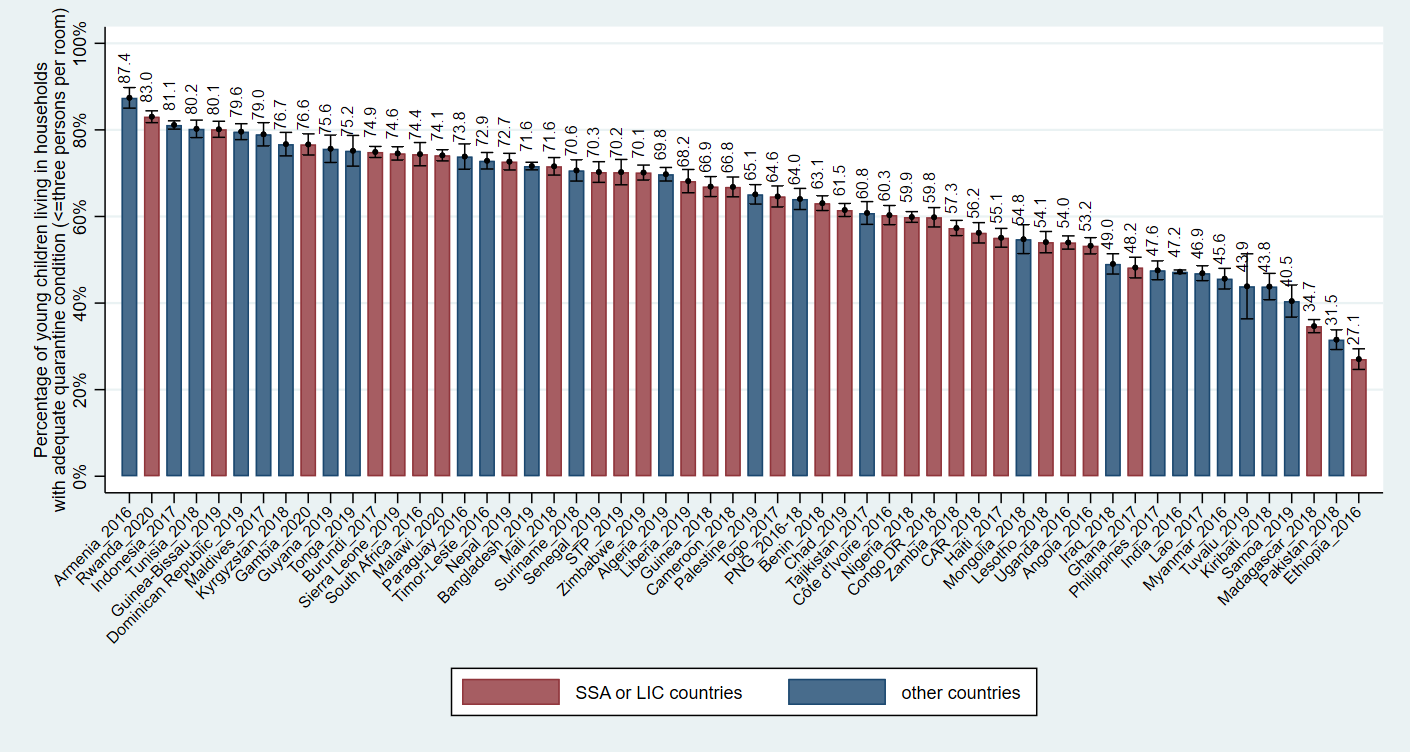


**Figure S4.** Country-level percentage of young children living in households with adequate quarantine condition (lower-bound)


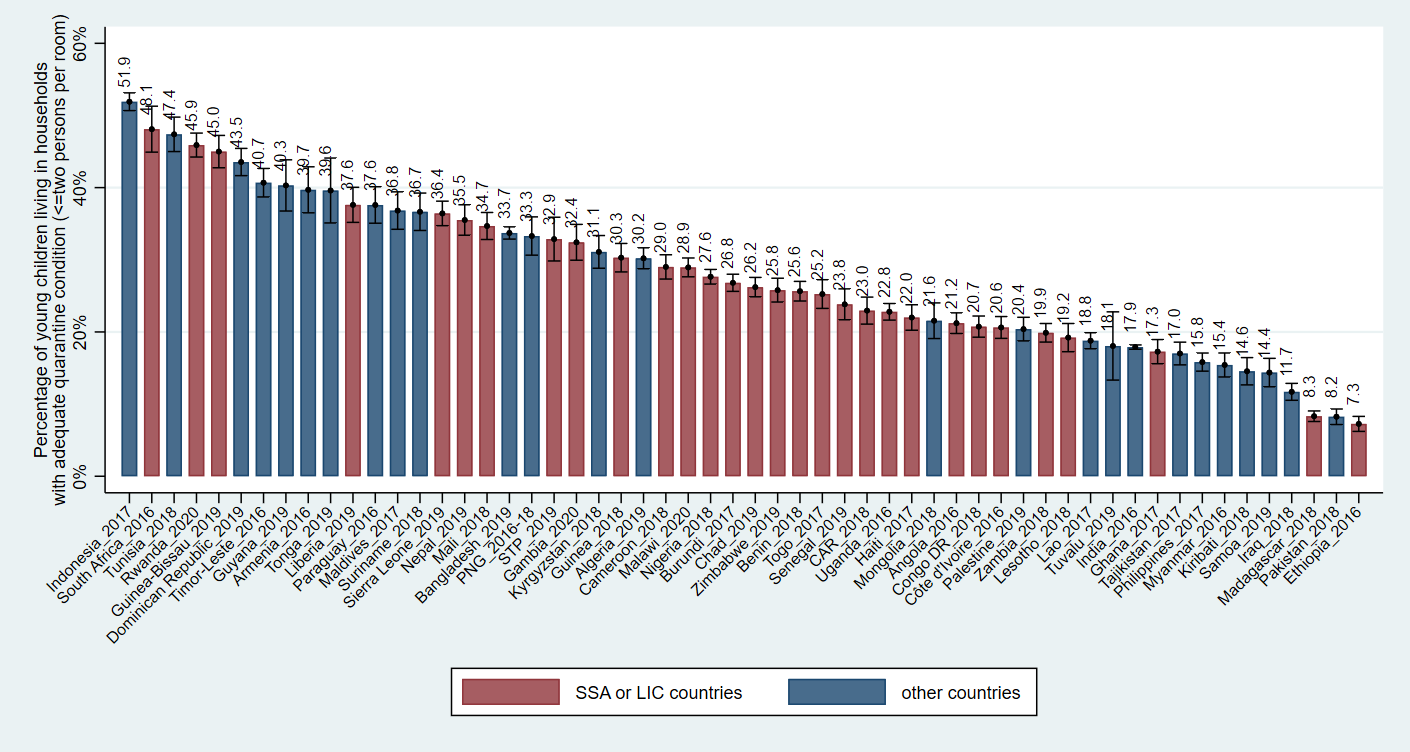


**Figure S5.** Country-level percentage of young children living in households with adequate quarantine condition (upper-bound)


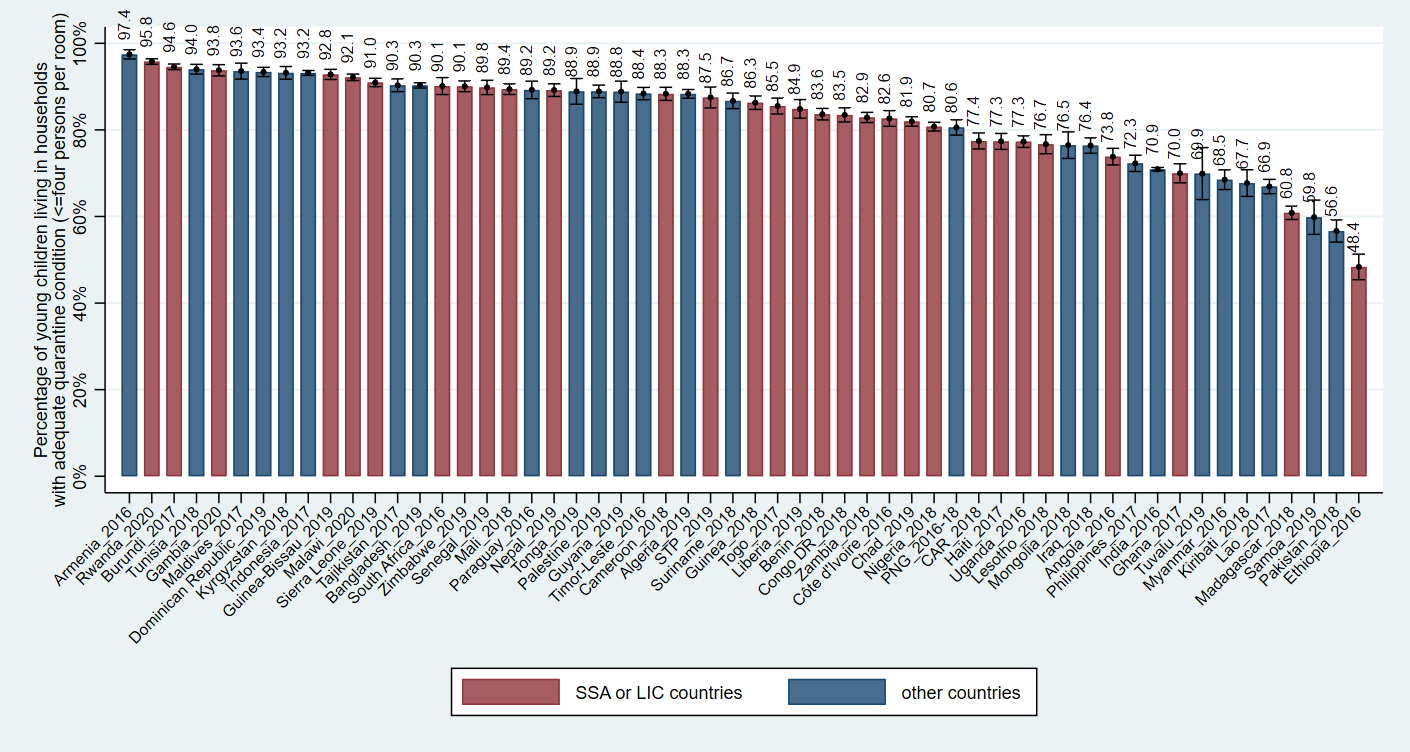


**Figure S6.** Country-level percentage of young children living in households with basic hygiene conditions


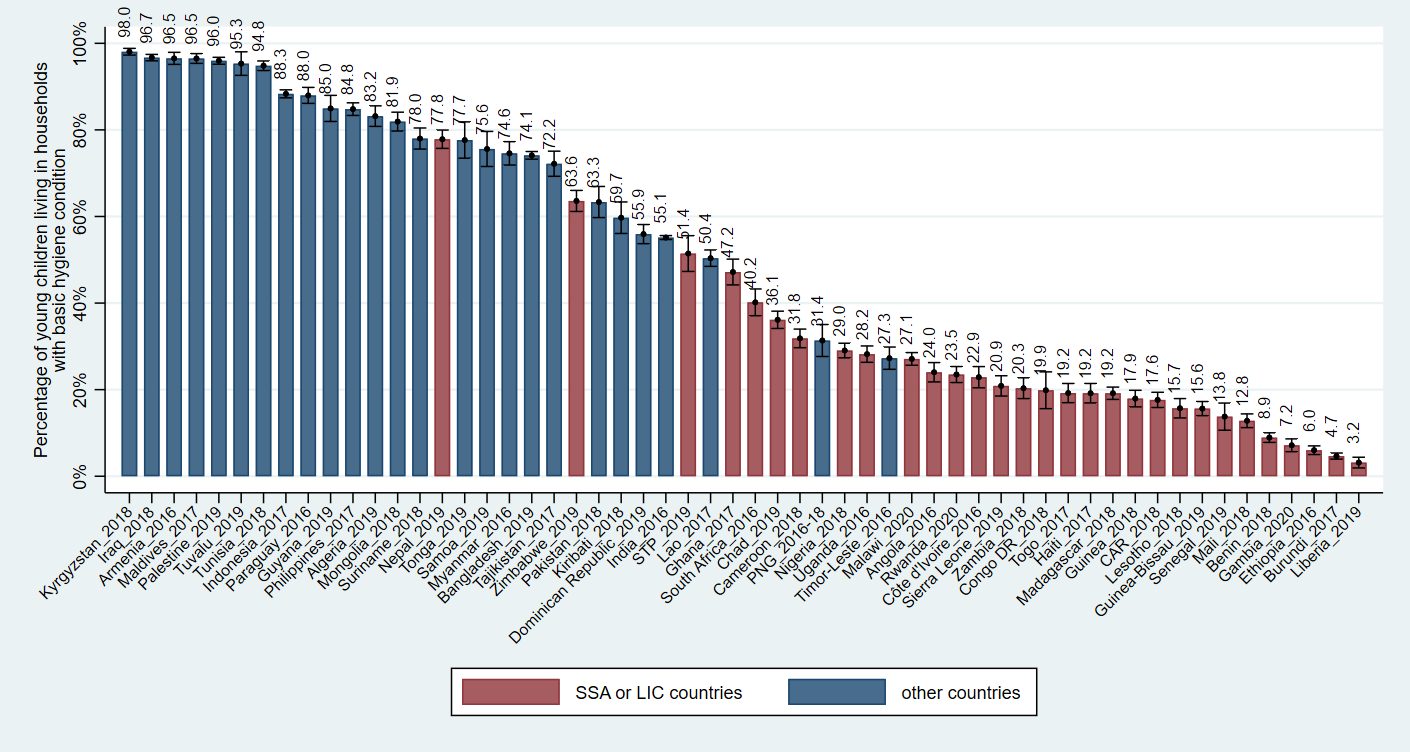


**Figure S7.** Country-level percentage of young children living in households with basic sanitation conditions


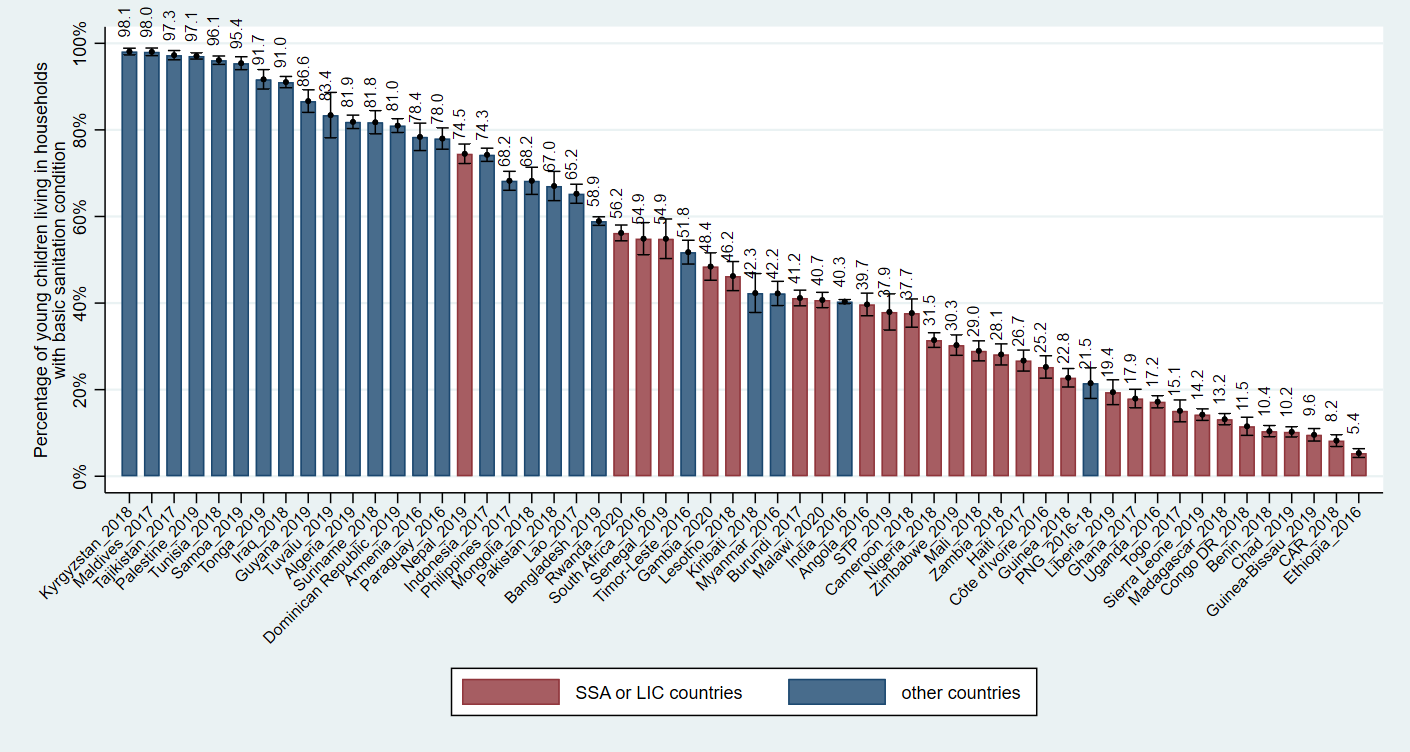


**Figure S8.** Country-level percentage of young children living in households with ownership of landline or mobile phones


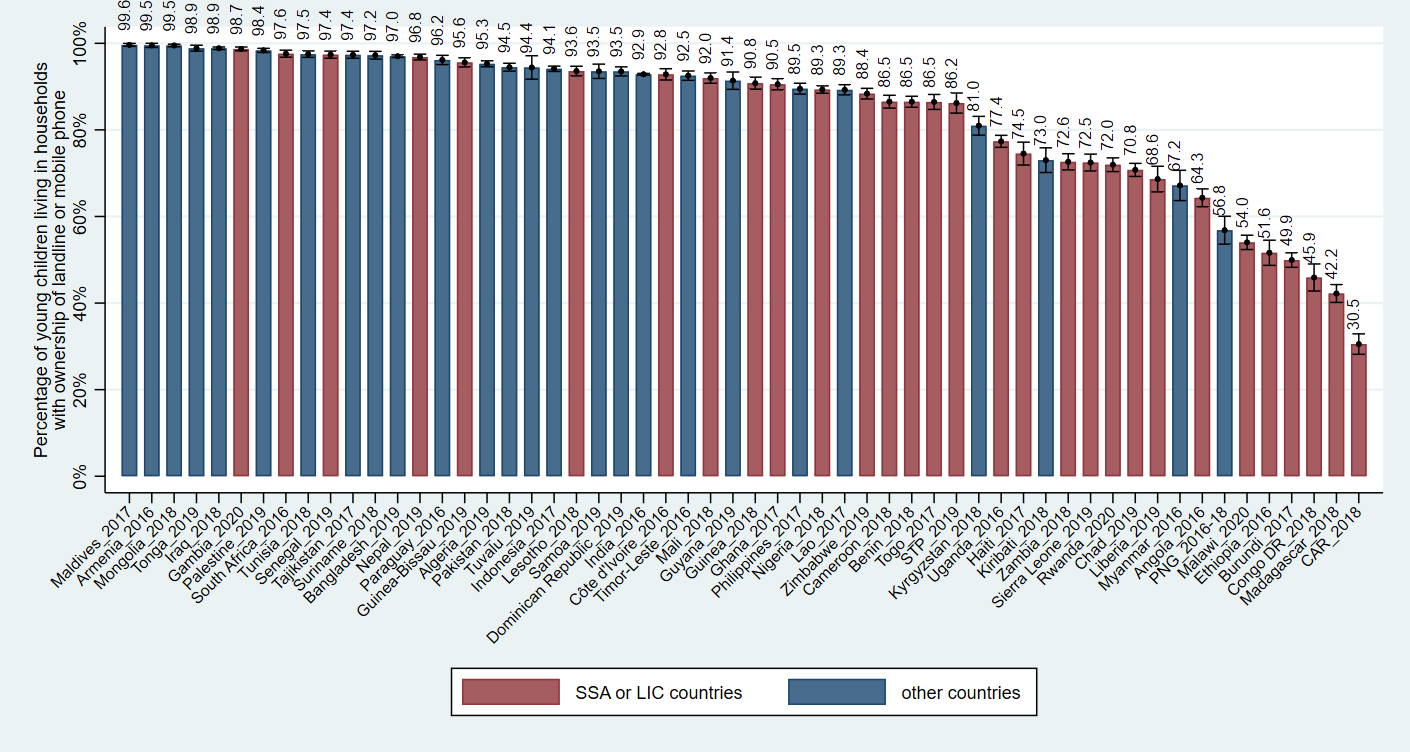


**Figure S9.** Country-level percentage of young children living in households with mother exposed to mass media at least once a week


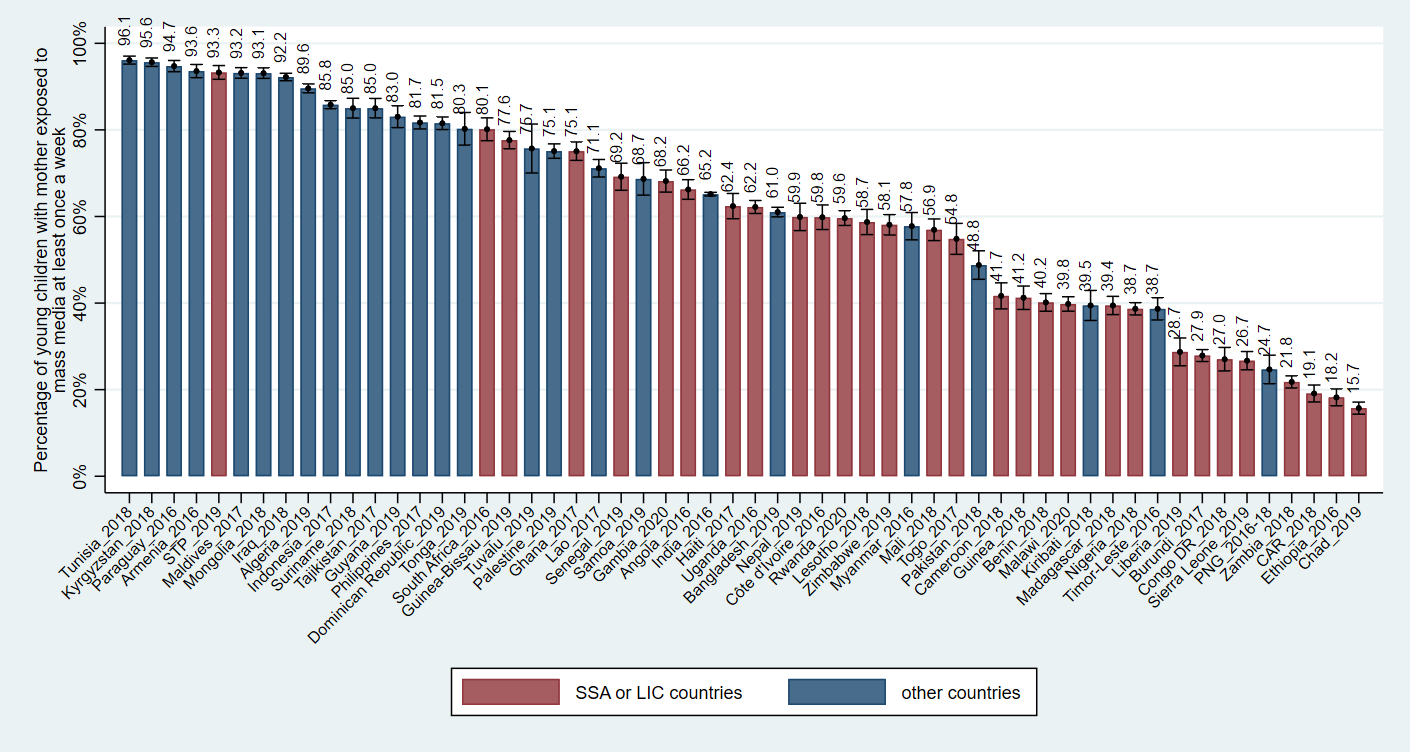


1. Both DHS and MICS use two separate files to record household and individual women’s survey data; sample size here reflects the number of successfully surveyed households and women. [↑](#footnote-ref-2)
2. The percentage of missing values is calculated as the number of households with missing values in that variable divided by the household sample size. [↑](#footnote-ref-3)
3. The percentage of missing values is calculated as the number of women with missing values divided by the woman sample size. [↑](#footnote-ref-4)
